# Hydrothermal polymerization of porous aromatic polyimide networks and machine learning-assisted computational morphology evolution interpretation†

**DOI:** 10.1039/d1ta01253c

**Published:** 2021-09-08

**Authors:** Marianne Lahnsteiner, Michael Caldera, Hipassia M. Moura, D. Alonso Cerrón-Infantes, Jérôme Roeser, Thomas Konegger, Arne Thomas, Jörg Menche, Miriam M. Unterlass

**Affiliations:** Technische Universität Wien, Institute of Materials Chemistry Getreidemarkt 9/165 1060 Vienna Austria; Technische Universität Wien, Institute of Applied Synthetic Chemistry Getreidemarkt 9/163 1060 Vienna Austria; CeMM – Research Center for Molecular Medicine of the Austrian Academy of Sciences Lazarettgasse 14, AKH BT 25.3 1090 Vienna Austria; Max F. Perutz Labs, Campus Vienna Biocenter 5 Dr.-Bohr-Gasse 9 1030 Vienna Austria; Universität Konstanz, Department of Chemistry, Solid State Chemistry Universitätsstrasse 10 D-78464 Konstanz Germany miriam.unterlass@uni-konstanz.de; Technische Universität Berlin, Institute of Chemistry Str. des 17. Juni 115 10623 Berlin Germany; Technische Universität Wien, Institute of Chemical Technologies and Analytics Getreidemarkt 9/164 1060 Vienna Austria

## Abstract

We report on the hydrothermal polymerization (HTP) of polyimide (PI) networks using the medium H_2_O and the comonomers 1,3,5-tris(4-aminophenyl)benzene (TAPB) and pyromellitic acid (PMA). Full condensation is obtained at minimal reaction times of only 2 h at 200 °C. The PI networks are obtained as monoliths and feature thermal stabilities of >500 °C, and in several cases even up to 595 °C. The monoliths are built up by networks of densely packed, near-monodisperse spherical particles and annealed microfibers, and show three types of porosity: (i) intrinsic inter-segment ultramicroporosity (<0.8 nm) of the PI networks composing the particles (∼3–5 μm), (ii) interstitial voids between the particles (0.1–2 μm), and (iii) monolith cell porosity (∽10–100 μm), as studied *via* low pressure gas physisorption and Hg intrusion porosimetry analyses. This unique hierarchical porosity generates an outstandingly high specific pore volume of 7250 mm^3^ g^−1^. A large-scale micromorphological study screening the reaction parameters time, temperature, and the absence/presence of the additive acetic acid was performed. Through expert interpretation of hundreds of scanning electron microscopy (SEM) images of the products of these experiments, we devise a hypothesis for morphology formation and evolution: a monomer salt is initially formed and subsequently transformed to overall eight different fiber, pearl chain, and spherical morphologies, composed of PI and, at long reaction times (>48 h), also PI/SiO_2_ hybrids that form through reaction with the reaction vessel. Moreover, we have developed a computational image analysis pipeline that deciphers the complex morphologies of these SEM images automatically and also allows for formulating a hypothesis of morphology development in HTP that is in good agreement with the manual morphology analysis. Finally, we upscaled the HTP of PI(TAPB–PMA) and processed the resulting powder into dense cylindrical specimen by green solvent-free warm-pressing, showing that one can follow the full route from the synthesis of these PI networks to a final material without employing harmful solvents.

## Abstract 2

We report on the hydrothermal polymerization (HTP) of porous polyimide (PI) networks using the medium H_2_O and the comonomers 1,3,5-tris(4-aminophenyl)benzene (TAPB) and pyromellitic acid (PMA).

## Graphical abstract

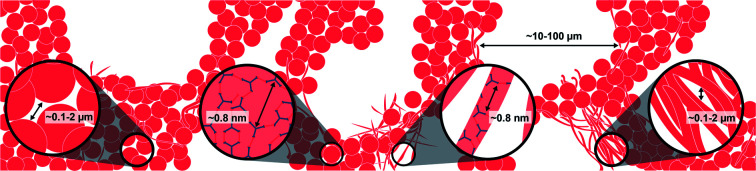

## 1. Introduction

Networks are three-dimensional molecular structures built up by covalently-linked building blocks, where at least one building block has a functionality *f* > 2. Depending on the connectivity and topology of the building blocks and the degree or absence of network interpenetration, different segment lengths and sizes of voids between the segments result. *Networks* are amorphous, *i.e.* do not exhibit long-range order, in contrast to their crystalline counterparts *frameworks*. For instance, amorphous silica (SiO_2_) is an inorganic network composed of the covalently-linked building blocks Si (*f* = 4) and O (*f* = 2), and quartz is one of its framework counterparts. Compared to the corresponding framework, a network architecture often engenders materials properties such as mechanical strength (*vs.*, *e.g.*, fracture along lattice planes in a framework), elasticity, a broader range of pore sizes (*vs.* a narrow pore size distribution in frameworks), and malleability *e.g.* through solvent swelling at increased *T*.^1^ Fully organic networks are typically referred to as polymer networks, and their crystalline counterparts as covalent organic frameworks (COFs). Both polymer networks and COFs are on a molecular scale “infinite” and therefore virtually insoluble in any solvents, and furthermore thermoset, with the exception of systems featuring chemical moieties able to undergo bond exchange reactions within the segments (in the case of polymer networks so-called vitrimers,^2^ or covalent adaptable networks (CANs)).^3^ This limits their analysis to solid-state characterization techniques. Synthetically speaking, there are two major ways of generating a polymer network: (i) linear polymer chains are post-synthetically connected *via* crosslinking and (ii) direct construction of the network from multifunctional monomers (aka multitopic monomers).^2^ One particular type of polymer networks typically generated from multitopic monomers are so-called polyheterocyclics networks. Polyheterocyclics are exclusively composed of aromatic building blocks linked *via* heterocyclic moieties. Polyheterocyclic networks are interesting, because they combine porosity with high thermal and chemical stabilities (imparted through being built up of exclusively strong bonds) with functionality (imparted through the heteroatoms and conjugation). Examples of networks of the polyheterocyclics-type comprise polyimides,^5–7^ triazine-comprising polyimides,^8,9^ polybenzimidazoles,^10–12^ and poly(thiazolo thiazole)s.^13,14^

**Fig. 1:**
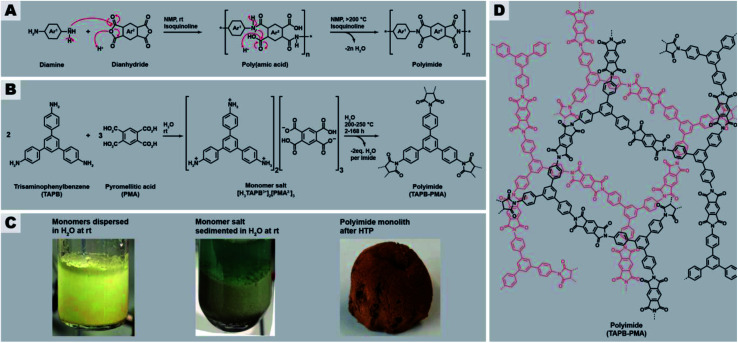
General synthesis route of polyimides and the here studied network PI(TAPB–PMA). (A) Classical synthesis of linear aromatic polyimides through two-step polycondensation *via* poly(amic acids). (B) Reaction equation of the hydrothermal polymerization of PI(TAPB–PMA) from the comonomers TAPB and PMA *via* the monomer salt [H_3_TAPB^3+^]_2_[PMA^2−^]_3_. (C) Aspects of comonomer mixture (left), monomer salt (middle), and PI network (right). (D) Molecular structure of the PI(TAPB–PMA) network.

The major downside of polyheterocyclics is their synthesis, which is harsh and harmful, expensive, and tedious. For example for polyimides, which lie at the center of this contribution, the conventional synthetic route proceeds *via* cyclocondensation reactions between dianhydrides and diamines (in the linear case) employing toxic condensation catalysts like isoquinoline (Fig. 1A). The aromaticity of the employed monomers requires harmful and expensive, high-boiling solvents such as *m*-cresol, *N*-methyl pyrrolidone (NMP), or mesitylene.^15^ The cyclization proceeds *via* two steps (Fig. 1A): first, an intermediary poly(amic acid) (PAA) is formed through nucleophilic attack of one nitrogen of the diamines at one of the electrophilic carbonyl carbon atoms of the anhydrides (leading to amide linking functions), accompanied by the opening of the anhydride ring and formation of the pendant acid functions. Second, the 5-ring imide functions are formed by nucleophilic attack of the amide nitrogens at the pendant CO_2_H functions and subsequent condensation reaction (liberation of H_2_O). Related routes and polymeric intermediates are used for all other types of polyheterocyclics, such as polybenzimidazoles, polybenzoxazoles, or polybenzthiazoles.^4^

Recently, we reported on a synthetic technique that does not require any toxic solvents or catalyst, but – beside the comonomers – solely H_2_O as solvent: hydrothermal polymerization (HTP).^16^ HTP generates highly crystalline PIs and has therefore been described as a geomimetic approach imitating natural hydrothermal (HT) mineral formation processes, specifically HT crystallization of network minerals such as metal oxides (*e.g.* quartz, sapphire), aluminosilicates (*e.g.* zeolites), and silicates (*e.g.* olivine).^17–21^ In striking analogy, both PIs and the latter network minerals form through polycyclocondensations, *i.e.* through the formation of a cyclic moiety connecting the monomeric units, accompanied by the release of a low-molecular weight by-product (H_2_O in the case of both minerals and of PIs). Aside PIs, HTP has recently been shown to be also amenable to the synthesis of polyamide networks, which in the reported example are, however, linked by linear amide functions (and hence no heterocyclic linkages), and based on both aromatic and also alicyclic moieties,^22^ polyazomethines (also not featuring heterocyclic linkages but linear azomethine (aka secondary aldimine) linkages),^23^ and polybenzimidazoles and benzoyl-imidazole polymers (aka BBB – benzimidazolebenzophenanthroline-type semiladder polymers).^24^ HT cyclocondensations can also be used for generating small organic molecules, the technique is then rather termed hydrothermal synthesis (HTS), with examples comprising rylene bisimides,^25^ benzimidazoles,^26^ quinoxalines,^76^ and the fused bisbenzoylimidazole perinone.^27^ HTS/HTP relies on superheated liquid H_2_O as medium, generated through heating H_2_O above its boiling point in a closed vessel (typically an autoclave). High-temperature water features physicochemical properties from which cyclocondensations profit, *e.g.*, decreased polarity and enhanced autoprotolysis leading to simultaneously increased acidity and basicity. Lower polarity enhances the solubility of organic molecules and enhanced autoprotolysis equips H_2_O itself with the ability to act as acidic/basic catalyst. HTS/HTP bears two major advantages: (i) the technique is environmentally friendly, especially in contrast to the conventional routes towards compounds built-up by mainly aromatic and heterocyclic functions, and (ii) the technique is straightforward to implement: the starting compounds are suspended in H_2_O at r.t., and the mixture is sealed in a commercially available steel autoclave and subsequently heated to the desired *T*_r_, *e.g.*, by placing it in an oven for the desired reaction time *t*_r_. After *t*_r_, the autoclave is cooled back to r.t. and hence the medium water becomes polar again, leading to the phase separation of the rather apolar reaction products, which can then be collected by simple filtration (if solid) or decantation (if liquid).^28^ HT conditions have been shown to promote crystallinity in small molecules,^27,29^ linear polyimides,^16,28,30,31^ polyamide networks,^22^ or at least semi-crystallinity in PI/SiO_2_ hybrid materials and linear polybenzimidazoles and BBB.^24,31^ To date, monomers used in HTP were mainly difunctional aromatic monomers, *i.e.*, aromatic dianhydrides,^16,28,31^ aromatic diamines,^16,23,28^ and aromatic dialdehydes.^23^ Some reports employ tetrafunctional aromatic monomers, such as tetraamines,^24^ or in the case of PIs the free tetracarboxylic acids instead of the corresponding dianhydrides.^31^ In these cases however, the 1,2-difunctionality is in fact required to generate the heterocyclic linkages and thus does not lead to networks but linear polyheterocyclics. Only one publication has targeted the preparation of amorphous polymer networks by HTP, specifically a poly(amide imide) network, using mellitic (benzenehexacarboxylic acid; here each pair of adjacent CO_2_H groups is required per intended imide linkage) in combination with different aromatic diamines. However, the reported product is not a fully condensed network or framework, but rather consists of oligomers that can only be converted to a network-type higher molecular weight species by thermal annealing through solid-state postcondenzation at 340 °C.^32^

With this contribution, we have set out to, for the first time, hydrothermally prepare highly condensed aromatic PI networks. We chose the comonomers 1,3,5-tris(4-aminophenyl)benzene (TAPB) and pyromellitic acid (PMA) to synthesize the network PI(TAPB–PMA), see Fig. 1B. HTP of PI(TAPB–PMA) occurs, *via* the isolable multitopic monomer salt [H_3_TAPB^3+^]_2_[PMA^2−^]_3_ (Fig. 1B and D), which forms by acid–base reaction between the monomers at low *T*, *i.e.* at the beginning of heating of the reaction mixture. While HTP to PIs has been shown to involve monomer salts (MS) on all occasions reported to date,^16,24,27,28,30,31,33–35^ a multitopic MS has to date not been reported, including in the field of solid-state polymerizations of MS.^36^ For their insolubility, all PI(TAPB–PMA) networks where characterized *via* several solid-state techniques, including Fourier-transform infrared attenuated-total-reflection spectroscopy (FT-IR-ATR), X-ray diffractometry (XRD), thermogravimetric analysis (TGA), gas sorption (low-pressure CO_2_ physisorption), Hg porosimetry, and scanning electron microscopy (SEM). We show that and discuss why all PI networks are amorphous, despite both TAPB and PMA being rigid comonomers and HTP's propensity to generate crystalline products. Furthermore, through screening various reaction parameters and SEM analysis of all products, we find a wealth of distinct micromorphologies that occur in combination in the samples. We develop a hypothesis of the formation and underpinnings of these morphologies based on expert interpretation of hundreds of SEM images and cross-referencing with spectroscopic and diffraction data. Furthermore, we have devised a computational analysis pipeline that analyses these complex high-content images in an automated fashion. We believe that this approach is of great interest for the materials science/chemistry community, in light of the large analytical datasets generated nowadays, for instance by accelerated materials discovery through automation.^37,38^ Finally, we show that the HTP of PI(TAPB–PMA) can be upscaled, and that bulk materials can be generated through solvent-free warm-pressing.

## 2. Results and discussion

The general procedure for all HTP experiments performed in this study employs the commercially available monomers TAPB and PMA (Fig. 1B, ESI for further details†). TAPB and PMA were first suspended in H_2_O at r.t., transferred to an autoclave which was subsequently sealed, immediately heated to the reaction temperature *T*_r_ (200 or 250 °C), and kept there for the reaction time *t*_r_. Instead of first preparing the MS [H_3_TAPB^3+^]_2_[PMA^2−^]_3_, which is possible (Fig. 1B and D and ESI†), we started directly from the comonomers, as the MS inevitably forms at low *T* during heating of the reaction mixture to the desired *T*_r_. After the desired *t*_r_, the autoclave is allowed to slowly cool down to r.t., and the product is isolated by filtration, washed several times with subsequently cold aq. dest., ethanol, and acetone, dried *in vacuo* at 80 °C overnight, and analyzed by FT-IR-ATR, TGA, and XRD.

### 2.1 Benchmark system: PI(TAPB–PMA) synthesized at *t*_r_ = 24 h and *T*_r_ = 200 °C

**Fig. 2:**
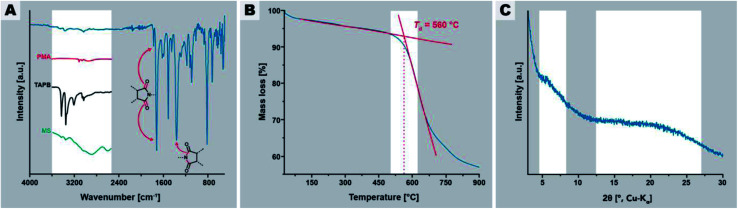
Characterization of PI(TAPB–PMA) synthesized at 200 °C for 24 h. (A) FT-IR-ATR spectrum of PI(TAPB–PMA) with characteristic imide modes highlighted by arrows; insets show the H-bonding region 4000–2250 cm^−1^ of spectra of PMA (red curve), (ii) TAPB (black curve), and the MS (green curve). (B) TGA of PI(TAPB–PMA) under N_2_ flow. (C) Powder XRD pattern of PI(TAPB–PMA) with the two halos highlighted.

In a first experiment, we employed *t*_r_ = 24 h and *T*_r_ = 200 °C, which are standard HTP conditions.^24,30^Fig. 2 summarizes the characterization of this initial PI(TAPB–PMA). FT-IR-ATR spectroscopy (Fig. 2A) indicates high degrees of condensation of PI(TAPB–PMA). Absorption modes at ∼1775 and ∼1720 cm^−1^ arising from imide C=O stretching vibrations, as well as the imide C–N stretching mode at ∼1365 cm^−1^ are clearly present and confirm the formation of the imide moiety. Additionally, the characteristic amine (∼3400–3300 cm^−1^), carboxylic acid C=O (∼1850–1760 cm^−1^), and MS modes (aryl-NH_3_^+^ at 2850 and 2580 cm^−1^, and aryl-CO_2_^−^ at 1715 cm^−1^) are missing, which strongly points at both the absence of unreacted monomers and MS (*cf.* insets in Fig. 2A), and suggests substantial degrees of condensation (*i.e.* to a point where end groups are not detectable anymore). Moreover, TGA of PI(TAPB–PMA), shows a high decomposition temperature *T*_d_ of ∼550 °C and a small weight loss of around 4 wt% between r.t. and 200 °C and of in total ∼7 wt% until 560 °C (Fig. 2B). We attribute this to physisorbed H_2_O (which we expect to be mainly a reminiscence from the synthesis in water). While this value seems at first sight large for physisorbed H_2_O, both the amount and the temperatures of release significantly above 100 °C are in alignment with micro- and mesoporous hosts. Furthermore, TGA does not show a condensation step as one would expect for oligomers/monomers. Yet, full condensation cannot be claimed, and some extent of end-groups, albeit likely very small, cannot be excluded. Powder XRD (PXRD) measurements reveal that the PI(TAPB–PMA) network is amorphous with the diffractogram showing two broad features (Fig. 2C, gray boxes) and no sharp reflections.

**Fig. 3:**
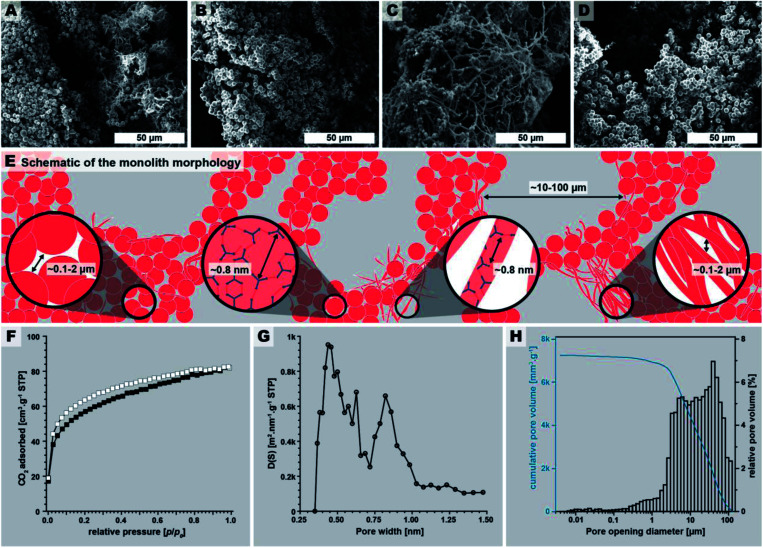
PI(TAPB–PMA) monolith micromorphology, structure, and porosity. (A–D): SEM images of the monolith. (E) Schematic of the monolith micromorphology. (F) Low pressure CO_2_ physisorption isotherm measured at 195 K (adsorption: white squares; desorption: black squares). (G) Pore size distribution (PSD) analysis shows a majority of ultramicropores (<1 nm). (H) Pore opening diameter distribution obtained by Hg intrusion porosimetry (max. intrusion pressure 400 MPa).

The macroscopic appearance of PI(TAPB–PMA) fabricated by this initial synthesis was intriguing: a sponge-like polymer monolith was obtained (Fig. 1E). SEM analysis (Fig. 3A–D) shows that the monoliths are composed of coexisting spherical near-monodisperse particles (∼3–5 μm in diameter) and roundish branched fibers (∼1 μm in diameter, ∼20 μm in length). The particles and fibers are closely packed, forming the monolith cell walls that are ∼20–100 μm thick. The monolith cells exhibit void spaces of ∼10–100 μm between the cell walls (Fig. 3E).

The macroscopic appearance as monolith and the intriguing micromorphology from SEM prompted us to investigate PI(TAPB–PMA)'s porosity. Therefore, gas sorption experiments were carried out using CO_2_ as analyzing gas. The permanent porosity was evaluated by low-pressure carbon dioxide (CO_2_) sorption studies at 195 K and 273 K of the at 120 °C for 24 h evacuated PI(TAPB–PMA) sample (Fig. 3F and G). A typical type-I isotherm was observed (Fig. 3F) and is indicative of the sample's microporosity.^39^ The Brunauer–Emmett–Teller surface area (SA_BET_) calculated from the CO_2_ adsorption points at 195 K was found to be 207 m^2^ g^−1^. The pore size distribution (PSD) analysis derived by fitting a Monte Carlo model for carbon adsorbents to the collected isotherm at 273 K indicated the predominant presence of ultra-micropores with pore diameters below 0.8 nm (Fig. 3G). Note that we also synthesized PI(TAPB–PMA) at longer reaction times (see Section 2.2), and also performed gas sorption analysis of selected products. Specifically, gas sorption experiments of PI(TAPB–PMA) samples prepared at *T*_r_ = 200 °C and *t*_r_ = 48 h, 60 h, 72 h and 168 h, respectively, were analyzed (ESI†), and all revealed a typical type-I isotherm. The samples' porosity was furthermore investigated by Hg porosimetry, revealing that the majority of porosity within the material can be assigned to pores in the size range of 1–100 μm (Fig. 3H), corresponding to the large interstitial space between the monolith cell walls (∼20–100 μm from SEM). Fig. 3H also evinces pores in the size range 0.1–2 μm, which we attribute to the porosity within the monolith cell walls, *i.e.*, interstitial voids between spherical microparticles of PI(TAPB–PMA). Finally, small relative amounts of mesoporosity (2–50 nm) can be identified from Fig. 3H, which we believe to correspond to interstitial voids between microfibers, and between microparticles and microfibers. Hence, intriguingly and prompted by both its molecular architecture and micromorphology, PI(TAPB–PMA) possesses three types of porosity. In accordance with SEM images (Fig. 3A–C), we believe the porosity to be hierarchical. From the total Hg uptake (306.82 mm^3^/0.0423 g), a very high specific pore volume of 7250 mm^3^ g^−1^ was calculated, which is in fact one order of magnitude larger than the specific pore volumes of PI(TAPB–PMA) synthesized by classical polycondensations using organic solvents and catalysts (670 and 310 mm^3^ g^−1^ (ref. ^40^ and ^41^); ESI Table S2†). Yet, SA_BET_ of PI(TAPB–PMA) from HTP is smaller than from conventional synthesis (586, 570, and 1297 m^2^ g^−1^ (ref. ^40–42^) Table S2†). We suspect that the higher apolarity of solvents used in conventional synthesis produces solvent swelling of these networks during the synthesis, resulting in higher porosity in the microporous range. Such effects have been previously reported for linear polyimides.^43^ The PSD (Fig. 3G) reflects significant amounts of ultramicropores, which is consistent with the networks' segments being closer to each other when synthesized by HTP *vs.* classically. This supports our hypothesis that PI(TAPB–PMA) is less swollen by solvent molecules from HTP than when synthesized in organic solvents. A further contrast to the conventionally synthesized analoga is that they exclusively feature micropores, while PI(TAPB–PMA) from HTP instead exhibits ultramicropores, and additionally mesopores and macropores, imparted through the unique morphology obtained by HTP.

So far, we have demonstrated through the initial benchmark HTP of PI (TAPB–PMA), that an amorphous, highly condensed network monolith exhibiting hierarchical porosity and the – to date – largest cumulative specific pore volume reported for this PI can be obtained in nothing but hot water from a stoichiometric mixture of the comonomers at *t*_r_ = 24 h and *T*_r_ = 200 °C. From this starting point, we were curious to determine the effects of employing different reaction parameters, which we discuss in the following.

### 2.2 Screening of the reaction parameters of the HTP of PI(TAPB–PMA) and their effect on chemical nature and long-range order within the samples

To determine if the properties of PI(TAPB–PMA) could be tuned through the reaction parameters, we screened various reaction conditions. Specifically, we probed *T*_r_, *t*_r_, and the use of the additive acetic acid (HOAc), for the following reasons: (i) *T*_r_ was varied as previous organic HTS have shown that in some cases a minimum *T*_r_ is necessary,^25^ and that increasing *T*_r_ can speed up HT cyclocondensations.^27^ (ii) *t*_r_ was varied since it can affect condensation and crystallinity.^31^ (iii) We also chose to investigate the effects of the addition of HOAc, as its use as morphology modulator in HTP has been reported,^31^ and was thought to potentially also be able to alter the degrees of condensation and crystallinity. The washed and isolated products of all experiments (also summarized in Fig. 5) were analyzed by TGA, FT-IR-ATR, XRD, and SEM. When comparing the characterization results of all products, the vast majority of samples were found to be chemically identical, *i.e.*, as concluded from FT-IR-ATR analysis (*cf.* ESI†): a high degree of condensation and full conversion of comonomers is indicated by the presence of imide modes and the absence of modes related to NH_2_ and CO_2_H (indicative for monomers and end-groups). A small number of samples depicted differences in FT-IR-ATR spectra, notably when synthesized at 250 °C at long reaction times (*t*_r_ > 60 h). Specifically, no matter if with or without HOAc, in samples synthesized at 250 °C, at *t*_r_ = 60 h, the imide modes' (1775, 1720, and 1365 cm^−1^) intensity is strongly decreased, and at *t*_r_ = 72 h and 168 h (7 d) they are barely visible anymore (ESI†). While this could stem from a decomposition of the samples, it is striking that the spectra still feature overall sharp peaks and that the majority of peaks, *e.g.*, in the fingerprint region, seem unaffected. At the same time, new peaks appear, notably (i) 7 new peaks in the H-bonding region (3625, 3325, 3200, 3020, 3820, and 2690 cm^−1^), (ii) a relatively weak but sharp mode appears ∼1700 cm^−1^, (iii) a strong broad mode at ∼1100 cm^−1^ that increases from *t*_r_ = 60 to *t*_r_ = 168 h, and (iv) a weak and broad mode at ∼900 cm^−1^ that is only present at *t*_r_ = 60 h and 72 h, but absent at 168 h. The strong broad mode nicely fits—both in shape and wavenumber—to the asymmetric siloxane (Si–O–Si) vibration, reported in the literature to occur at 1180 cm^−1^.^44^ The weak mode at ∼900 cm^−1^ (only found at *t*_r_ = 60 and 72 h) as well as the modes in the H-bonding region both nicely fit to silanol (Si–OH) groups (Si–OH bonding is reported in the literature as to occur at 3500–3000 cm^−1^).^44^ Therefrom, we infer that at long *t*_r_ > 60 h at sufficiently high *T*_r_ of 250 °C, small parts of the glass liners in which the reactions are performed dissolve and form SiO_2_. From the absence of imide modes in the IR spectra that otherwise do not point at decomposition, we infer that the cyclic imides are opened up. Opening of the imide moieties should happen trough C–N bond hydrolysis of R–C(O)–N–R′, hence generating RCO_2_^−^/RCO_2_H and RNH_2_/RNH_3_^+^ moieties. Yet, there are no modes characteristic for MS or monomers, which prompted us to wonder if the imide moieties had not only opened up, but in fact reacted with Si–OH functions towards a covalently linked hybrid material. We hypothesize that the covalent linking to occur by condensation of amic acid intermediates R–C(O)N–CO_2_H (as in PAAs, *cf.*Fig. 1A) with Si–OH, *i.e.*, through silyl ester linking functions. In fact, the new mode at ∼1700 cm^−1^ fits well to silyl ester C=O modes^45^ and to amide C=O modes. Furthermore, aromatic amides' N–H stretching modes also fit to the new peaks observed in the H-bonding region.^46,47^ Taken together, we believe from FT-IR-ATR that small parts of the glass liners that were exposed to HT conditions at 250 °C for a long time (≥60 h), dissolve as silicic species, and, most intriguingly, in fact react with PI(TAPB–PMA) to form a covalently-linked hybrid material.

**Fig. 4:**
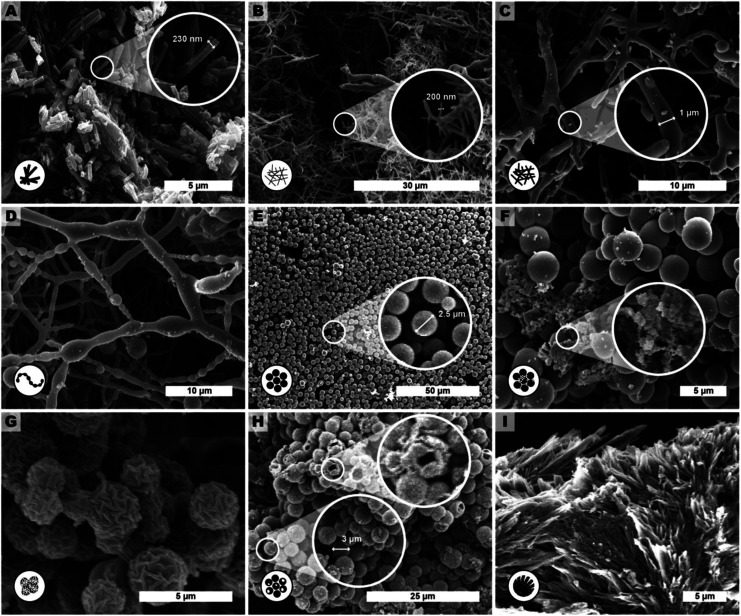
Representative SEM images of products of extended reaction parameter screening of HTP of PI(TAPB–PMA). The described morphologies are: (A) monomer salt crystallites (*t*_r_ = 24 h, *T*_r_ = r.t.), (B) fibers (*t*_r_ = 2 h, *T*_r_ = 200 °C.), (C) thicker fibers (*t*_r_ = 6 h, *T*_r_ = 200 °C), D pearl-chain fibers (*t*_r_ = 12 h, *T*_r_ = 200 °C), (E) near-monodisperse particles (*t*_r_ = 48 h, *T*_r_ = 250 °C), (F) seed particles on top of microspheres (*t*_r_ = 72 h, *T*_r_ = 200 °C), (G) wrinkle-textured microspheres (*t*_r_ = 60 h, *T*_r_ = 250 °C), (H) hollow spheres (*t*_r_ = 72 h, *T*_r_ = 250 °C, with HOAc) and (I) angular particles (*t*_r_ = 72 h, *T*_r_ = 250 °C).

**Fig. 5:**
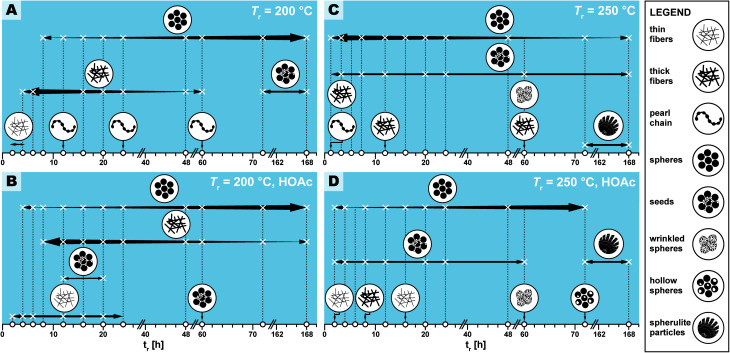
Appearance and relative abundance of limit micromorphologies in SEM images of PI(TAPB–PMA) reaction condition screening. Legend of limit morphologies is shown on the right. (A–C) Appearance (X) and relative abundance (thickness of arrows) deduced from manual inspection of SEM images. White circles on the *x*-axis indicate at which specific time HTP experiments were performed. Each panel represents a pair of *T*_r_ and HOAc presence/absence, specifically (200 °C|no HOAc) (A), (200 °C|with HOAc) (B), (250 °C|no HOAc) (C), (250 °C|with HOAc) (D).

The XRD patterns of all samples exhibit the two amorphous features discussed earlier (Section 2.1, Fig. 2C). Yet, just as for FT-IR-ATR results, we also find the XRD patterns of a few samples to differ from all others, notably for samples obtained after long reaction times at *T*_r_ = 250 °C. These samples additionally show 3–5 weak but relatively sharp reflections (3 when HOAc is absent, the same three plus two more when HOAc is present, see ESI†). Specifically, we find the following additional reflections: (i) 16.1° (2*θ*, Cu-K_α_), corresponding to *d*_*hkl*_ = 5.5 Å (*n* = 1); (ii) 17.5° (2*θ*, Cu-K_α_), corresponding to *d*_*hkl*_ = 5 Å (*n* = 1); (iii) 18.6° (2*θ*, Cu-K_α_), corresponding to *d*_*hkl*_ = 4.8 Å (*n* = 1); (iv) 25.2° (2*θ*, Cu-K_α_), corresponding to *d*_*hkl*_ = 3.5 Å (*n* = 1); (v) 27.9° (2*θ*, Cu-K_α_), corresponding to *d*_*hkl*_ = 3.2 Å (*n* = 1). All five are present in the sample with HOAc at *T*_r_ = 250 °C and *t*_r_ = 7 d, and only (ii), (iv), and (v) are present with HOAc at *T*_r_ = 250 °C and *t*_r_ = 2 h, and without HOAc at *T*_r_ = 250 °C and *t*_r_ = 48 h, 60 h, 72 h, and 7 d. We initially suspected that these reflections would correspond to the SiO_2_ polymorph quartz. Yet an overlay of the diffractograms of our samples synthesized at *T*_r_ = 250 °C for long *t*_r_, shown in the ESI,† with the diffractograms of, for completeness, all crystalline SiO_2_ polymorphs found in the ICDD database (*i.e.* α-quartz: PDF 01-070-3317; β-quartz: PDF 01-089-8951; α-tridymite: PDF 01-073-6613; β-tridymite: PDF 04-005-4647; α-cristobalite: PDF 04-018-0236; β-cristobalite: PDF 01-089-3435; keatite: PDF 01-077-3514; moganite: PDF 00-052-1425; coesite: PDF 01-077-1725; seifertite: PDF 04-023-2204; melanophlogite: PDF 04-015-4104; and stishovite: PDF 00-045-1374) shows no matching reflections. Based on not matching to any SiO_2_ crystal structure in combination with FT-IR-ATR pointing at covalent linking through Si–O–R, Si–N–R, and SiO–(C=O)R, we suspect that the 3–5 new reflections found correspond to a somewhat ordered hybrid material. Interestingly, in a recent work on the HTS of covalently linked SiO_2_-PI hybrids (hybrids prepared on purpose through employing the linker compound 3-(aminopropyl)triethoxysilane), we found similar reflections.^31^

In summary from all FT-IR-ATR and XRD results, we conclude that neither the conversion of monomers nor the degree of condensation of the PI network, including its order, are significantly affected by *t*_r_ and the presence of HOAc at *T*_r_ = 200 °C. At *T*_r_ = 250 °C, at short to medium *t*_r_ (until 24 h) both with and without HOAc, conversion and crystallinity are also not observably affected. Hence, one can conclude that HTP of PI(TAPB–PMA) is a fairly robust process under these conditions. Yet, at 250 °C and long *t*_r_ (≥48 h), silica species seem to dissolve from the glass liner and in fact appear to react with PI(TAPB–PMA) towards a somewhat ordered hybrid material.

Macromorphologically, the samples differ relatively strongly. The observed macromorphologies range from elastic, sponge-like monoliths to brittle monoliths and powders, and correlate with the HTP conditions. At short *t*_r_ (<48 h) elastic, spongy monoliths are obtained. Beyond *t*_r_ = 48 h, we first obtain brittle monoliths, and subsequently mixtures of macroscopic pieces of different sizes with fine powders. Notably, at *t*_r_ = 72 h and 168 h, such mixtures are obtained exclusively. The most intact monoliths are also the most elastic ones, and are obtained after 12–24 h at 200 °C. The monoliths are generally more intact and elastic in the absence of HOAc.

Finally, the samples were analyzed by TGA, as decomposition temperatures (*T*_d_) and char yields might also allow for drawing conclusions with respect to chemical composition. In terms of thermal stability, we find *T*_d_ ∼ 560 ± 35 °C (lowest *T*_d_ determined: 525 °C, highest *T*_d_ determined: 595 °C, Table S1†) for all *t*_r_ at *T*_r_ = 200 °C, and until *t*_r_ = 60 h at *T*_r_ = 250 °C. Interestingly, the thermal stability of hydrothermally generated PI(TAPB–PMA) thus exceeds the reported classically prepared analoga by up to 70 °C using the tangents method (*T*_d_ = 530 and 590 °C of classical analoga *vs.* 602 °C for PI(TAPB–PMA) by HTP).^41,42^ As becomes clear from plotting all determined *T*_d_ against *t*_r_ (ESI†), there is no clear trend, *i.e.*, these variations of ±35 °C do not correlate with *t*_r_ for *T*_r_ = 200 °C, and also do not correlate with *t*_r_ until *t*_r_ = 60 h. The variations point towards slight differences in the extent of condensation (*i.e.* “size” of the networks) that are undetected within the limits of FT-IR-ATR, but still have consequences for *T*_d_. For *t*_r_ > 60 h at 250 °C, the *T*_d_ values decrease drastically (∼250 °C), which points at a disintegration of the networks to oligomers or monomers. The char yields (remaining sample mass in wt% at the end of the TGA experiment, *i.e.*, 900 °C) vary between 42 and 59 wt%, except for samples obtained at 250 °C for long *t*_r_, which show considerably higher values (*e.g.* 250 °C, 7 d, without HOAC: 77 wt%, or 250 °C, 72 h, with HOAc: 75 wt%, see ESI†). These significant increases in char yields point at some mineral content. In agreement with ATR-FT-IR and PXRD data, we hypothesize that the reason is SiO_2_ components in samples obtained at 250 °C for long *t*_r_. The micromorphologies observed by SEM are fairly complex, and therefore discussed in detail in the following section.

### 2.3 The micromorphological evolution of PI(TAPB–PMA) as a function of the HTP the reaction parameters

**Fig. 6:**
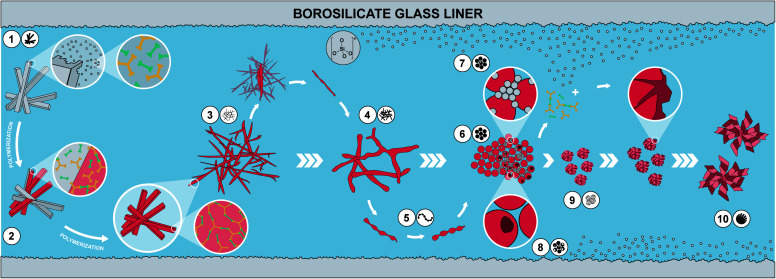
Proposed hypothesis of the morphological evolution leading to monodisperse PI particles. (1) Dissolution of a small fraction of MS crystallites; (2) polymerization of dissolved MS and deposition on yet undissolved MS crystallites leading to (3) thin fibers; (4) softening of thin fibers and merging into thicker fibers; (5) further softening and separation *via* pearl chain-like structures into (6) spheres, (which assemble into particle networks building up the monoliths (not shown)). At 250 °C, (7) dissolution of SiO_2_ from glass liners and reprecipitation as nanometric SiO_2_ particles; dissolved SiO_2_ species react with PI networks forming hybrid materials on the surface of existing PI spheres; (8) monomers leaching out from PI particles creates hollow spheres. SiO_2_/PI hybrids' growth generates (9) wrinkled sphere and subsequently (10) spherulite morphologies.

We observed several distinct micromorphological features that co-exist to different degrees throughout the samples. Specifically, we found eight distinct morphologies. The distinct morphologies, plus that of the MS, are summarized in Fig. 4: (i) cuboid monomer salt crystals, ∼1–5 μm in length times ∼100–200 nm in width (Fig. 4A); (ii) thin roundish needle-like fibers, ∼100–200 nm in width and several μm in length (Fig. 4B); (iii) thicker coalesced roundish fibers that feature branching, ∼1 μm in thickness and several μm in length (Fig. 4C); (iv) pearl-chain fibers; several μm in length and featuring globular slubs of ∼2 μm in diameter (Fig. 4D); (v) near-monodisperse spherical particles, ∼2–5 μm in diameter (Fig. 4E), (vi) roundish nanoparticles, ≤100 nm in diameter, henceforth referred to as “seeds” (Fig. 4F); (vii) wrinkle-textured microspheres, ∼2–5 μm in diameter (Fig. 4G), hollow spheres, ∼2–5 μm in diameter (Fig. 4H), and (viii) spherulitic structures, up to ∼50 μm in length and up to ∼180° in circle segment angle, composed of twisted fibers of ∼5–10 μm in diameter and ∼50 μm in length (Fig. 4I). From expert analysis of the SEM micrographs of all investigated samples (439 micrographs in total) produced in the screening, we devised the morphological evolution of PI(TAPB–PMA), as a function of *t*_r_, *T*_r_, and the presence of HOAc, summarized in Fig. 5. In Fig. 5, every panel stands for a pair of *T*_r_ and with/without HOAc Fig. 5A (200 °C|no HOAc), Fig. 5B (200 °C|with HOAc), Fig. 5C (250 °C|no HOAc), Fig. 5D (250 °C| with HOAc). *t*_r_ is plotted on the *x*-axis, and white circles on the *x*-axis indicate at which specific *t*_r_ HTP experiments were carried out. Schematics for each of the previously described eight distinct micromorphologies are used to illustrate in which sample a morphology was found. Black two-sided arrows indicate the timespan over which the morphology was found, with the relative size of the arrow tips illustrating the relative abundance of the morphology. Through this display, the following steps of the morphology evolution during the HTP of PI(TAPB–PMA) become clear:

(i) Fig. 5A (*T*_r_ = 200 °C|no HOAc): (1) thin fibers are found only briefly, at short *t*_r_ of 2–4 h. For their matching dimensions, we believe that the thin fibers result from MS crystals (SEM in Fig. 4A) serving as solid templates on which initially formed PI(TAPB–PMA) is deposited (*cf.* schematic of the micromorphological evolution, Fig. 6). (2) The thicker coalesced fibers are also found over a medium *t*_r_ (4–60 h), compared to corresponding HTP (same *T*_r_) with HOAc (there they are found at 8–168 h). The relative amounts of both thick and thin fibers decrease with increasing *t*_r_. (3) Spherical microparticles start to appear at *t*_r_ = 8 h and persist until the longest *t*_r_ of 168 h. Their relative amount increases with *t*_r_. We think that the degree of condensation increases from the thin fibers to the thick fiber to the sphere morphologies (*cf.*Fig. 6). (4) Pearl-chain morphologies are only found in three samples (*t*_r_ = 12, 24, 72 h) and neither in large amounts nor repeatedly (*i.e.* in several consecutive samples). We think that they are somewhat a snapshot of the thicker fibers softening and separating into the spherical particles. Therefore, they would be short-lived, and we hence do not find them in many samples. (5) We additionally find “seed” nanoparticles at long *t*_r_ = 72 and 168 h.

(ii) Fig. 5B (*T*_r_ = 200 °C|with HOAc): the morphologies found and the overall trends with respect to their appearance and abundance are similar to the samples prepared at the same *T*_r_ but in the absence of HOAc. Yet, several morphologies are present over slightly different timescales. (1) The thin fiber morphologies appear earlier and persist for longer times, *i.e.*, 2 h ≤ *t*_r_ ≤ 20 h (compared to 2 h ≤ *t*_r_ ≤ 4 h in the absence of HOAc). (2) The spherical particles also appear earlier and persist for longer times, *i.e.*, 4 h ≤ *t*_r_ ≤ 168 h (compared to 8 h ≤ *t*_r_ ≤ 4 h in the absence of HOAc). As for HTP in the presence of HOAc, their abundance increases with increasing *t*_r_. The observation that thin fiber morphologies appear earlier in the presence than in the absence of HOAc points at HOAc initially speeding up the MS dissolution and initial reaction to oligomers. However, the observation that these morphologies persist for longer *t*_r_ points at HOAc slowing down the subsequent polymerization. (3) In contrast, the thick fibers appear later and persist for longer, *i.e.*, 8 h ≤ *t*_r_ ≤ 20 h (compared to 2 h ≤ *t*_r_ ≤ 4 h without HOAc). Again, this points at HOAc slightly slowing down the polymerization at 200 °C. The thick fibers' abundance also increases with increasing *t*_r_, as for (*T*_r_ = 200 °C|no HOAc). (4) Pearl-chain morphologies are not found at all. As previously discussed, we believe the pearl-chain morphologies to be short-lived, and hence not finding them in SEM images is in our opinion merely coincidental. (5) Seed particles are found significantly earlier, *i.e.* at *t*_r_ = 12, 20, and 60 h. We believe that these small particles (≤100 nm in diameter) correspond to SiO_2_. We do not find indications of SiO_2_ in FT-IR-ATR or PXRD for these reaction times, and in SEM we only find them in very small relative amounts. Hence, the absence of indication for SiO_2_ at the corresponding *t*_r_, in FT-IR-ATR and PXRD is likely explained through both techniques sensitivity (in PXRD for amorphous structures). That these SiO_2_ nanoparticle morphologies appear earlier in the presence of HOAc is consistent with the literature: geochemical studies have shown that, *e.g.*, quartz can dissolve in H_2_O in small quantities,^48^ and that such dissolution is up to one order of magnitude faster in the presence of (organic) acids.^49–51^

(iii) Fig. 5C (*T*_r_ = 250 °C|no HOAc): (1) no thin fibers are found at any *t*_r_, pointing at HTP being much more rapid at 250 °C, which is expected. (2) Thick fibers are found in 3 instances and in low amounts, *i.e.*, at *t*_r_ = 2, 12, and 60 h. (3) Spherical particles are present through the entire *t*_r_ span studied, and their abundance decreases with increasing *t*_r_. Again, this points at HTP being much faster at the higher *t*_r_ of 250 °C. (4) Seeds are also present through the entire time span studied, at low but constant abundance. This is consistent with the literature on SiO_2_ dissolution in H_2_O and its re-precipitation being faster with increased *T*_r_.^48^ (5) Pearl-chain morphologies are only found in one sample, at the lowest tested *t*_r_ of 2 h. (6) Additionally, we find a “wrinkle-covered sphere” morphology at *t*_r_ = 60 h. We think that this intriguing morphology is also corresponding to SiO_2_/SiO_2_-PI hybrids. In fact, such morphologies have been described by Oehler in a geochemical study on the hydrothermal crystallization of silica gels.^52^ Oehler describes them as “honeycomb-textured microspheres”, likely for the particles' surface textures resembling honeycomb-weathering in sandstone, and explains these morphologies as the result of a complex crystallization process of subsequent steps with different growth rates. (7) Finally, we find spherulitic objects at *t*_r_ = 72 and 168 h. These are composed of twisted fibers ∼5–10 μm in diameter and ∼50 μm in length. We think that these fibers are composed of the ordered SiO_2_/PI(TAPB–PMA) hybrid of which we have hypothesized its existence in Section 2.2 from FT-IR-ATR and PXRD data. Spherulitic structures are the most common crystal habit across all crystalline substances, and numerous naturally solution-grown minerals form spherulites.^53^ In fact, chalcedony, *i.e.* fibrous quartz, is the most well-known spherulite-forming mineral.^53^ Fibrous quartz is often consisting of twisted fibers, and is typically generated hydrothermally.^54^ Hence, finding spherulites of twisted fibers at long *t*_r_ at the higher *T*_r_ of 250 °C is in agreement with our hypothesis of silica-rich SiO_2_/PI(TAPB–PMA) hybrids.

(iv) Fig. 5D (*T*_r_ = 250 °C|with HOAc): the observed micromorphologies are similar to those found at 250 °C without HOAc, but are found over different time spans. Morphologies related to PI(TAPB–PMA) are found later than without HOAc at 250 °C, suggesting, once more, that the presence of HOAC slows down HTP. At the same time, morphologies related to SiO_2_ or SiO_2_/PI(TAPB–PMA) seem to be favorably formed in the presence of HOAc. (1) Thin fibers are found at *t*_r_ = 2 h and 16 h. Since these are related to the early stages of HTP, *i.e.*, an initial polymer deposition on external MS nuclei, their existence points at HOAc slowing down the HTP (compared to 250 °C no HOAC). (2) Thick fibers are only found in one sample, at *t*_r_ = 8 h. (3) Spherical particles are found at 2 h ≤ *t*_r_ ≤ 72 h, and interestingly, their amount increases with increasing *t*_r_. Again, this further substantiates the hypothesis that HOAc at 250 °C slows down HTP. Without HOAc at 250 °C, spherical particles are present from the beginning and their abundance decreases with *t*_r_. (4) We do not find pearl-chain morphologies. (5) Seed particles are present at 2 h ≤ *t*_r_ ≤ 60 h, and their abundance stays constant throughout that time span. (6) Wrinkle-covered spheres are found at *t*_r_ = 60 h, and (7) fibrous spherulites are found at the highest *t*_r_ of 72 h and 168 h. Finally, (8) we observe hollow sphere morphologies. Hollow morphologies are often generally pointing at the dissolution of an object's interior rather than its exterior, because the outer surface is already covered with another material acting as barrier for dissolution. Such morphologies are known for various solution-synthesized materials, *e.g.* CaCO_3_.^55^

We have summarized the overall morphological transformation in Fig. 6. We hypothesize that the following morphological evolution takes place: first, at low *t*_r_, the MS [H_3_TAPB^3+^]_2_[PMA^2−^]_3_ forms as angular needle-like crystallites. Second, some MS dissolves in high-temperature water (HTW) and starts polymerizing to PI(TAPB–PMA). Third, these initial PI(TAPB–PMA) fragments (suspected to be oligomeric) deposit onto not yet dissolved MS crystals and hence, we find thin needles that roughly match the dimensions of MS crystals. Fourth, thick fibers form by both further polymerization and softening and merging of the thin fibers. Fifth, the thick fibers soften and, driven by minimizing their interface with H_2_O, start splitting into more roundish morphologies. The pearl-chain morphologies are a snapshot of this splitting. Sixth, the morphologies resulting from the splitting of thick fibers are near-monodisperse spherical PI(TAPB–PMA) particles. Seventh, at prolonged reaction times at 200 °C (*t*_r_ > 60 h) and at 250 °C right from the beginning, silica (likely in the form of silicic acids) dissolves from the glass liners and starts reprecipitating as nanoparticles, which we refer to as “seeds”. Eighth, SiO_2_-rich hybrids form through reaction of SiO_2_ seeds and further silicic acid species with the present PI(TAPB–PMA). The morphologies related to hybrid formation include wrinkle-covered spheres, fibrous spherulites, and hollow spheres (which we speculate to arise from PI leaching out/depolymerizing before subsequently forming hybrids).

To ascertain our hypothesis that several morphologies obtained at high *T*_r_ and long *t*_r_ were related to SiO_2_ dissolution and reprecipitation in the form of a hybrid material, we performed EDX analyses (see ESI†). EDX results reveal that not only Si but also Al and B are found in the samples. These elements are all part of the borosilicate glass liners used. We conclude that not only SiO_2_ but also other species leach out of the used glass liners. We believe that what is primarily formed is a hybrid of SiO_2_ and PI(TAPB–PMA), especially considering that all aromatic modes of PI(TAPB–PMA) in ATR-FT-IR are still present and that we also find amide modes. At this point, we cannot tell if Al and B are also incorporated in what we suspect to be hybrids, or form boro-/aluminosilicate species that coexist with the presumed PI(TAPB–PMA)/SiO_2_ hybrid.

Finally, we performed solid-state polymerization (SSP) experiments of [H_3_TAPB^3+^]_2_[PMA^2−^]_3_ to check if any of the morphologies in the HTP of PI(TAPB–PMA) correspond. It is conceivable, and has been previously reported, that monomer salts can undergo SSP under HT conditions when they are not soluble, *i.e.*, SSP of MS particles occurs in dispersion in high-temperature water. However, SEM analysis clearly evinces that PI(TAPB–PMA) from SSP appears as shape copies of the MS crystals (see ESI†). This is in accordance with previous reports on the morphology of PIs from SSP of MS.^34,56^ Hence, we conclude that SSP does not play a role in the here observed morphologies.

### 2.4 Computational image analysis

**Fig. 7:**
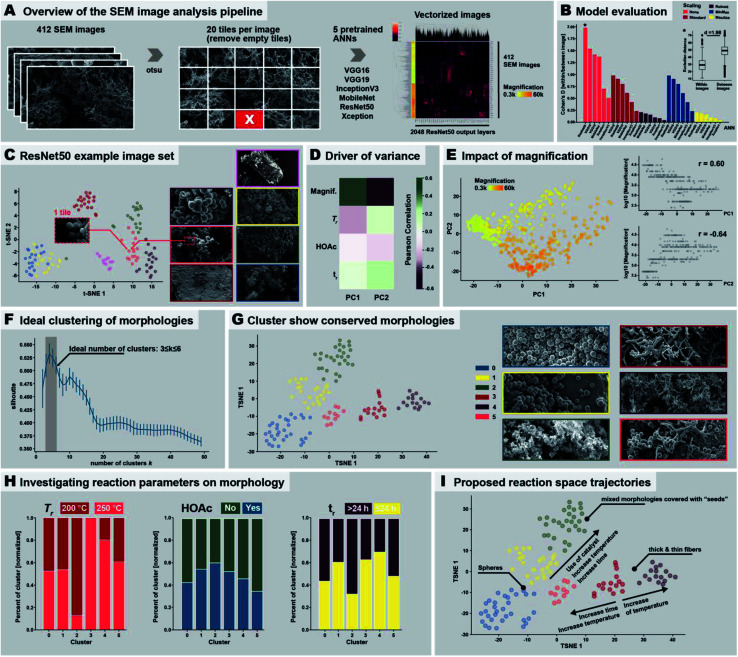
Automated SEM image analysis. (A) Image treatment and analysis pipeline employed in the study. (B) Evaluation of the performance of the different neural network models and normalization methods. (C) Result of the clustering of seven example images analyzed using ResNet50. (D) Driver of variance. (E) Impact of magnification. (F) Morphology clusters. (G) Cluster show conserved morphologies. (H) Investigating reaction parameters on morphology. (I) Proposed reaction space trajectories.

Identifying the 9 distinct morphologies and devising a hypothesis for the morphological evolution of PI(TAPB–PMA) during HTP (*cf.* Section 2.3) was ensured through manual expert interpretation of 437 SEM images. Our images are complex through often featuring several of the identified distinct morphologies in one and the same picture. Since manual interpretation is tedious, we were wondering if images as complex as ours could be analyzed more efficiently in an automated fashion. This interest is in line with current major efforts of the chemistry community: recent examples to best profit from machine support include *e.g.*, employing robotics,^38^ predicting compounds,^57^ discovering reactions,^58^ and performing and analyzing physicochemical characterizations.^59^ Computational SEM image analysis, notably automated image recognition and categorization, has been recently reported.^60–63^ Yet, the images analyzed in these prior reports are significantly less complex than our images. In fact, most computational SEM image analyses show low accuracy when confronted with images containing elements of multiple categories.^60^ Here, we aimed at automatedly clustering our SEM images by the morphologies they contain, specifically through using artificial neural networks (ANNs), a type of machine-learning methods.^64^ ANNs comprise an input layer, one or many hidden layers, and an output layer, each containing hundreds or thousands of so-called nodes. Nodes are computational devices performing a weighted simple function on a set of inputs and the outputs of the nodes are fed as inputs to other nodes.^65^ In an ANN computation, the weights of the nodes are changed iteratively (so-called learning or training), and in the end an overall output (harvested at the output layer) results from a somewhat black box (the hidden layers) in response to the initial input (fed in at the input layer).^65^ ANN approaches are well suited for image analysis in general, and to our knowledge, all reports on automated SEM image analysis employ ANN approaches.^60–63^ To implement ANNs for analyzing our SEM images, we decided to use pre-trained ANNs. Furthermore, as conventionally done for image clustering, we cut away the output layer of the used ANNs, since output layers are conventionally used for prediction (*e.g.*, in a chemical context, to predict a physicochemical property). Consequently, we analyzed the last connected hidden layer instead of the output layer. The numerical values harvested at the last hidden layer don't have any physical meaning and are therefore termed pseudo-features. Despite pseudo-features not containing any physical meaning, the input of two similar images should result in similar pseudo-feature values, which allows for clustering the input images as recently shown for the classification of cell microscopy images.^66^ The employed image treatment and analysis pipeline is displayed in Fig. 7A. First, to speed up the classification process of our SEM images, as well as to focus on local patterns of the images, we split the images into 20 tiles before ANN classification (Fig. 7A). Second, we computationally removed tiles expected to falsify the ANN analysis if used as input. Specifically, these would be (i) areas within an image that do not contain any morphology but just background, or (ii) areas that are far in the perceived back of the image (*i.e.*, out of the focused area of the image). Areas that do not contain any morphology arise from the sample preparation: our samples for SEM imaging are prepared by sprinkling a dry powder of the sample onto a carbon-tape coated sample holder prior to sputtering with a conductive alloy. Hence, there are areas of the sample which do not contain any object and display just the bare, sputtered carbon tape. Both areas devoid of objects and areas depicting objects in the far background were removed after identifying them computationally using the Otsu threshold (Fig. 7A),^67^ which allows for detecting fore- and background. This resulted in the removal of 25 images, as these did not contain any tile passing the analysis, *i.e.*, 412 images remained. Third, we analyzed the remaining 412 images' tiles using ANNs. We used 6 different ANN topologies that have been previously employed for automated image analysis, namely: ResNet50,^68^ VGG16,^69^ VGG19,^69^ IncecptionV3,^70^ MobileNet,^71^ and Xception,^72^ see Fig. 7A. These ANNs are all pretrained with the ImageNet dataset,^73^ which is an image database comprising currently >14 million hand-annotated image files conventionally used to train/test visual object recognition software. The resulting number of pseudo-features is either 512 (VGG16, VGG19), 1024 (MobileNet), or 2048 (Xception, InceptionV3, ResNet50). The pseudo-features’ numerical values were normalized (which is necessary for the absence of physical meaning). In Fig. 7A, right side, the pseudo-feature value clustering is shown, evincing already at this point that some pseudo-feature values cluster. We investigated the impact of 5 different pseudo-feature normalization methods on the overall outcome of the image classification. To test the ANN architectures and normalization methods, we used a training set of 7 images comprising both similar and dissimilar morphologies (Fig. 7C, right). Ideally, the method would put the 20 tiles of the same image close together in the *n*-dimensional pseudo-feature space (*e.g.*, 2048 dimensions in the case of ResNet50), while maximizing the distance between two dissimilar images. As benchmark test, we decided to calculate the intra-image (tiles of same image) as well as the inter-image (tiles of different images) Euclidean distances and calculated the Cohen's *D* (effect size indicating the standardized difference between two means) between the two distributions (larger values indicating better separation). We found that using the original pseudo-feature vectors resulted in better results than any of the normalization methods. Especially the scaling methods Robust and MaxAbs have a detrimental impact on SEM image classification, and result in Cohen's *D* ≤ 0.25 for all six ANNs (Fig. 7B). The best performing ANN topology was found to be ResNet50, followed by VGG16 and VGG19, and to a lesser degree MobileNet. Xception and InceptionV3 seem to be less suitable (*cf.*Fig. 7B & ESI†). Consequently, we decided to use the original pseudo-feature vector space of ResNet50 for further analysis. Applying the dimensionality reduction methodology t-SNE (stochastic neighborhood embedding) on the 2048 pseudo-features of ResNet50, we found that tiles of the same image cluster well together (Fig. 7C), while individual images are well separated. Additionally, images with similar morphologies are also clustered close together, *e.g.*, the yellow and the blue framed SEM images in Fig. 7C, which are both depicting thin fibers. Having picked ResNet50 as our ANN of choice, we next analyzed all 437 SEM images. As overall result we decided to use the centroid of the 20 tiles for each image. To investigate the resulting clustering, we used the dimensional reduction methods principal component analysis (PCA; maximizes variance) and again t-SNE (visually clusters similar objects), see Fig. 7D and E and ESI.† For both methods we found a strong link between image magnification and the clustering. Calculating the correlation between the two first principal components and magnification plus the 4 reaction parameters varied in the morphological study, *i.e.*, presence/absence of HOAc, temperature and reaction time, we found that in fact most of the variance between the images can be attributed to magnification itself (see Fig. 7D and ESI†). This is expected, as an object cannot necessarily be interpreted as the same object at different magnifications. For the strong impact of magnification, we decided to perform all subsequent analysis on a more homogenous set of images, *i.e.*, on images recorded at magnifications 1300–4000×. Within these boundaries lie 200 out of the 437 SEM images, *i.e.*, *ca.* half of the images. We applied t-SNE on this reduced set of images and performed agglomerative clustering. Agglomerative clustering requires a defined number of clusters and then recursively merges the pair of clusters that minimally increases a given linkage distance. The ideal number of clusters was identified using the silhouette method,^74^ which provides a measure for how well an object resides within its cluster (cohesion), outside of the cluster, or between clusters (separation). The silhouette value can range from −1 to +1, where a high value indicates good cohesion. Hence, an appropriate clustering method is reflected in high silhouette values, while low/negative values indicate a subpar clustering (*i.e.*, too many or too few clusters). Applying the silhouette method, we identified an ideal number of clusters *k* between 3 and 6 clusters (Fig. 7F). At 3 ≤ *k* ≤ 6, the silhouette values are >0.5. We decided to use the upper limit of the ideal cluster range, *i.e. k* = 6, to have smaller, more detailed clusters to characterize. When visually inspecting images (ESI†), we found that: (i) cluster 0 corresponds to images that almost exclusively depict near-monodisperse microspheres. (ii) Cluster 1 corresponds to images showing near-monodisperse microspheres together with smooth bulk areas (likely corresponding to agglomerated particles that stuck to the reactor wall and coalesced). (iii) Cluster 2 corresponds to images featuring mixed morphologies of a small number of spherical particles, some fibers, and some wrinkled particles, notably all decorated with the nanoparticles that we previously referred to as “seeds”. (iv) Cluster 3 corresponds to images featuring thick fibers coexisting with some spherical particles and pearl-chain morphologies. (v) Cluster 4 corresponds to images featuring mostly thin fibers, and (vi) Cluster 5 corresponds to images mainly featuring thick fibers. Fig. S37C† exemplarily shows 4 images per cluster. We found that images within one cluster show remarkable resemblance in the morphologies they contain, indicating that pretrained ANNs are in fact capable of detecting the differences in our images that correspond to the different morphologies, despite the images' morphological complexity. Furthermore, the relative distance between the individual clusters reflects the overall similarity between them (see t-SNE in Fig. 7G). Cluster 0, 1 and 3 all contain spheres and are close to each other, while cluster 3 (spheres and thick fibers) is closer to cluster 5 (mainly thick fibers).

Next, we investigated if the computationally determined 6 morphological clusters correlate with certain reaction conditions, as we have in the previous section devised by manual expert interpretation of the SEM images. Therefore, we checked the percentage of morphologies within each cluster being associated with a certain reaction parameter (*cf.* ESI†). Since reaction conditions are not equally balanced in our image set, *e.g.*, 81 images were obtained from products of syntheses at 200 °C *vs.* 47 images at 250 °C, we normalized the results accordingly (see ESI,† computational methods). We checked for the three varied reaction parameters, *i.e.*, temperature (200 °C *vs.* 250 °C), use of HOAc (yes *vs.* no), and reaction time (short ≤ 24 h and long > 24 h), see Fig. 7H (ESI† for absolute numbers). As becomes clear from Fig. 7H left, cluster 3 (thick fibers coexisting with some pearl-chain morphologies) exclusively contains images of samples that have been synthesized at 200 °C. On the contrary, cluster 2 (images of mixed morphologies covered with “seeds”) is almost exclusively found at 250 °C. Strikingly, this is in agreement with our manual interpretation that thick fibers and pearl-chain morphologies go hand in hand with the early/low-*T*_r_ stages of the HTP of PI(TAPB–PMA), while “seed”-decorated morphologies are characteristic for silica/hybrid formation at high *T*_r_ and long *t*_r_. Regarding the presence of HOAc (Fig. 7H, middle), we computationally find that cluster 2 (mixed morphologies covered with “seeds”) benefits most from the presence of HOAc, while cluster 0 (exclusively near-monodisperse spheres) and 5 (mainly thick fibers) are more connected to the absence of HOAc. This too agrees with our previous observation that thick fibers are rather found when HOAc is not present, and, regarding “seeds”, and with the prior reports that the presence of acids catalyzes silica dissolution. With respect to *t*_r_, thick fibers (cluster 3) and thin fibers (cluster 4) are predominantly found at *t*_r_ ≤ 24 h, while seed-covered morphologies (cluster 2) predominantly form at long *t*_r_. This is again in agreement with our manual interpretation. Finally, we compiled these results into proposed reaction trajectories (see Fig. 7I): starting from thick fiber morphologies that are formed at 200 °C relatively fast (≤24 h), increasing *T*_r_ slightly results in thin fiber morphologies, while even higher increase in *T*_r_ as well as time increases the chance for spherical morphologies. Increasing both *t*_r_ and *T*_r_ as well as adding a HOAc increases the likelihood of generating “seed”-covered morphologies.

Taken together we could show that ANNs are indeed capable of detecting the subtle differences that lie within morphologically complex SEM images, managing to differentiate such mixed morphologies. We identified that the unnormalized space of ResNet50 pseudovectors as for our research question best way to analyze these morphologies. Furthermore, we have shown that while certain ranges in magnification can be compensated by this technology, too large differences cannot. Also, the use of dimensional reduction technologies such as PCA and t-SNE as well as standard clustering techniques such as agglomerative clustering offer fast and easy tools to successfully automatically detect clusters of morphologies. We moreover showed how once successfully identified, one can investigate the role of reaction parameters on the formation of such morphologies, even potentially inferring reaction trajectories. While manual annotation of these morphologies of SEM images is time consuming the automated analysis manages to detect clusters of images that are based on the morphologies within several minutes (depending on the hardware; *ca.* 30 min on a Macbook Pro), offering an easy scalable unbiased analysis for such complex SEM images. The automated analysis detects only part of the morphologies that we have manually identified, yet, this is likely to be surmountable by an increased fine-graining (higher number of image tiles), and a full analysis of all images in appropriate subgroups subdivided by magnification ranges.

### 2.5 MW-assisted synthesis and upscaling of PI(TAPB–PMA) microspheres and their sintering to pellets

**Fig. 8:**
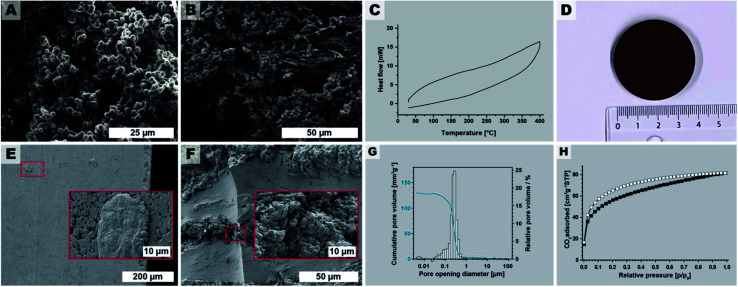
Upscaling and processing. (A) SEM image of PI(TAPB–PMA) synthesized at 200 °C, *t*_r_ = 4 h, in a st reactor. (B) SEM image of the upscaled PI(TAPB–PMA), 250 °C/4 h in stirred 100 mL microwave vessels (4 syntheses in parallel). (C) DSC of the upscaled PI(8APB-PMA). (D) Photograph of the PI(TAPB–PMA) pellet obtained by warm-pressing. (E) SEM images of the outer surface of the pellet. (F) SEM image of a fracture surface. (G) Pore opening diameter distribution from Hg porosimetry; (H) low pressure CO_2_ physisorption isotherm measured at 195 K (adsorption: white squares; desorption: black squares).

Stirring during materials synthesis in the liquid phase is generally known to influence the materials' morphology and morphological homogeneity. Our reaction parameter screening discussed in Sections 2.2 and 2.3 was carried out in non-stirred batch autoclaves and at short *t*_r_, led to monoliths composed of spherical microparticles (major component) and microfibers (minor component). Therefore, we expected that employing stirring during HTP would exclusively generate microspheres. To test this hypothesis, we performed several HTP experiments at *T*_r_ = 200 °C, without HOAc, and at *t*_r_ = 4 h in a stirred microwave (MW) reactor (V(H_2_O) = 10 mL, *V* (vessel) = 30 mL, see ESI†). We judged *t*_r_ = 4 h as being sufficient, since (i) MW-assisted heating is generally more efficient than heating batch autoclaves by placing them in ovens, and (ii) our reaction parameter screening had shown that *t*_r_ = 2 h was sufficient to form PI(TAPB–PMA). The thereby synthesized PI(TAPB–PMA) was reproducibly obtained as powders of exclusively near-monodisperse spherical microparticles (see Fig. 8A and ESI†). As expected, the monolithic structure obtained in batch reactors at short *t*_r_ was not retained. For the spherical morphology of the microparticles, and the other roundish morphologies (thick fibers, pearl-chain morphologies) discussed in Section 2.3, we speculated that PI(TAPB–PMA) features some degree of flexibility on a molecular level, which might allow for creep at increased temperatures in the dry state. Therefore, sintering into bigger objects should be possible. For this, the near-monodisperse particles obtained through MW-assisted HTP would provide nicely homogeneous starting powders. As bigger amounts are necessary for sintering, we scaled the stirred MW-assisted HTP up by moving from 30 mL to 100 mL vials in an MW reactor featuring a carousel for simultaneously running 4 experiments (see ESI†). Furthermore, we decided for the upscaling experiments to increase the temperature, since in our experience the heat transfer in the bigger MW with several vessels is less efficient. Specifically, we chose *T*_r_ = 250 °C and *t*_r_ = 4 h. In total 4 runs of 4 × 4 vessels were performed, and cumulatively, we generated 7.364 g of PI(TAPB–PMA) as fine brown powder. SEM analysis (Fig. 8B and ESI†) revealed products consisting of mixtures of thick fiber and microsphere morphologies. The micromorphologies of PI(TAPB–PMA) from different vessels and runs did not show micromorphological differences. All vials' products were furthermore checked by ATR-FT-IR, which confirmed that all PI(TAPB–PMA) samples were fully condensed. Differential scanning calorimetry (DSC) of the upscaled PI(TAPB–PMA) measured from 30 to 400 °C, does not show any thermal phenomena upon heating or cooling, *i.e.* neither first order (peaks) nor higher order (deflection points) phase transitions (*cf.*Fig. 8C). While the absence of a *T*_g_ in until 400 °C as judged from the DSC trace was discouraging, we were still hypothesizing that sintering could be possible, again, as the roundish morphologies imply that PI(TAPB–PMA) possesses the ability to soften. We attempted sintering by warm-pressing, a technique widely used for the compaction of powder-based polymers and polymer-derived materials.^75^ Therefore, the entire 7.364 g of PI(TAPB–PMA) powder were introduced into a steel cavity with an inner diameter of 40 mm and were uniaxially compacted at 80 MPa. As release agent between the steel punches and the powder, Kapton foil was used. The temperature of the die was increased to 350 °C (heating rate 6 K min^−1^), and the maximum temperature was held for 2 h. The pressure was adjusted accordingly when required. After cooling to room temperature, the pressure was reduced and the compacted part was removed from the mold. Thereby an intact, macroscopically highly homogenous and smooth cylindrical specimen of diameter of *d* = 39.96 mm and height *h* = 5.06 mm was obtained (Fig. 8D). This corresponds to a bulk density of 1.09 g cm^−3^ of the pellet. SEM images of the specimen's outer surface (Fig. 8E) reveal (i) that the particles are indeed well consolidated and merged, yet, (ii) the initial spherical morphologies are still discernible. SEM analysis of fracture surfaces after mechanical testing (Fig. 8F) support this assessment. To evaluate how the materials' porosity had developed through compacting we again performed Hg intrusion porosimetry (Fig. 8G). The data reveals that the major part of porosity is in the size range of 0.2 to 0.5 μm. This pore population corresponds, as we believe, to the voids located at the triple-points between compressed particles. Indeed SEM images show such pore necks characteristic for an intermediate stage of sintering of spherical particles (Fig. 8E and F). The pore population furthermore depicts only minor contributions from mesoporosity. The total porosity obtained from Hg intrusion porosimetry after compressibility correction is 15%; however, due to method-inherent limitations, this value does not include pores smaller than 4 nm. Low-pressure carbon dioxide (CO_2_) sorption measurements at 195 K (Fig. 8H) of a piece of the specimen gives SA_BET_ = 203 m^2^ g^−1^, which is within the error margin of SA_BET_ = 207 m^2^ g^−1^ for the noncompacted particulate sample. Hence, one can conclude that PI(TAPB–PMA)s intrinsic ultramicroporosity is not affected by the processing *via* sintering. Finally, we tested the pellet mechanical performance by flexural strength measurements using a three-point flexural test setup, following EN ISO 178 (ESI†). The flexural strength of warm-pressed PI(TAPB–PMA) material was 36.0 ± 1.8 MPa. The flexural modulus was 2084 ± 34 MPa. The variability between individual specimens was low, suggesting an overall high homogeneity within the sample microstructure after warm-pressing. Flexural yields strengths of ∼25–500 MPa and flexural moduli of ∼1.3–40 GPa are found for commercial PIs, which places the PI(TAPB–PMA) specimen within the typical range, but at the lower end of performance. We think that the slightly decreased mechanical properties compared to literature can be traced to the residual porosity present within the samples, as shown by mercury intrusion porosimetry data.

## 3. Conclusions

With this contribution we show for the first time that HTP can generate fully condensed PI networks. The process is a fast (full condensation achieved in as short as 2 h), easy (the synthesis consists of nothing but mixing the comonomers in water, heating that dispersion in an autoclave to *T*_r_, keeping it there for *t*_r_, and isolating the product by filtration) and benign (through using solely water as the reaction medium). The synthesized PI network shows high thermal stabilities of >550 °C. At relatively short *t*_r_ the products are obtained as sponge-like monoliths, and at longer *t*_r_ as powders. We studied the sorption properties of PI(TAPB–PMA) using CO_2_ and N_2_ as analyzing gases, as well as employing Hg porosimetry measurements. These analyses revealed a trimodal porosity, comprising (i) ultra-micropores (<0.8 nm), (ii) a small amount of mesopores (<50 nm), and (iii) a majority of macropores (1 μm–100 μm) resulting in a surprisingly high specific pore volume of 7250 mm^3^ g^−1^. Furthermore, we observed a complex micromorphological evolution that manifests as a function of the studied reaction parameters (*t*_r_, *T*_r_, presence/absence of HOAc). Observed morphologies comprise fiber-like structures and near monodisperse spherical particles as well as intermediate structures such as pearl-chain morphologies. At long *t*_r_ (>48 h at 250 °C), silica dissolves from the glass liners in which the reactions were performed, and as we hypothesize, at even longer *t*_r_ reacts with the present PI networks towards hybrid materials. We furthermore developed an automated computational image analysis pipeline, which confirmed the morphology evolution determined previously by manual expert interpretation of ∼450 SEM images. Finally, using MW-assisted stirred HTP, we show that PI(TAPB–PMA)'s morphology can be controlled towards obtaining exclusively near-monodisperse microparticles. Upscaling these stirred MW- assisted HTP experiments, we could prepare a sufficient amount of microspheres to process them into a dense cylindrical specimen by warm-pressing. Compared to conventional PI processing, which uses harsh and armful solvents such as NMP or cresols, warm-pressing is benign for using solely heat and pressure. The specimen from warm-pressing retained PI(TAPB–PMA)'s intrinsic micro- and ultramicroporosity, while the majority of macropores are lost through compacting.

## Author contributions

This project was designed by MMU. ML performed all small-scale syntheses as well as all PXRD, TGA, and ATR-FT-IR measurements, and part of the SEM analyses. HM performed part of the SEM analyses as well as EDX analyses. DACI performed upscaling syntheses, as well as ATR-FT-IR and DSC analyses of the upscaled samples. TK performed and analyzed Hg porosimetry, performed processing by warm-pressing, and mechanical characterization of the specimen. JR and AT performed gas sorption measurements and analyzed the data. MC and JM performed the computational image analysis. MMU and ML wrote the manuscript, with input from all coauthors. ML, DACI and MMU prepared the figures. All authors have given approval to the final version of the manuscript.

## Conflicts of interest

There are no conflicts to declare.

## Supplementary Material

TA-009-D1TA01253C-s001
