# Supplementary material for: Hydrothermal polymerization of porous aromatic polyimide networks and machine learning-assisted computational morphology evolution interpretation
Source: J Mater Chem A Mater. 2021 Sep 8;9(35):19754–69. doi: 10.1039/d1ta01253c (PMC8439099; doi:10.1039/d1ta01253c)
Supplement: TA-009-D1TA01253C-s001 [file TA-009-D1TA01253C-s001.pdf]

Electronic Supplementary Material (ESI) for Journal of Materials Chemistry A.
This journal is © The Royal Society of Chemistry 2021

Supporting Information to the Paper

Hydrothermal Polymerization of Porous Aromatic Polyimide Networks and Machine Learning-Assisted Computational Morphology Evolution Interpretation

By

Marianne Lahnsteiner,[a,b,c] Michael Caldera,[c,d] Hipassia M. Moura,[a,b,c,e] D. Alonso Cerrón-Infantes,[a,b,c] Jérôme Roeser,[f] Thomas Konegger,[g] Arne Thomas,[f] Jörg Menche,[c,d] and Miriam M. Unterlass[a,b,c,e*]

[a] Technische Universität Wien, Institute of Materials Chemistry, Getreidemarkt 9/165, 1060 Wien, Austria.

[b] Technische Universität Wien, Institute of Applied Synthetic Chemistry, Getreidemarkt 9/163, 1060 Wien, Austria.

[c] CeMM - Research Center for Molecular Medicine of the Austrian Academy of Sciences, Lazarettgasse 14, AKH BT 25.3, 1090 Wien, Austria.

[d] Max F. Perutz Labs, Campus Vienna Biocenter 5, Dr.-Bohr-Gasse 9, 1030 Vienna, Austria

[e] Universität Konstanz, Department of Chemistry, Solid state chemistry, Universitätsstrasse 10, D-78464 Konstanz, Germany.

[f] Technische Universität Berlin, Institute of Chemistry, Str. des 17. Juni 115, 10623 Berlin, Germany.

[g] Technische Universität Wien, Institute of Chemical Technologies and Analytics, Getreidemarkt 9/164, 1060 Vienna, Austria

[*]To whom correspondence should be addressed: miriam.unterlass@uni-konstanz.de

Table of Contents

1. EXPERIMENTAL DETAILS 3

1.1. CHEMICALS AND EQUIPMENT 3

1.2. GENERAL PROCEDURE 3

1.2.1. MONOMER SALT SYNTHESIS 3

1.2.2. HYDROTHERMAL POLYMERIZATION 4

1.2.3. MICROWAVE SYNTHESIS 4

1.2.4. SOLID STATE POLYMERIZATION 4

2. CHARACTERIZATION 5

2.1. ATR-FT-IR 5

2.2. THERMOGRAVIMETRIC ANALYSIS 9

2.3. SCANNING ELECTRON MICROSCOPY 16

2.3.1. SEM MONOWAVE SYNTHESIS OF PI(TAPB-PMA) 17

2.3.2. SEM MULTIWAVE UPSCALE SYNTHESIS PI(TAPB-PMA) 18

2.3.3. SEM SOLID-STATE POLYMERIZATION 19

2.4. LOW PRESSURE GAS SORPTION MEASUREMENTS 20

2.5. HG- POROSIMETRY 26

2.6. POWDER X- RAY DIFFRACTOMETRY 26

2.7. DIFFERENTIAL SCANNING CALORIMETRY DSC 33

2.8. LITERATURE COMPARISON 34

2.9. AUTOMATED IMAGE ANALYSIS 35

2.10. ENERGY-DISPERSIVE X-RAY SPECTROSCOPY (EDAX) 38

3. PROCESSING 47

3.1. WARM-PRESSING 47

3.2. FLEXUAL STRENGTH EVALUATION 47

1. Experimental details

1.1. Chemicals and equipment

All chemicals were commercially available and used as received from TCI without any further purification. Deionized water was used at all times. As reaction vessels Berghof Digestec system and Parr steel reactors were used. ^{1}H NMR spectra were recorded on a Bruker AC600 spectrometer. Chemical shifts are reported in ppm (δ) relative to tetramethylsilane and calibrated using solvent residual peaks. Data are shown as follows: Chemical shift, multiplicity (s = singlet, d = doublet, t = triplet, q = quartet, quin = quintet, m = multiplet, b = broad signal), coupling constant (J, Hz) and integration.

1.2. General procedure

1.2.1. Monomer salt synthesis

TAPB (1 eq., 1 mmol) and PMA (1.5 eq., 1.5 mmol) were weighed in to a round bottom flask and suspended in 10 mL of deionized water. The white suspension was stirred at room temperature overnight. In the course of the reaction, the suspension turned green. The solid was filtered, washed with water and very little acetone and subsequently dried *in vacuo*. Yield: 785,3 mg (54%). ^{1}H NMR (600 MHz, DMSO-d_6) δ 8.02 (s, 6H), 7.52 (s, 6H), 7.51 (d, 12H), 6.73 (d, J = 8.4 Hz, 12H) ppm.

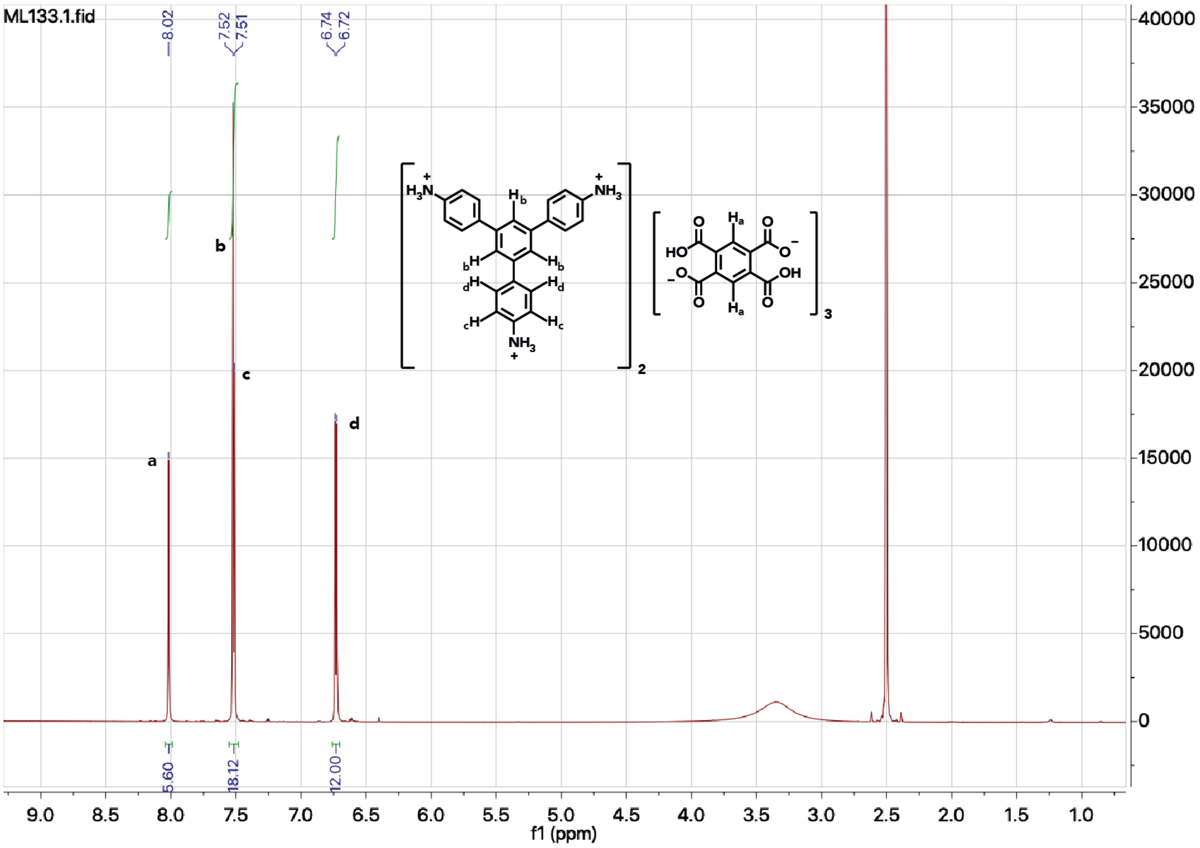

Figure S 1 NMR spectrum of the multitopic monomer salt [$H_3TAPB^{3+}PMA^{2-}$]

1.2.2. Hydrothermal polymerization

TAPB (1 eq., 0.2 mmol) and PMA (1.5 eq., 0.3 mmol) were weighed into a glass liner and suspended in 10 mL H_2O. After ultrasonication for 10 min, the glass liner was sealed in a steel autoclave and heated to 200 °C or 250 °C for 2-168 h under autogenous pressure (~18 bar). The brownish precipitate was filtered *via* a Büchner funnel (13-15 µm particle retention) and washed with water, acetone (techn.) and THF. Afterwards 75 mg of the product (ranging from brown powder to spongy monolith depending on the conditions) was dried at 80 °C in a vacuum oven overnight.

1.2.3. Microwave synthesis

Monowave

TAPB (1 eq., 0.2 mmol) and PMA (1.5 eq., 0.3 mmol) were weighed into a microwave reactor (Anton Paar, G30) and suspended in 10 mL H_2O. After adding a stirring bar, the reactor was closed with a cap including a PTFE septum and placed in the Anton Paar Monowave 400. The vessel was heated as fast as possible to 200 °C, stirred with 600 rpm, and held at that temperature for 4 h. Upon heating the reaction mixture turned first green, then blue and finally, when the temperature was reached, a brown precipitate was formed. Afterwards, the reactor was cooled down to 70 °C and the brown precipitate was collected via filtration. The product was washed with water, acetone, ethanol and THF. Finally, the product (brown powder, 73 mg) was dried overnight, *in vacuo*, at 80 °C.

Multiwave Pro

TAPB (1 eq., 0.8 mmol) and PMA (1.5 eq., 1.2 mmol) were weighed into a microwave reactor (Anton Paar, N8QX), suspended in 40 mL H_2O (degassed with Ar) and a stirring bar was added. In total four of these reactors were put in to the Anton Paar Multiwave Pro and heated within 20 min to 250 °C and held there for 4 h while stirring on the highest level. Afterwards, the reactors were cooled down to 70°C using compressed air. The reactors were opened, the brown powders of all four reactors were collected via filtration, washed with water, EtOH and acetone. The procedure was repeated four times and the 16 batches were combined. The product was then dried *in vacuo*. Yield: 7.364 g.

1.2.4. Solid state polymerization

500 mg of [$H_3TAPB^{3+}PMA^{2-}$] were placed in a round bottom flask, containing a stirring bar and evacuated. Afterwards, while continuously applying vacuum, the flask was placed in an oil bath which was set at 200 C° for 8 h while stirring vigorously. A color change from pale green to yellow and finally to beige was observed after several hours of heating. When cooled down to rt, the beige solid powder (360 mg) was collected and stored.

2. Characterization

2.1. ATR-FT-IR

IR-measurements were recorded with a PerkinElmer UATR Two FT-IR spectrometer and the data was processed with the PerkinElmer Spectrum software.

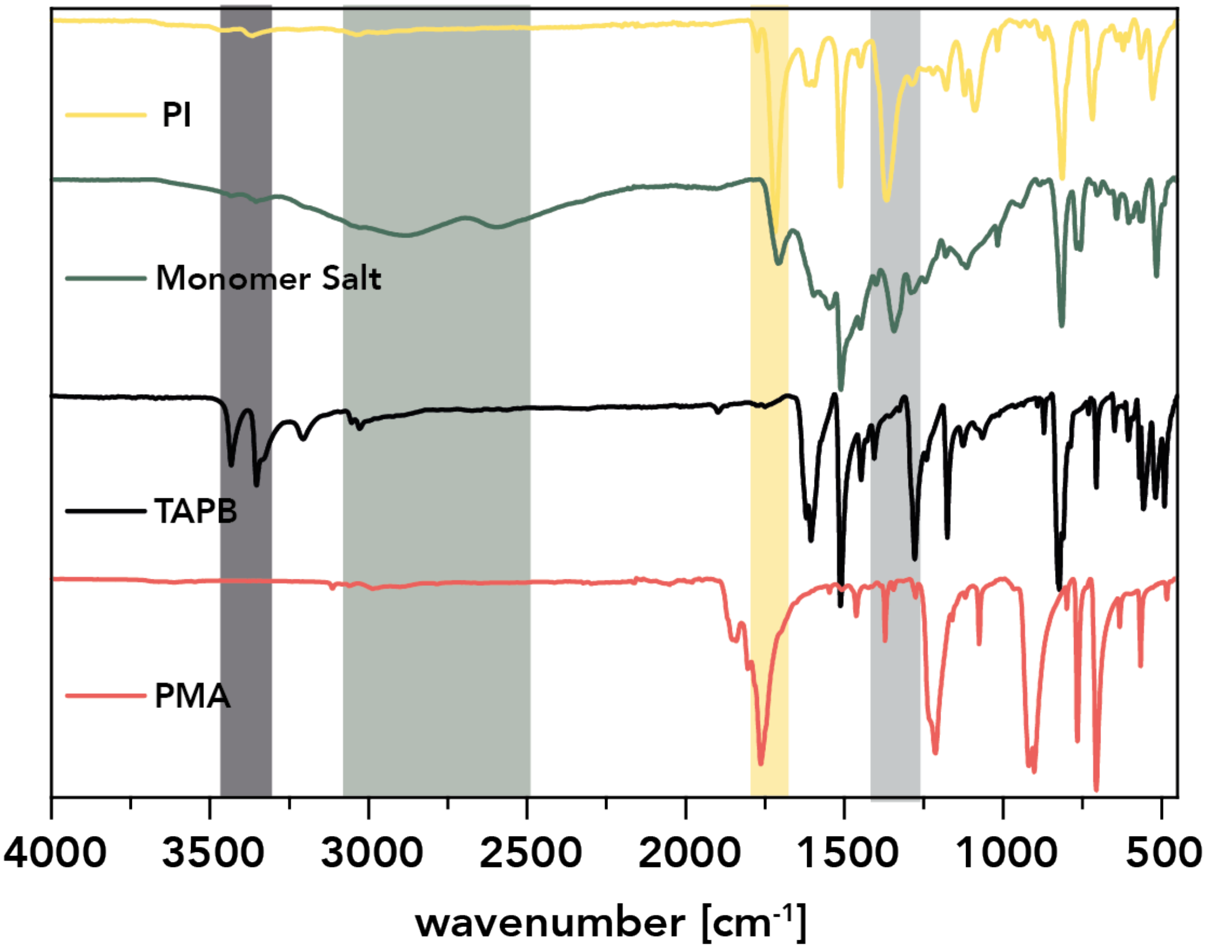

Figure S 2 ATR-FT-IR spectra of the monomer salt (green, aryl-ammonium modes at 2876 and 2592 cm^{-1}), a PI sample (t_r = 24 h, T_r = 200°C; symmetric and asymmetric C=O stretches at 1775 and 1720 cm^{-1} as well as the C-N stretch at 1365 cm^{-1}) and the two precursors TAPB (free amine modes at 3400-3300 cm^{-1}) and PMA (C=O stretches).

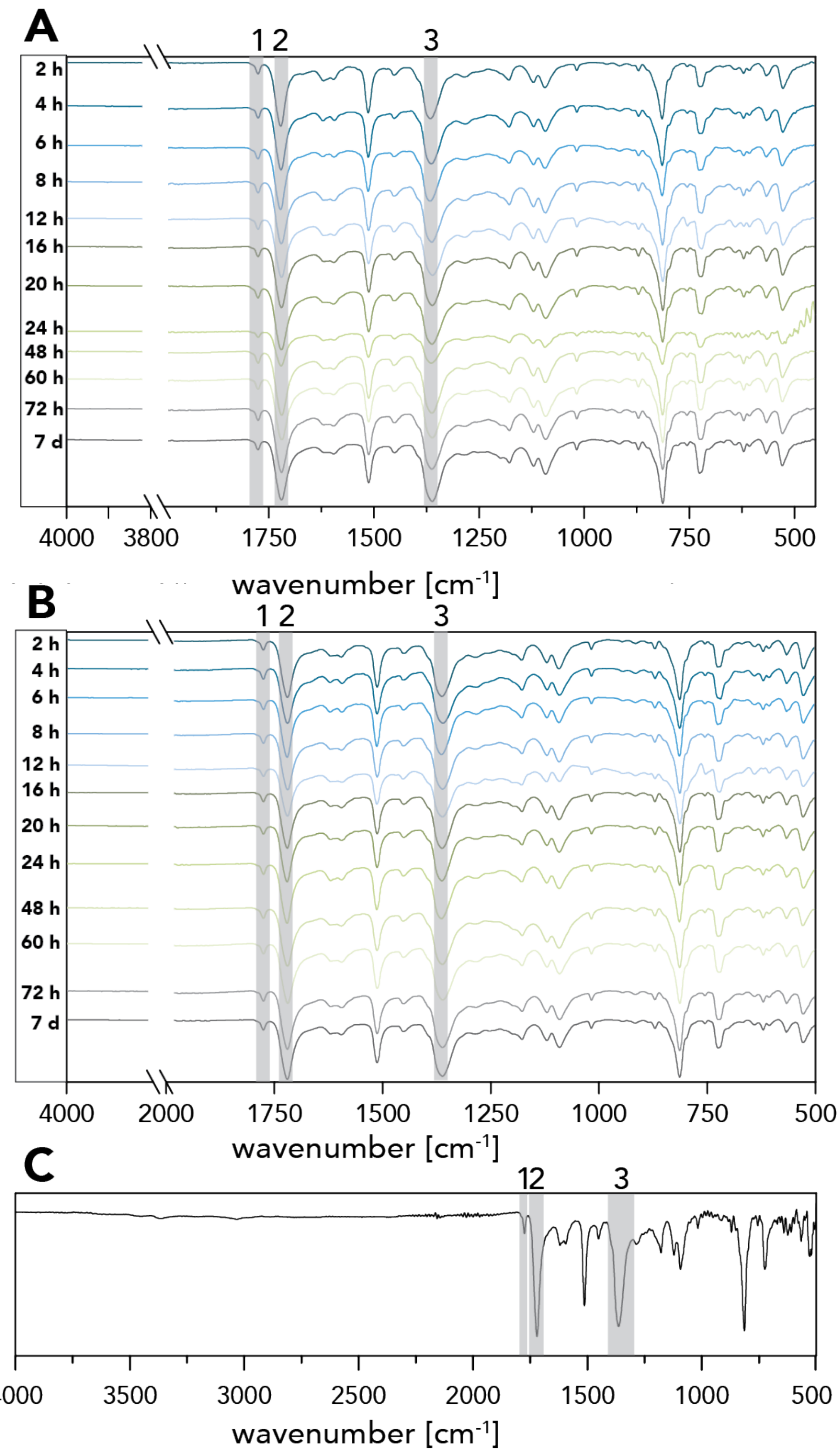

Figure S 3 **A** ATR-FT-IR spectra of all samples synthesized at 200 °C without HOAc. **B** ATR-FT-IR spectra of all samples synthesized at 200 °C in presence of HOAc. **C** a representative full spectrum. **1** symmetric C=O stretch at 1775 cm^{-1}, **2** asymmetric C=O stretch at 1720 cm^{-1}. **3** C-N stretch at 1365 cm^{-1}. Together these 3 modes confirm the presence of an imide.

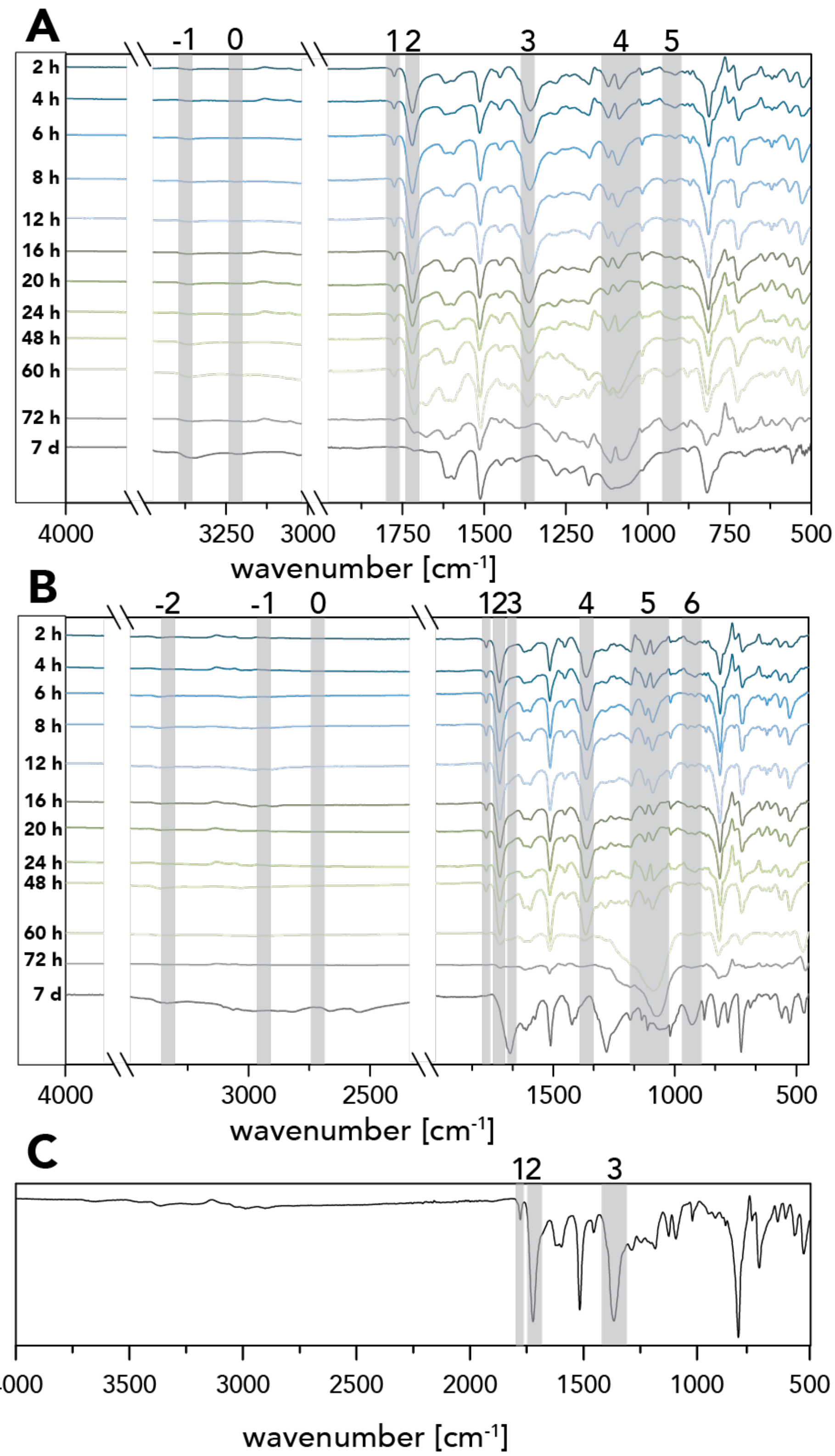

Figure S 4 A ATR-FT-IR spectra of all samples synthesized at 250 °C without HOAc. **1** symmetric C=O stretch at 1775 cm^{-1}, **2** asymmetric C=O stretch at 1720 cm^{-1}, **3** C-N stretch at 1365 cm^{-1} **4** Si-O-Si at 1125-1050 cm^{-1} and **5** Si-OH at 950-900 cm^{-1}. **B** ATR-FT-IR spectra of all samples synthesized at 250 °C in presence of HOAc. **1** symmetric C=O stretch at 1775 cm^{-1}, **2** asymmetric C=O stretch at 1720 cm^{-1}, **3** C=O stretch at 1520 cm^{-1}, **4** C-N stretch at 1365 cm^{-1}, **5** Si-O-Si at 1125-1050 cm^{-1} and **6** Si-OH at 950-900 cm^{-1}. **C** a representative full spectrum. **1** symmetric C=O stretch at 1775 cm^{-1}, **2** asymmetric C=O stretch at 1720 cm^{-1}. **3** C-N stretch at 1365 cm^{-1}. Together these 3 modes confirm the presence of an imide. In all spectra **-2, -1,** and **0** could be possible endgroups like amines or acides.

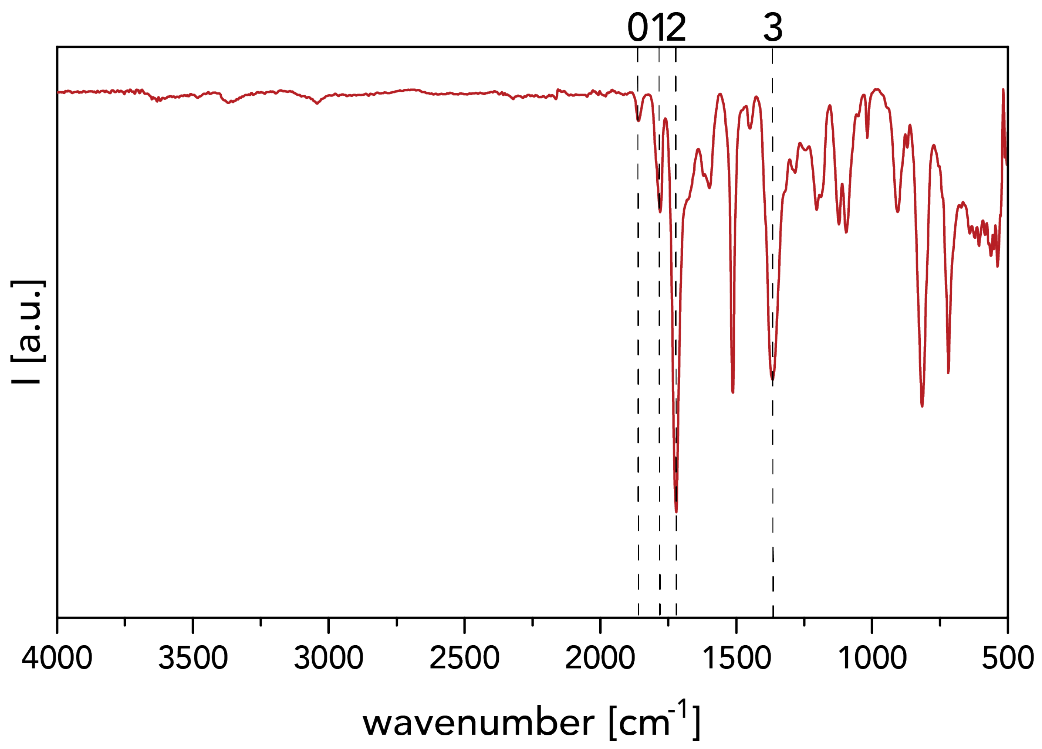

Figure S 5 ATR-FT-IR spectrum of the solid-state polymerization synthesis of PI(TAPB-PMA). **0** 1860 cm^{-1} which is most likely a C=O mode arising from pyromellitic anhydride (which is condensing during the solid-state polymerization process). **1** 1780 and **2** 1720 cm^{-1} are the symmetric and asymmetric stretching modes and **3** 1365 cm^{-1} the C-N mode of the imide moiety.

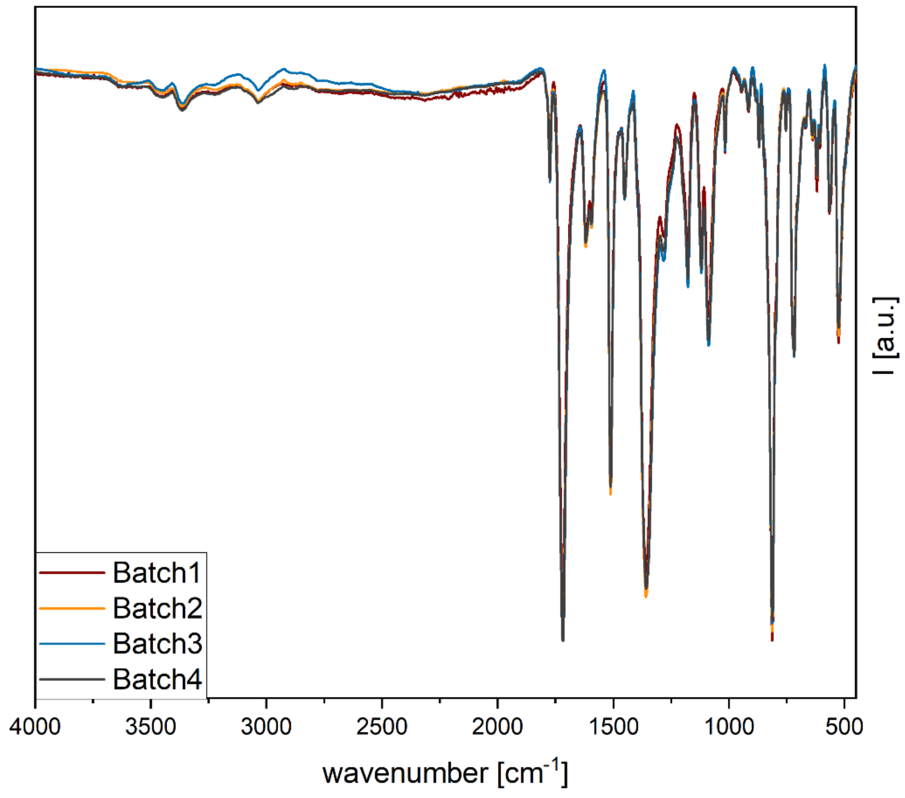

Figure S 6 ATR-FT-IR of the 4 PI(TAPB-PMA) batches obtained from the upscale reactions using stirred microwave reactors.

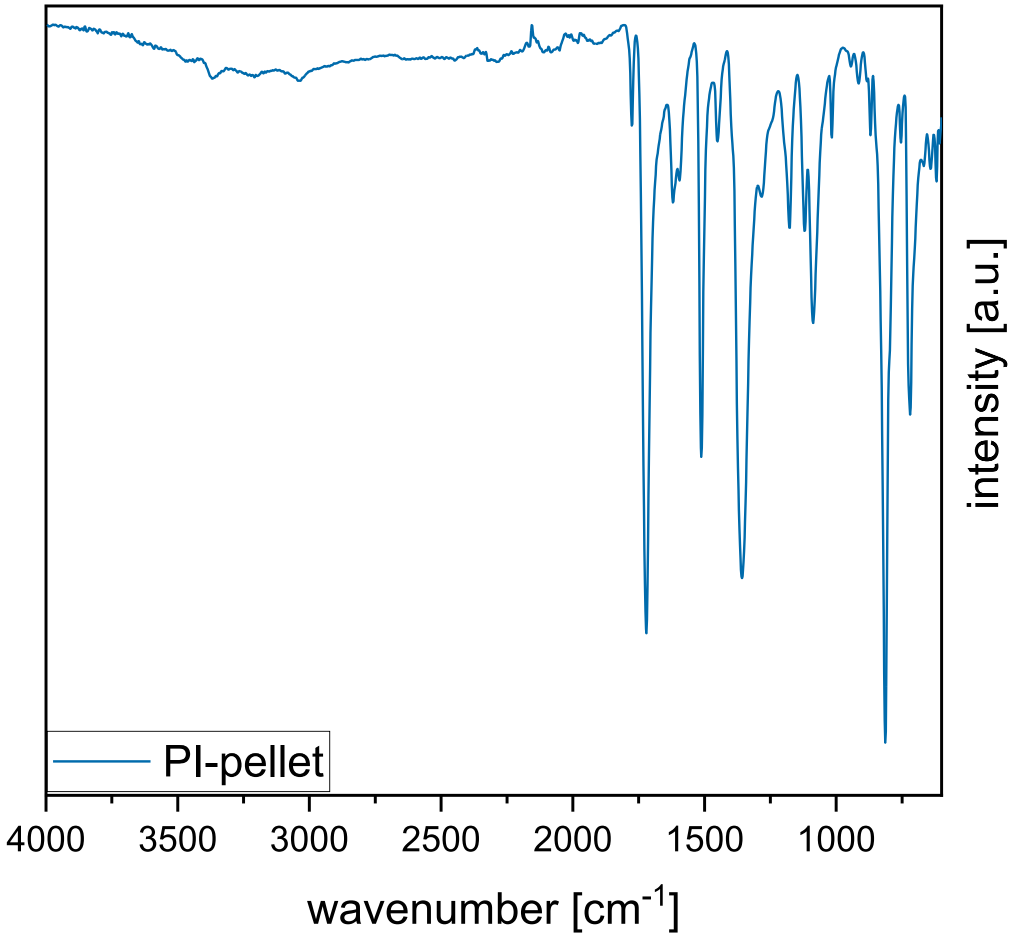

Figure S 7 ATR-FT-IR of the warm pressed PI(TAPB-PMA) aka pellet.

2.2. Thermogravimetric Analysis

Thermogravimetric analysis was carried out using a Netzsch TG 209 analyzer at a heating rate of 10 K min^{-1} under nitrogen atmosphere, equipped with NETZSCH Proteus (Version 4.3) software.

Table S1 Decomposition temperatures for all PI networks synthesized within the reaction screening determined via the tangent method.

t_r [h]	T_r [°C]	HOAc	T_d [°C]	t_r [h]	T_r [°C]	HOAc	T_d [°C]
2	200	no	552	2	200	yes	548
4	200	no	545	4	200	yes	544
6	200	no	554	6	200	yes	546
8	200	no	584	8	200	yes	591
12	200	no	551	12	200	yes	559
16	200	no	560	16	200	yes	562
20	200	no	567	20	200	yes	582
24	200	no	562	24	200	yes	591
48	200	no	567	48	200	yes	576
60	200	no	575	60	200	yes	572
72	200	no	564	72	200	yes	562
168	200	no	596	168	200	yes	586
2	250	no	545	2	250	yes	587
4	250	no	555	4	250	yes	562
6	250	no	554	6	250	yes	572
8	250	no	564	8	250	yes	569
12	250	no	564	12	250	yes	598
16	250	no	555	16	250	yes	559
20	250	no	587	20	250	yes	590
24	250	no	596	24	250	yes	550
48	250	no	528	48	250	yes	542
60	250	no	537	60	250	yes	533
72	250	no	252	72	250	yes	254
168	250	no	255	168	250	yes	238

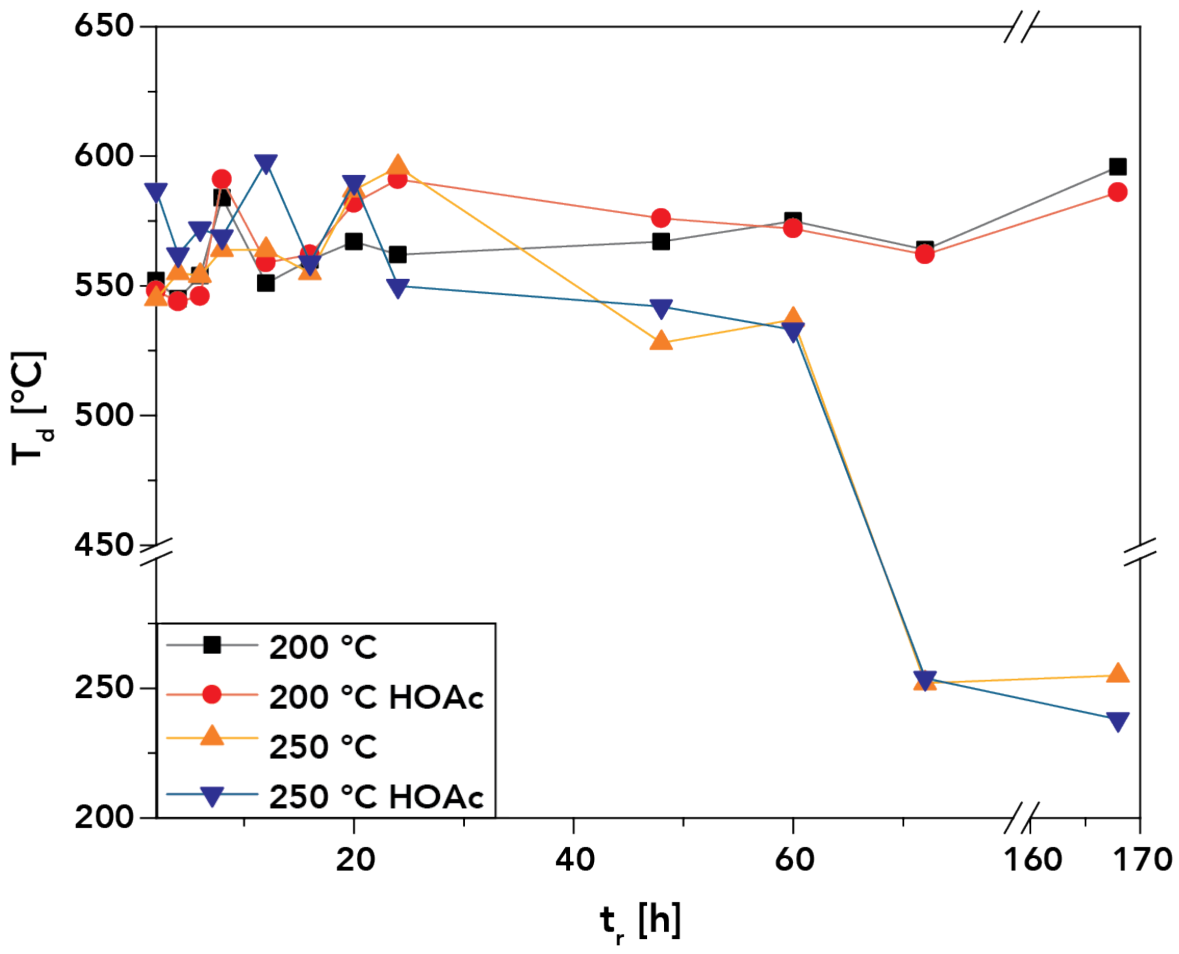

Figure S 8 T_d plotted versus reaction time of all samples studied within the screening (200 °C, 250 °C, with and without modulator). At lower T the stability definitely increases with t_r regardless the addition of modulator. At higher T, the materials' stability drastically decreases, which leads to the assumption that the polymer degraded already during its synthesis.

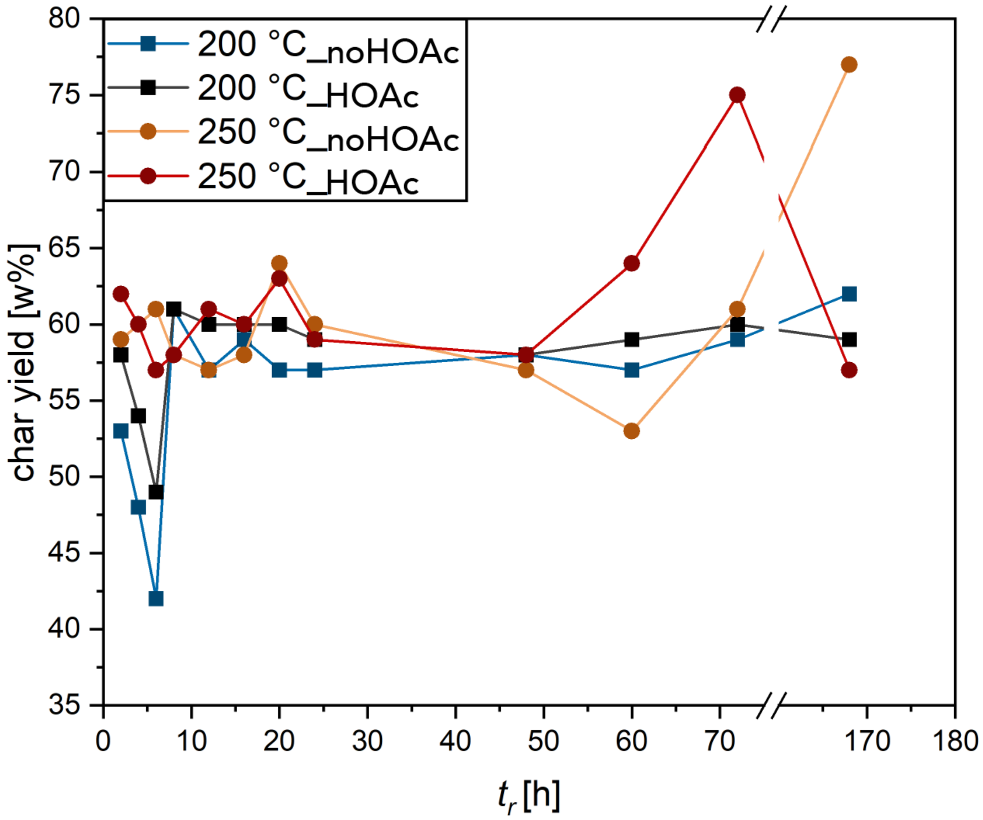

Figure S 9 Char yields in w% of all samples after TGA analysis until 900 °C. A clear trend towards higher char yields with higher t_r is visible, with the exception of the sample synthesized at 250 °C, 168 h with HOAc.

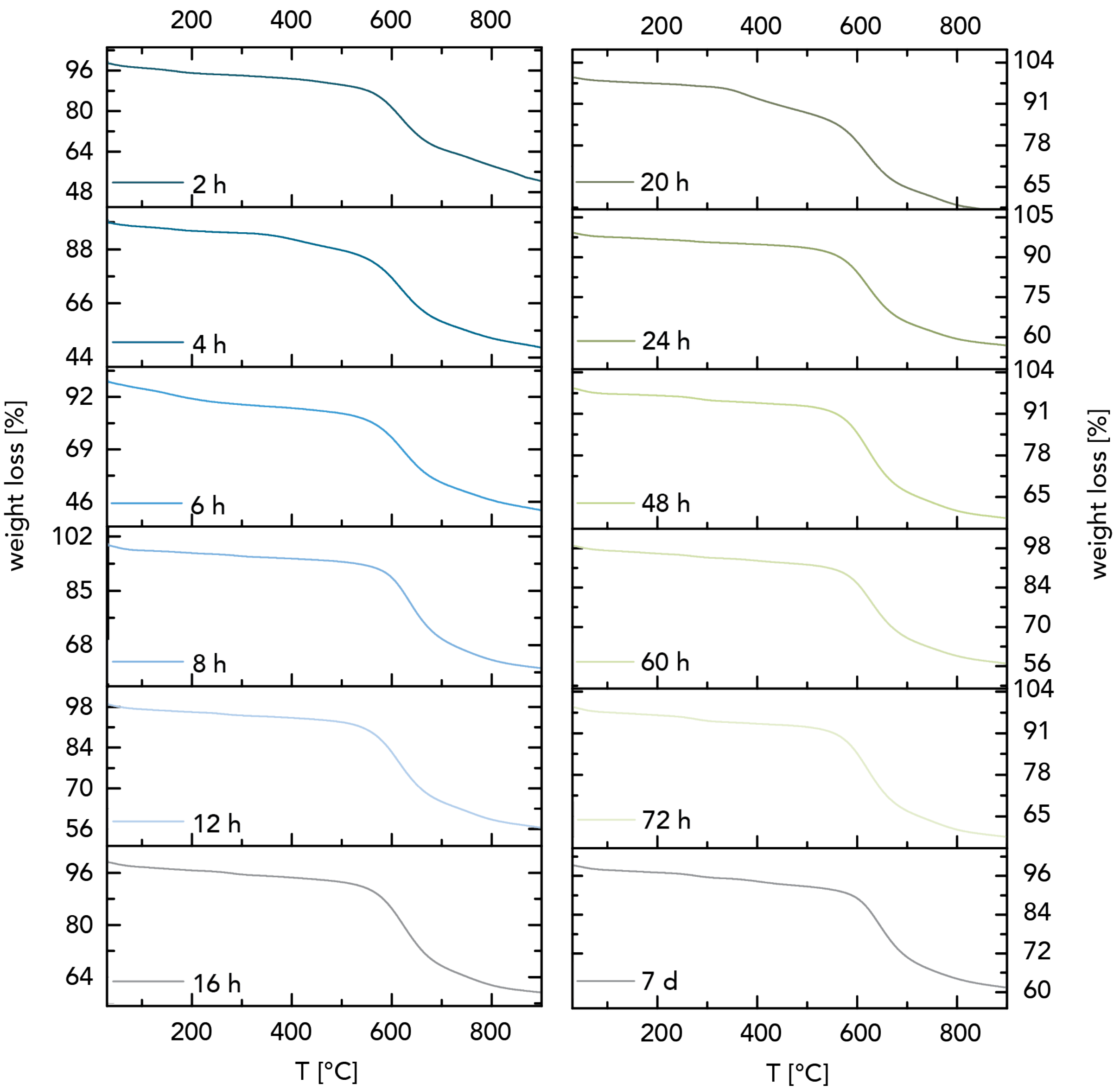

Figure S 10 TGA measurements of all samples synthesized at 200°C without the addition of HOAc.

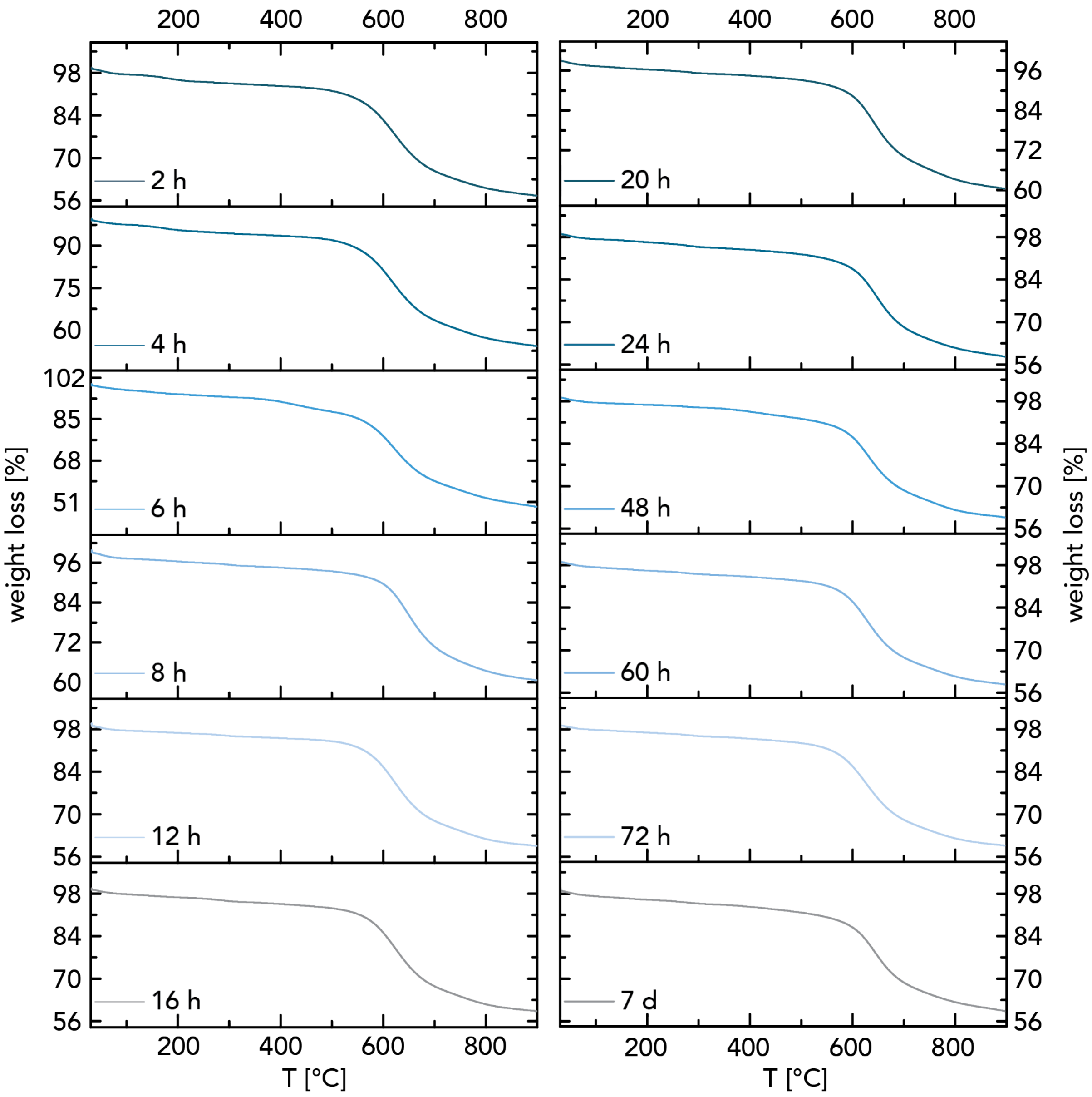

Figure S 11 TGA measurements of all samples synthesized at 200°C in the presence of HOAc.

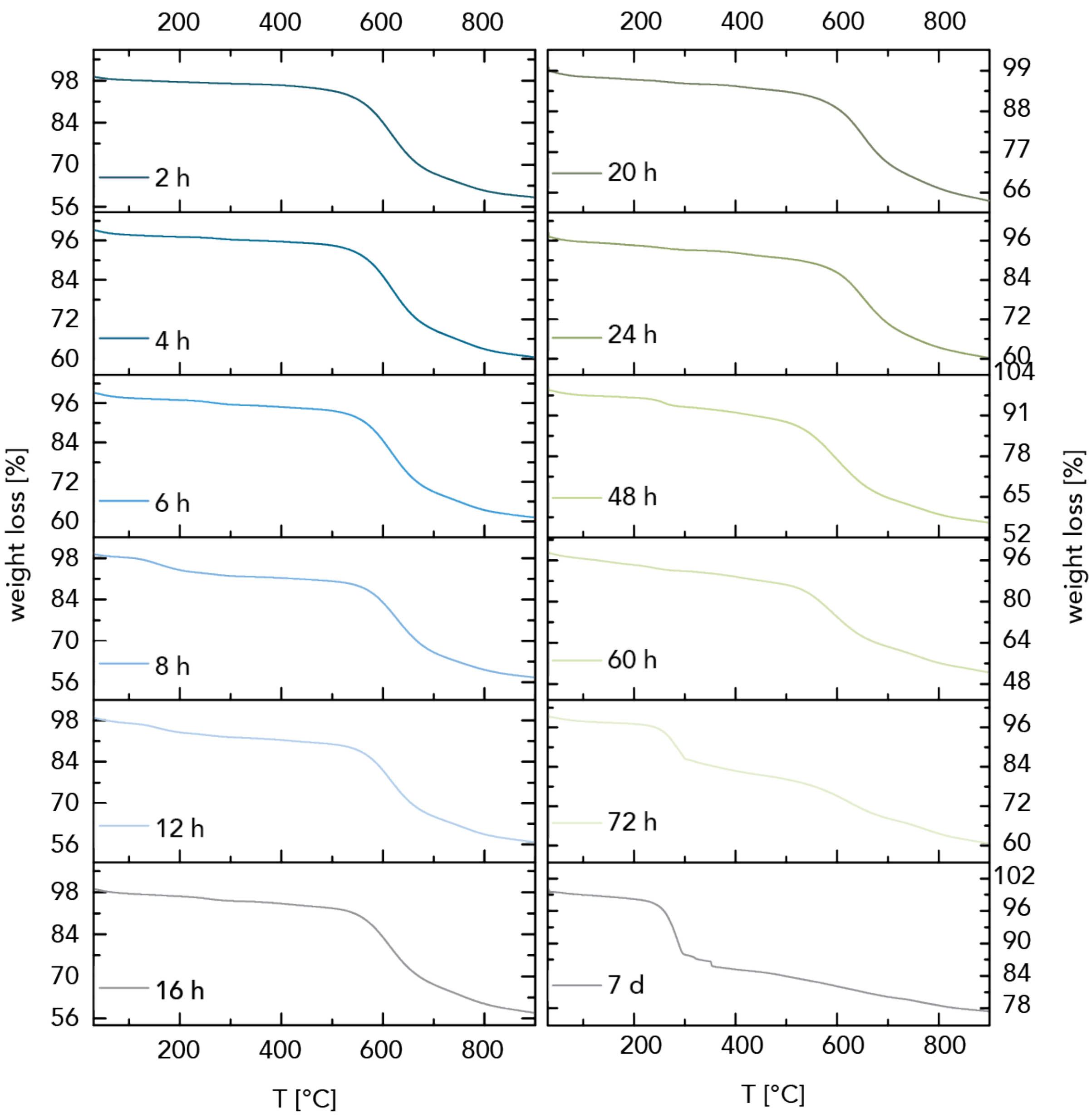

Figure S 12 TGA measurements of all samples synthesized at 250°C without HOAc. At $t_r \geq 72$ h a significant drop in decomposition *T* is visible.

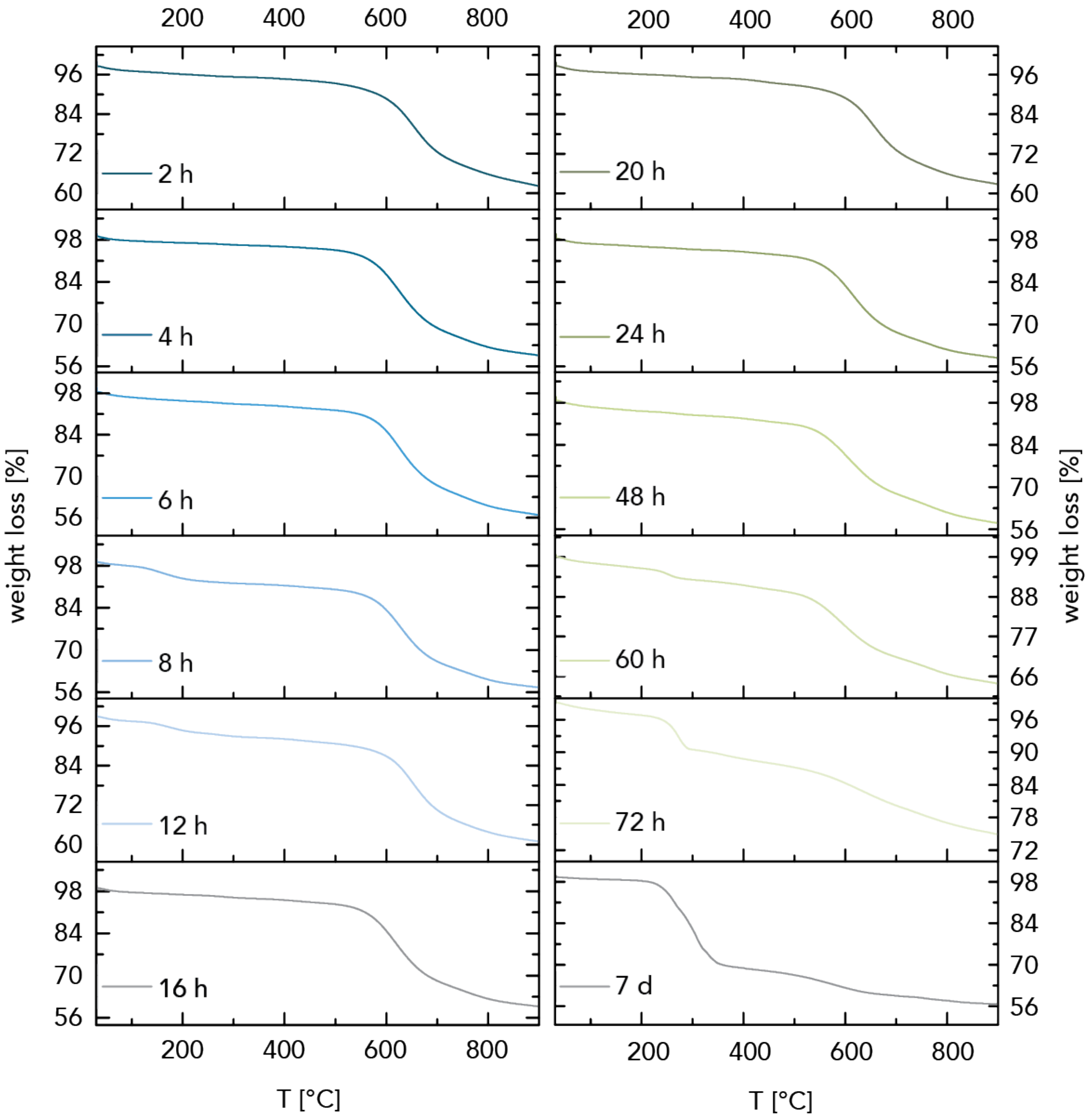

Figure S 13 TGA measurements of all samples synthesized at 250°C in the presence of HOAc. At $t_r \geq 60$ h a significant drop in decomposition *T* is visible-

2.3. Scanning Electron Microscopy

Scanning electron microscopy was carried out with a Quanta 200F FEI microscope. Typically, the samples were measured at 5 kV, with a working distance of 9 mm and spot size 2.0. Prior to imaging, samples were loaded onto carbon-tape on steel sample holders and coated by sputtering with a 17 nm thick layer of Au/Pd 60/40 alloy with a Quarum Q105T S sample preparation system. The 437 SEM images used for both the expert interpretation and the machine-learning supported morphology analysis are provided as an open access dataset with doi 10.5281/zenodo.4544904.

2.3.1. SEM Monowave Synthesis of PI(TAPB-PMA)

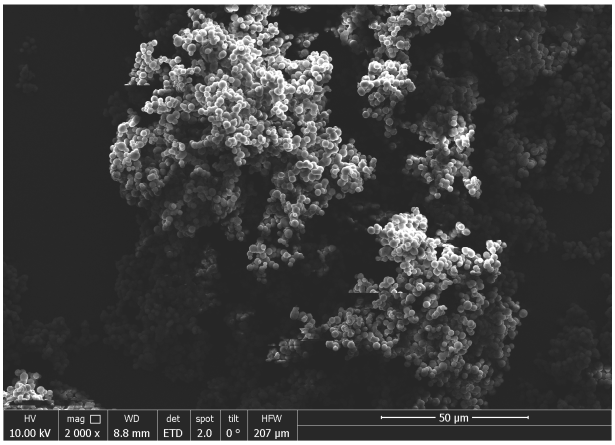

Figure S 14 SEM image of PI(TAPB-PMA) synthesized at T_r = 200 °C and t_r = 4 h in the Anton Paar monowave reactor, batch number 1

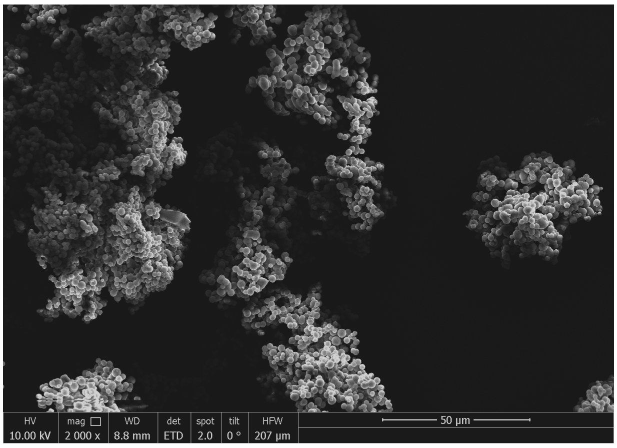

Figure S 15 SEM image of PI(TAPB-PMA) synthesized at T_r = 200 °C and t_r = 4 h in the Anton Paar monowave reactor, batch number 2

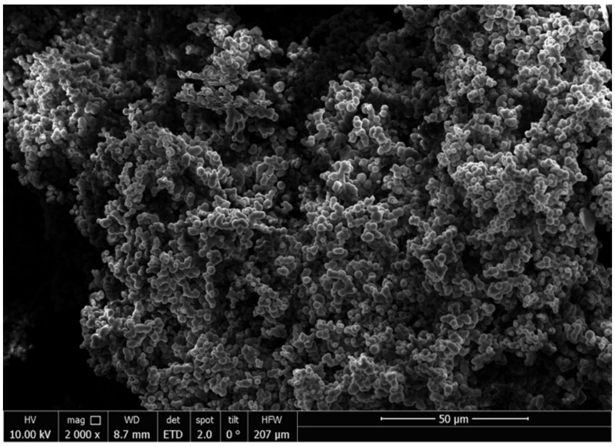

Figure S 16 SEM image of PI(TAPB-PMA) synthesized at T_r = 200 °C and t_r = 4 h in the Anton Paar monowave reactor, batch number 3.

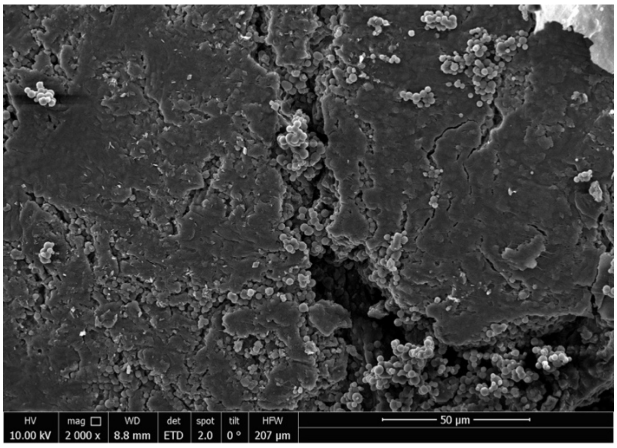

Figure S 17 SEM image of PI(TAPB-PMA) synthesized at T_r = 200 °C and t_r = 4 h in the Anton Paar monowave reactor, batch number 4.

2.3.2. SEM Multiwave upscale synthesis PI(TAPB-PMA)

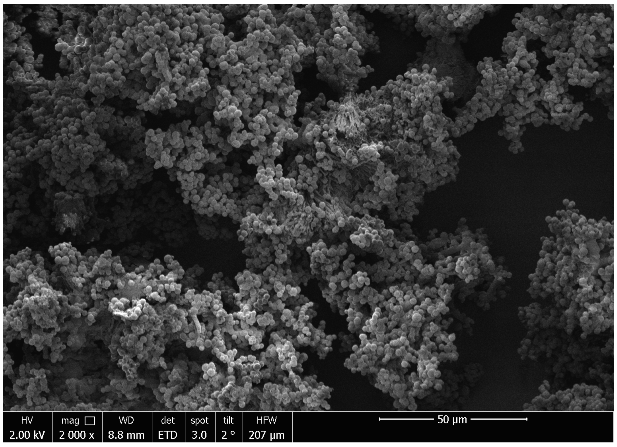

Figure S 18 SEM image of PI(TAPB-PMA) synthesized at $T_r = 250$ °C and $t_r = 4$ h in the Anton Paar multiwave reactor, batch number 1.

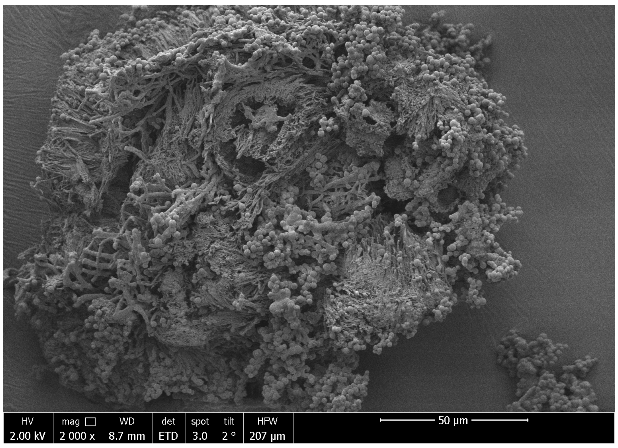

Figure S 19 SEM image of PI(TAPB-PMA) synthesized at $T_r = 250$ °C and $t_r = 4$ h in the Anton Paar multwave reactor, batch number 2.

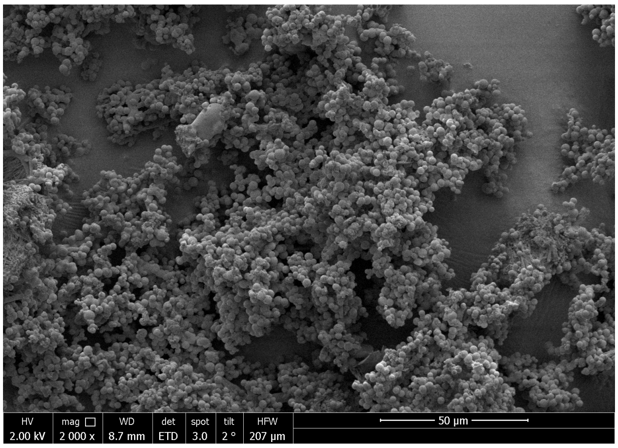

Figure S 20 SEM image of PI(TAPB-PMA) synthesized at $T_r = 250$ °C and $t_r = 4$ h in the Anton Paar multiwave reactor, batch number 3.

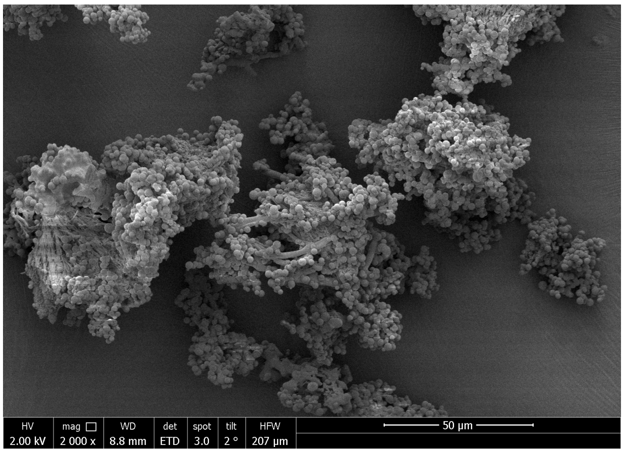

Figure S 21 SEM image of PI(TAPB-PMA) synthesized at $T_r = 250$ °C and $t_r = 4$ h in the Anton Paar multiwave reactor, batch number 4.

2.3.3. SEM solid-state polymerization

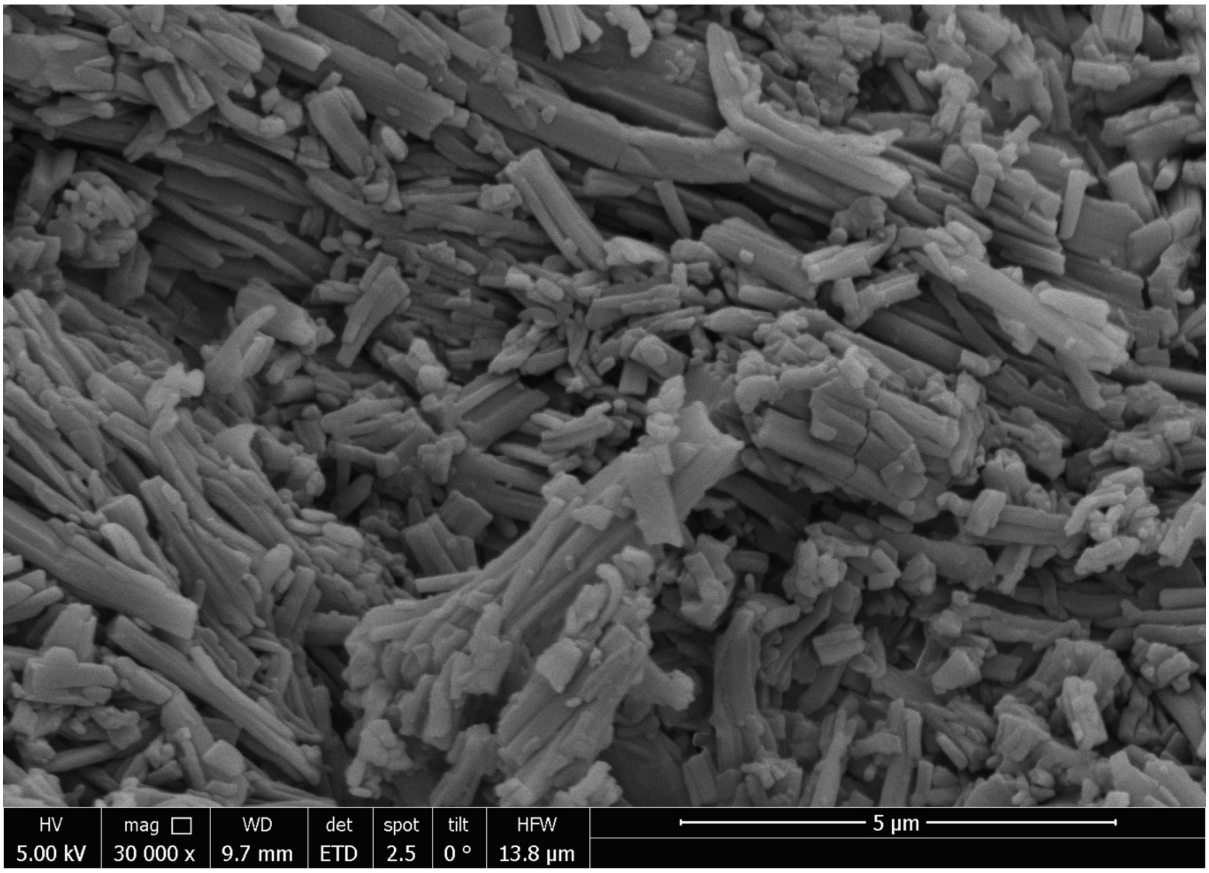

Figure S 22 SEM image of PI(TAPB-PMA) synthesized from solid state polymerization.

2.4. Low pressure gas sorption measurements

Low pressure CO_2 physisorption isotherms were measured volumetrically at 195 K and 273 K up to 1 bar using an Autosorb-IQ-MP from Quantachrome equipped with a Quantachrome CryoCooler for temperature regulation. Isotherm points chosen to calculate the BET surface area were subject to the consistency criteria detailed by Rouquerol.[1]The pore size distribution was derived from the adsorption isotherms at

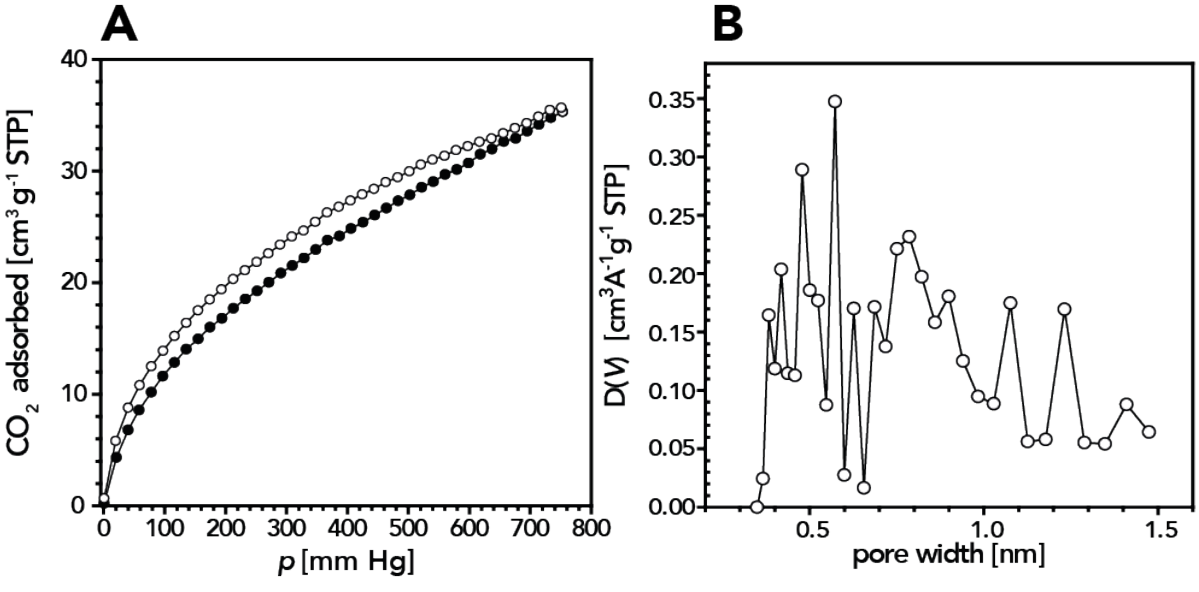

Figure S 23 A CO_2 absorption 273 K, **B** PSD D(V) at 273 K, of PI(TAPB-PMA) synthesized at t_r = 24 h and T_r = 200 °C.

273 K using the Monte Carlo model for carbon adsorbents. In all adsorption plots, open circles indicate the adsorption branch and filled circles indicate the desorption branch.

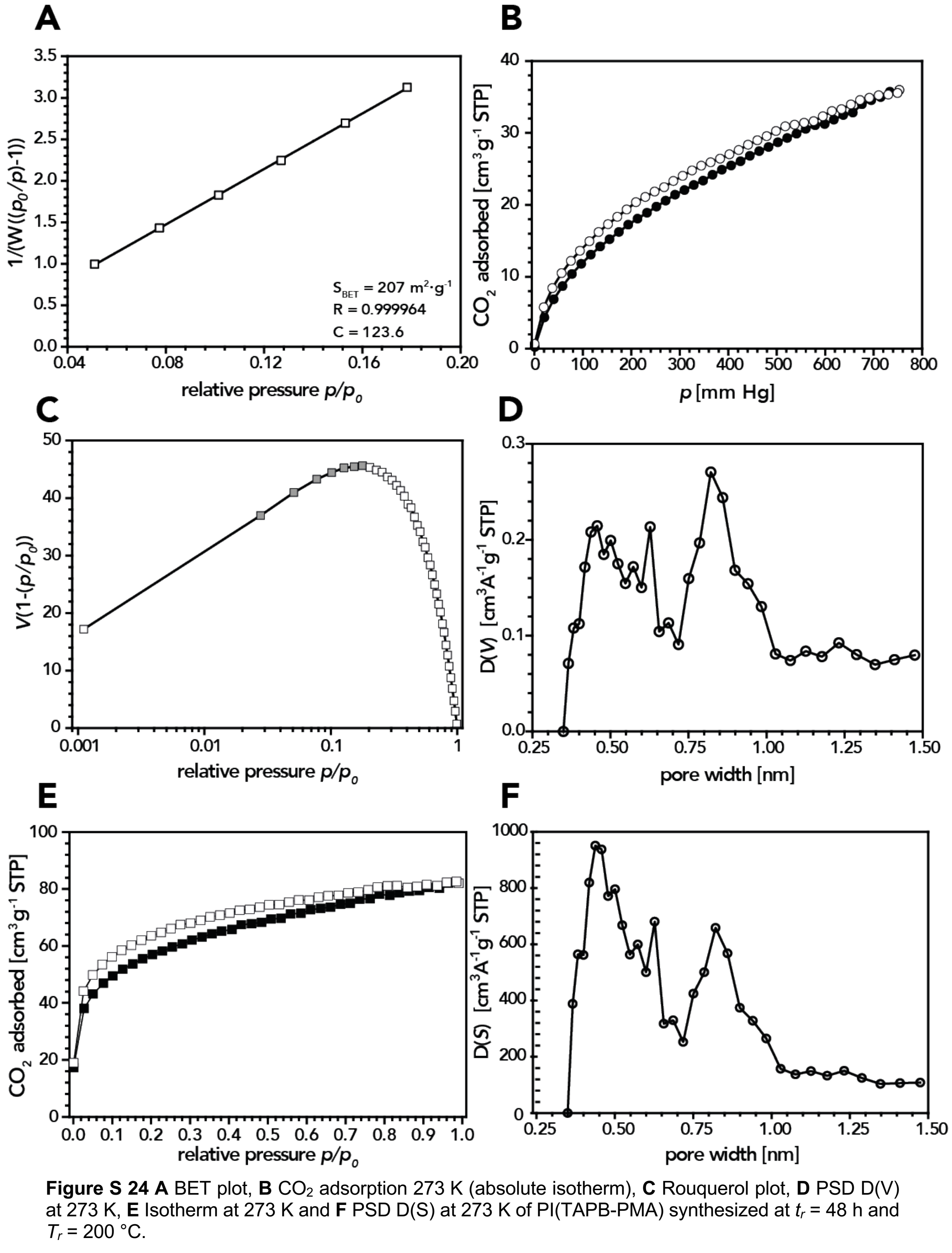

Figure S 24 A BET plot, **B** CO_2 adsorption 273 K (absolute isotherm), **C** Rouquerol plot, **D** PSD D(V) at 273 K, **E** Isotherm at 273 K and **F** PSD D(S) at 273 K of PI(TAPB-PMA) synthesized at t_r = 48 h and T_r = 200 °C.

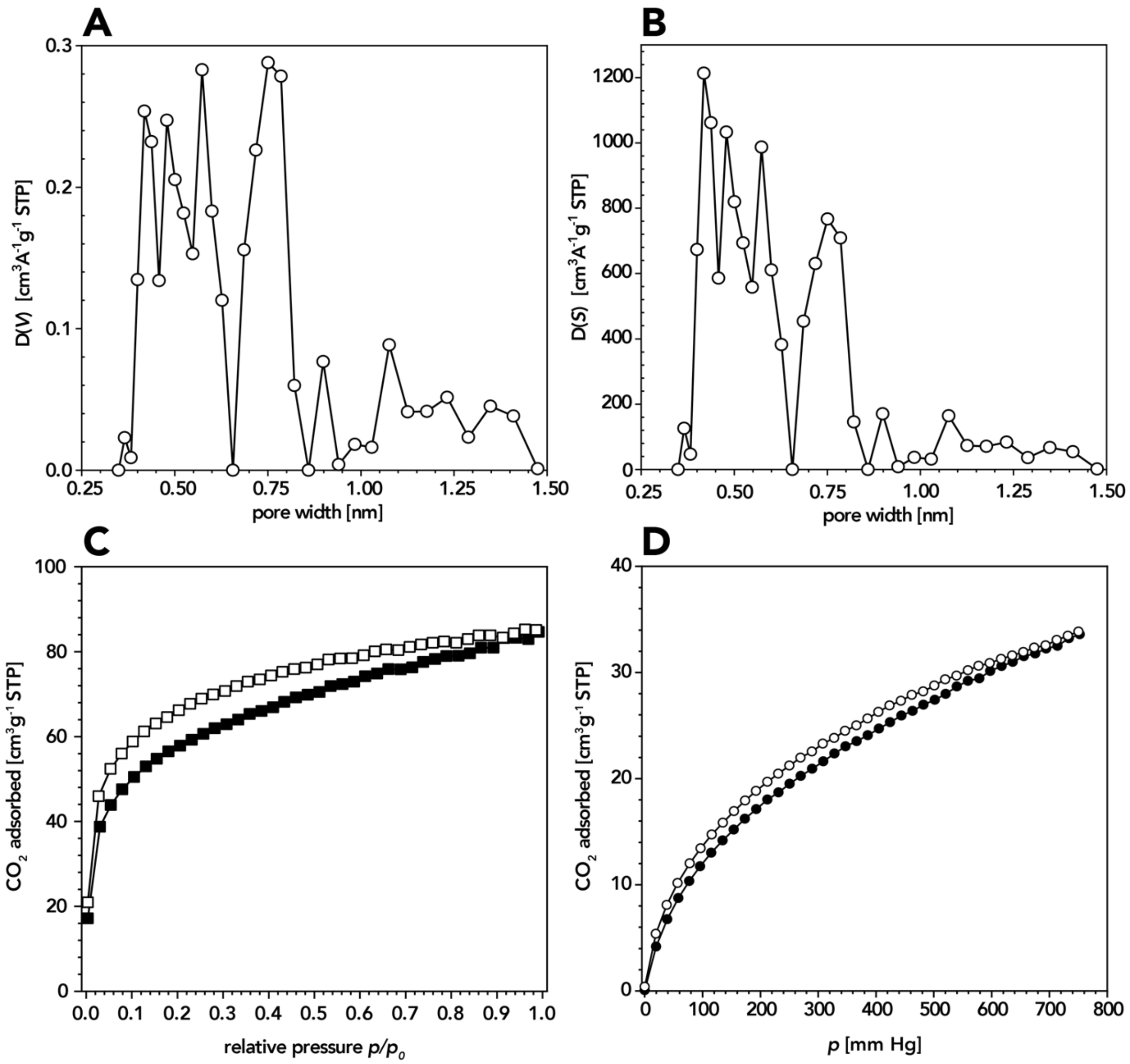

Figure S 25 A PSD D(S) at 273 K, **B** PSD D(V) at 273 K **C** Isotherm at 195 K, **D** CO_2 adsorbed at 273 K (absolute isotherm), of PI(TAPB-PMA) synthesized at t_r = 60 h and T_r = 200 °C.

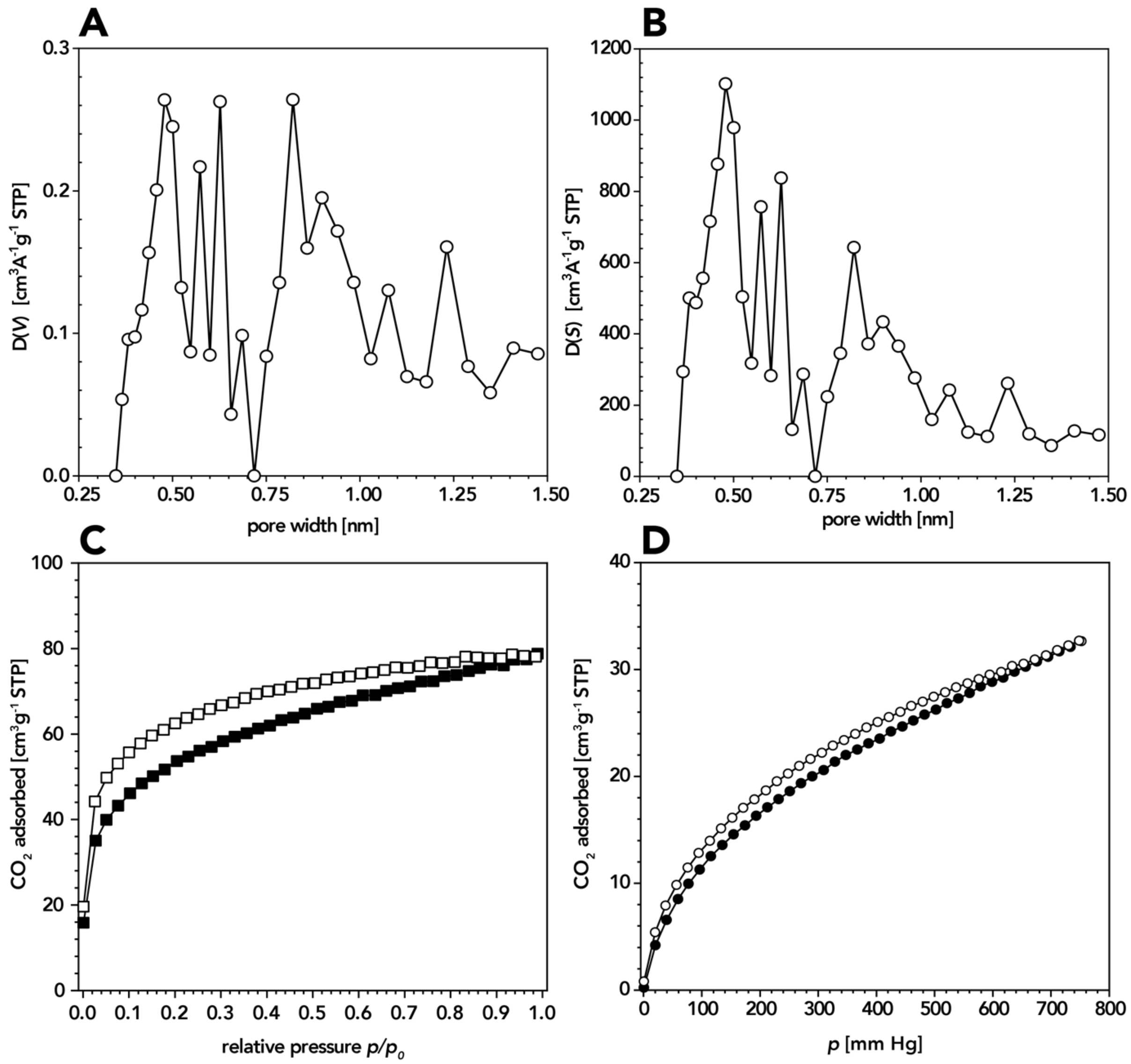

Figure S 26 A PSD D(S) at 273 K, **B** PSD D(V) at 273 K **C** Isotherm at 195 K, **D** CO_2 adsorbed at 273 K (absolute isotherm), of PI(TAPB-PMA) synthesized at t_r = 72 h and T_r = 200 °C.

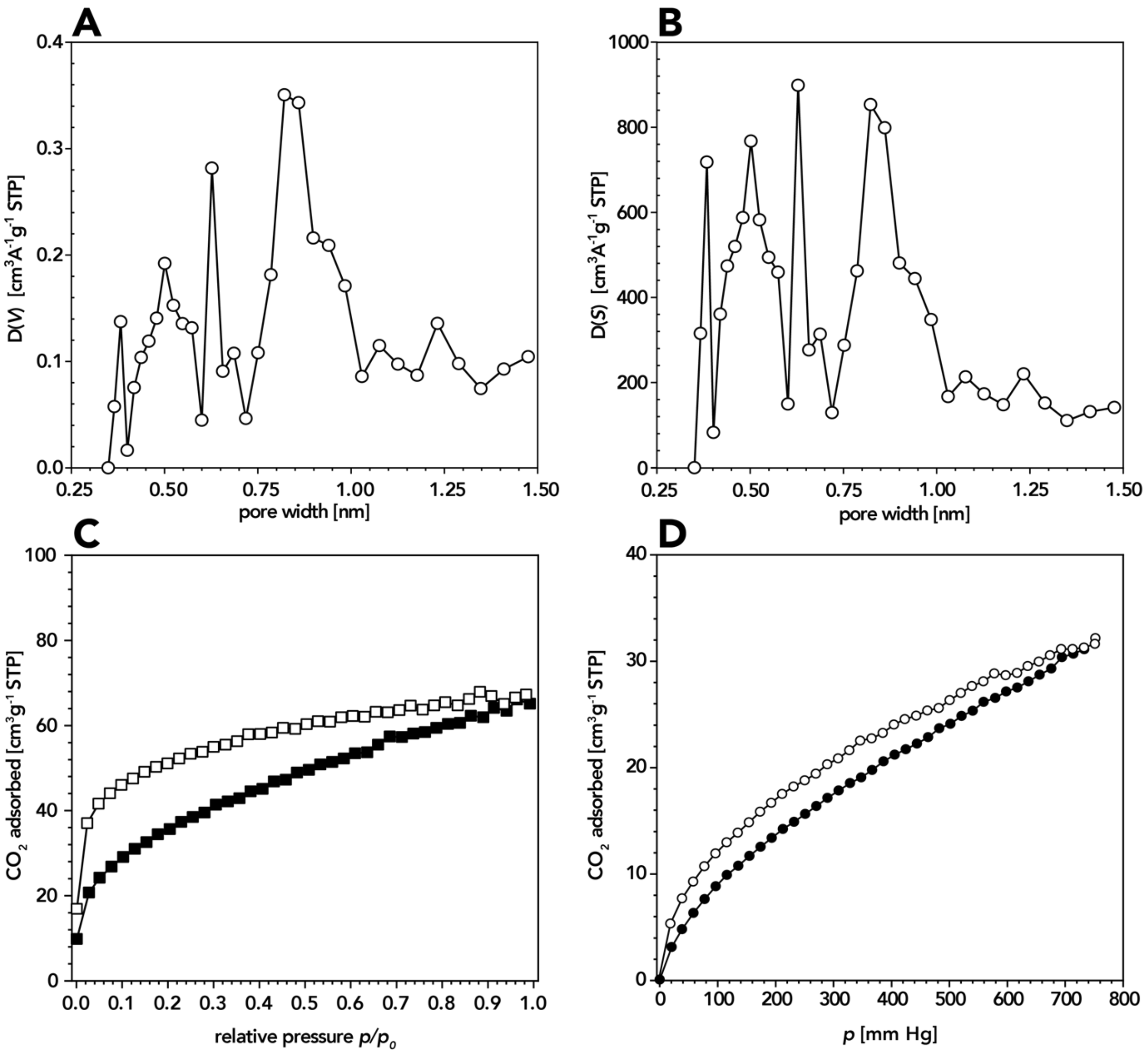

Figure S 27 A PSD D(S) at 273 K, **B** PSD D(V) at 273 K **C** Isotherm at 195 K, **D** CO_2 adsorbed at 273 K, of PI(TAPB-PMA) synthesized at t_r = 168 h and T_r = 200 °C.

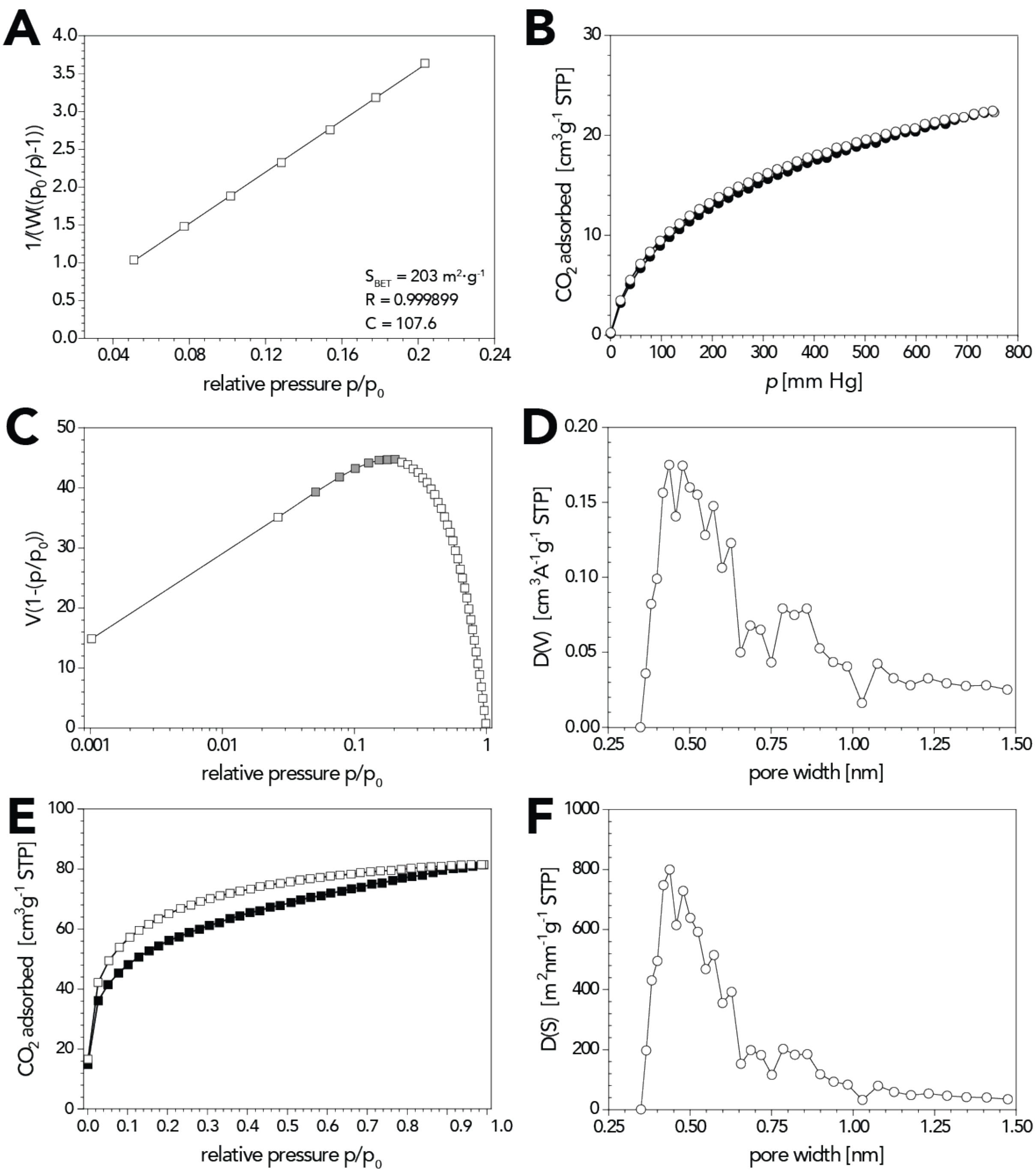

Figure S 28 A BET plot, **B** CO_2 adsorption 273 K (absolute isotherm), **C** Rouquerol plot, **D** PSD D(V) at 273 K, **E** Isotherm (195K) and **F** PSD D(S) at 273 K of PI-pellet.

2.5. Hg- Porosimetry

The pore size distribution of sample ML129B (PI(TAPB-PMA), t_r = 24 h and T_r = 200 °C) was determined by mercury intrusion porosimetry (PASCAL 140/440, Thermo Fisher Scientifc), assuming a contact angle of 140°. The maximum intrusion pressure was 400 MPa, corresponding to a minimum accessible pore opening diameter of 4 nm.

Density and pore characteristics of the warm-pressed PI(TAPB-PMA) pellet were characterized by mercury intrusion porosimetry [*same testing conditions as other sample*]. Owing to an apparent compression of the sample at intrusion pressures above 100 MPa, a compressibility correction was applied to the intrusion curve.
Furthermore, the flexural strength of the warm-pressed PI(TAPB-PMA) material was evaluated using a three-point flexural test setup, following EN ISO 178. Test specimens with dimensions of 5 x 1.6 x 30 mm were obtained using a diamond cutting disc. A total of three specimens were tested until fracture using a crosshead speed of 1 mm min^{-1} (Universal testing machine Model 1474, Zwick, Germany).

2.6. Powder X- Ray Diffractometry

A PANalyticalX'Pert Pro multi-purpose diffractometer (MPD) in Bragg Brentano geometry operating with a Cu anode at 45 kV, 40 mA and an X-Celerator multichannel detector was used. Samples were in most cases ground and mounted as loose powders on silicon single crystal sample holders, in other cases the spongy structure had to be fixed to the sample holder with heptane/Vaseline solution. The diffraction patterns were recorded between 1 and 30° (2θ) with 74.970 s/step and a step size of 0.0201°, sample holders where rotated during the measurement with 4 s/turn.

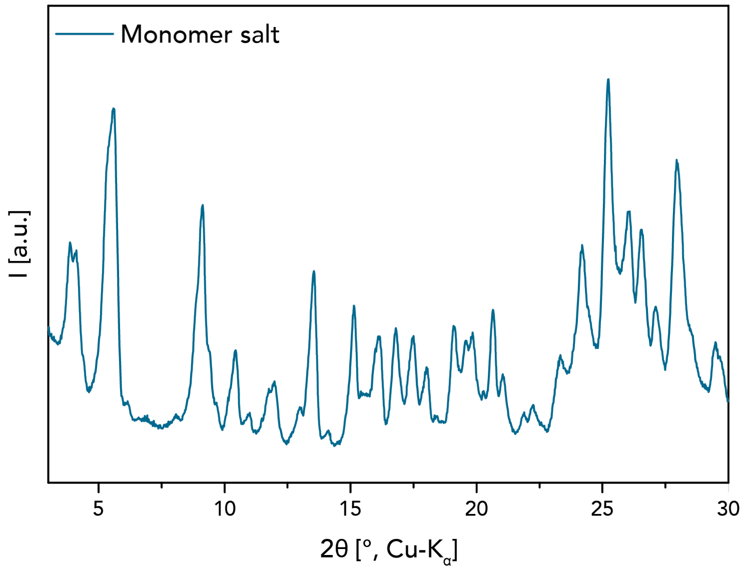

Figure S 30 PXRD pattern of the monomer salt [(H_3TAPB^{3+})$_2$(PMA^{2-})$_3$]

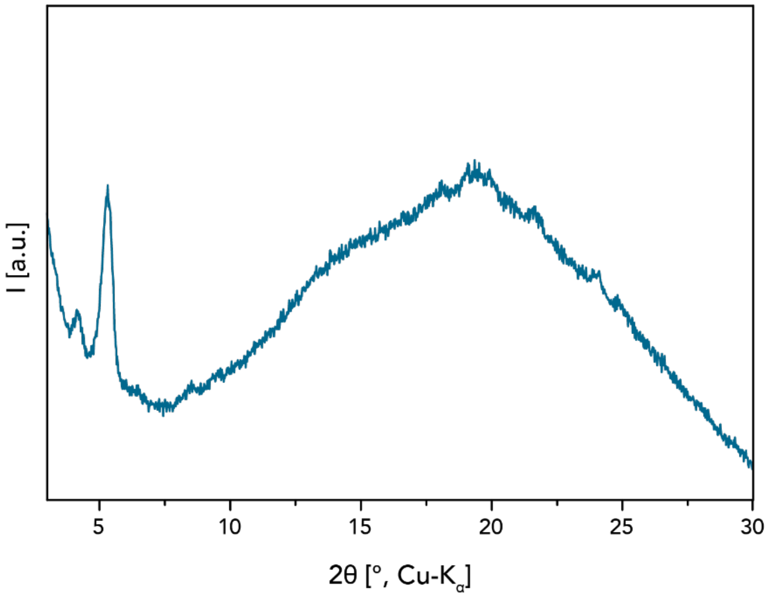

Figure S 29 Figure PXRD of the solid-state polymerized PI(TAPB-PMA). The two rather sharp reflexes around 5° 2θ could be a sign that monomer salt is still present.

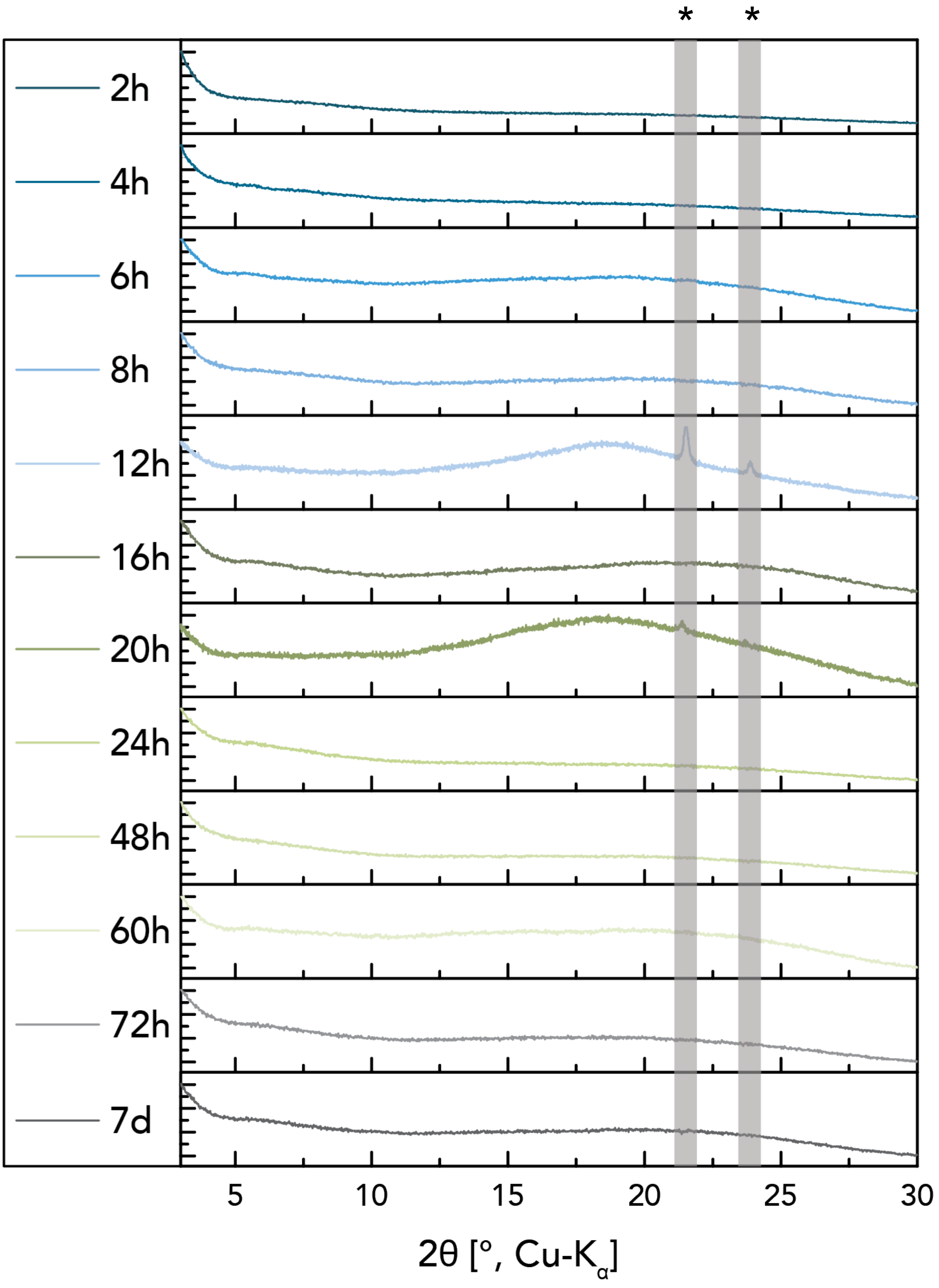

Figure S 31 XRD diffractogramms of all sampels synthesized at 200 °C without HOAc. Reflections marked with asterisks (in gray boxes) arise from vaseline used to prepare the samples.

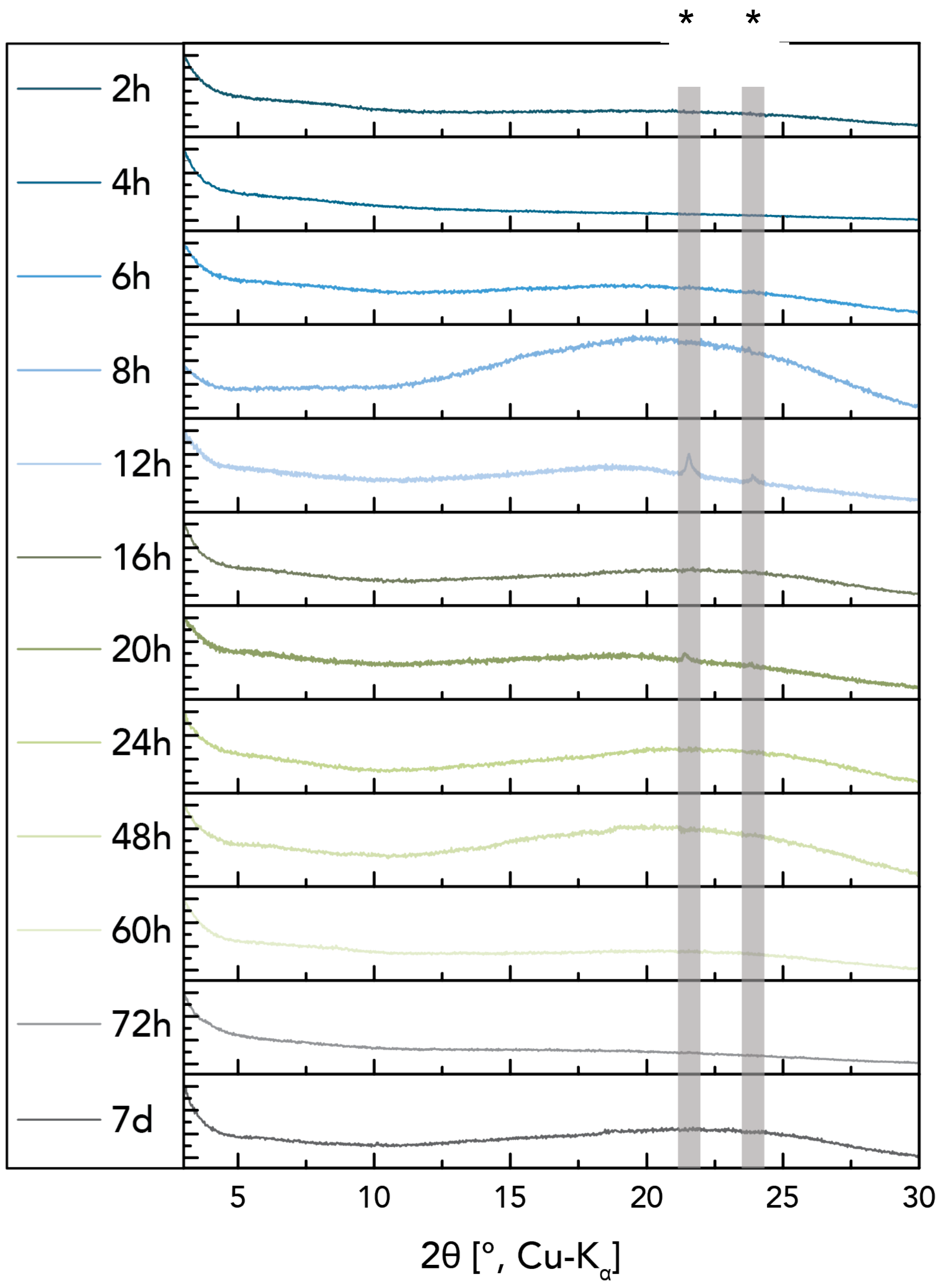

Figure S 32 XRD diffractogramms of all sampels synthesized at 200 °C in the presende of HOAc. Reflections marked with asterisks (in gray boxes) arise from vaseline used to prepare the samples.

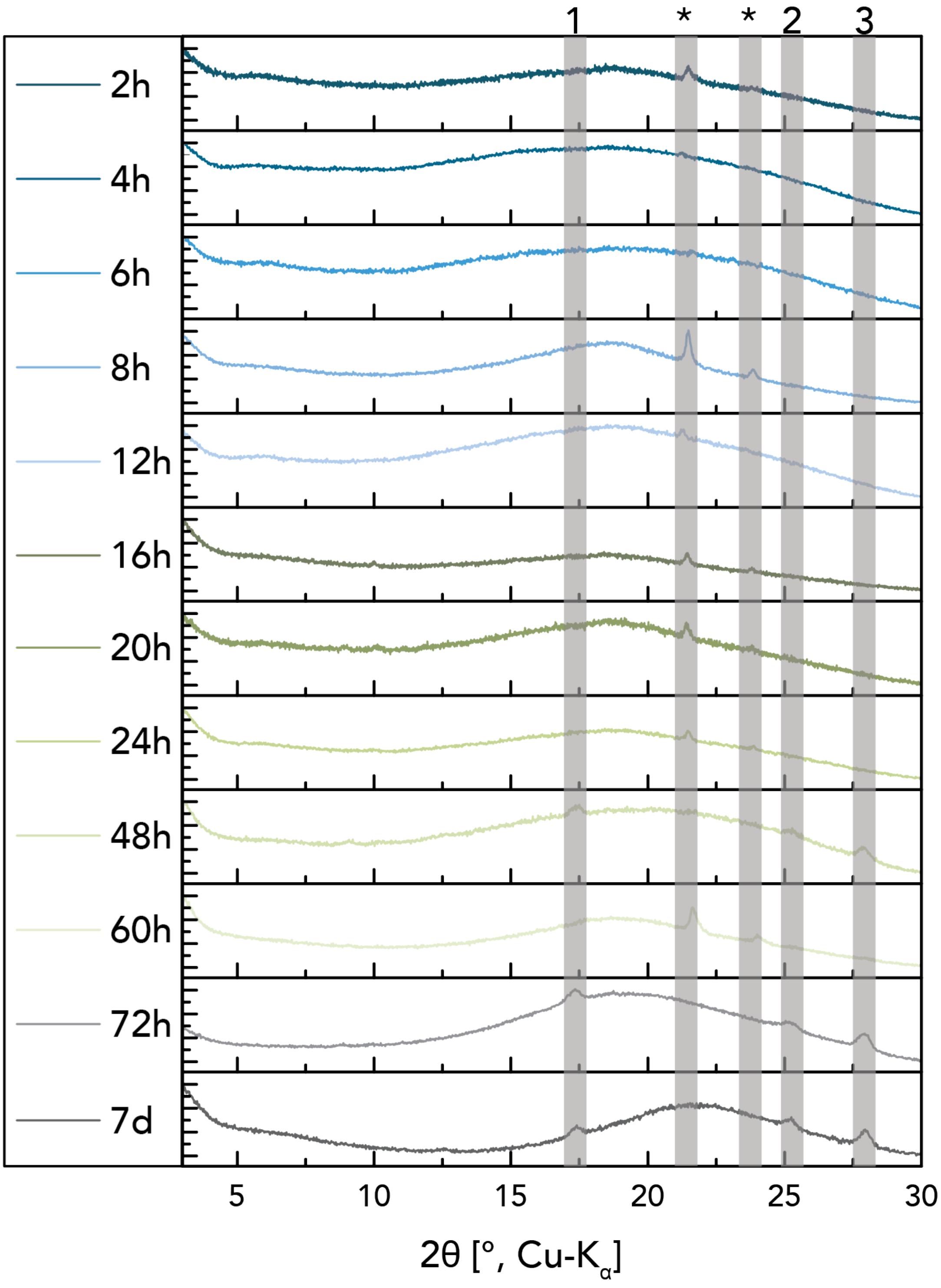

Figure S 33 XRD diffractogramms of all sampels synthesized at 250 °C without HOAc. Reflections marked with asterisks arise from vaseline used to prepare the samples. **1, 2,** and **3** arise additionally at t_r > 48 h and are interpreted as the reflexes stemming from a PI/Silicate hybrid.

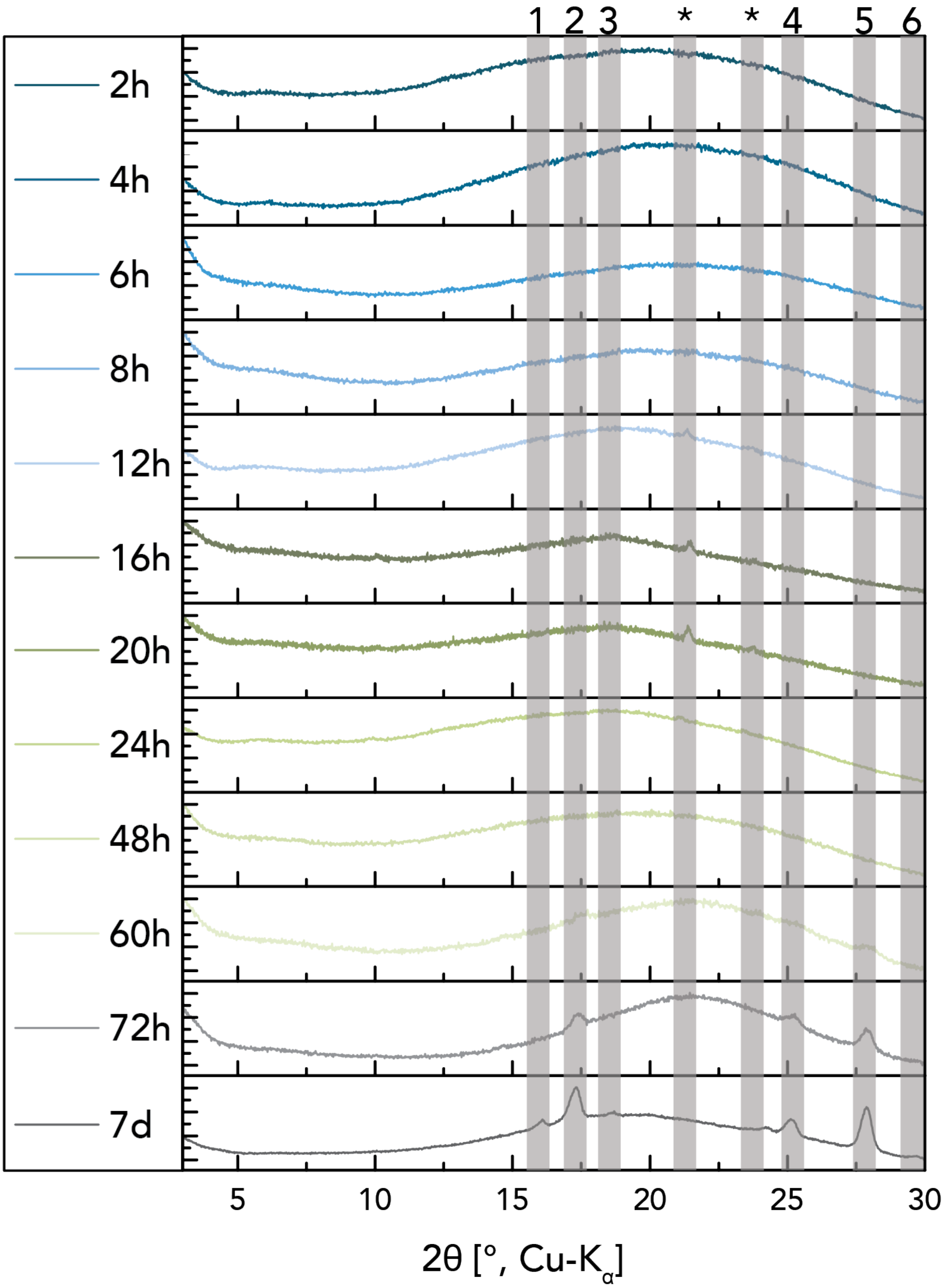

Figure S 34 XRD diffractogramms of all sampels synthesized at 250 °C in the presence of HOAc. Reflections marked with asterisks arise from vaseline used to prepare the samples. **1, 2, 3, 4, 5,** and **6** arise additionally at t_r > 48 h and are interpreted as the reflexes stemming from a PI/Silicate hybrid.

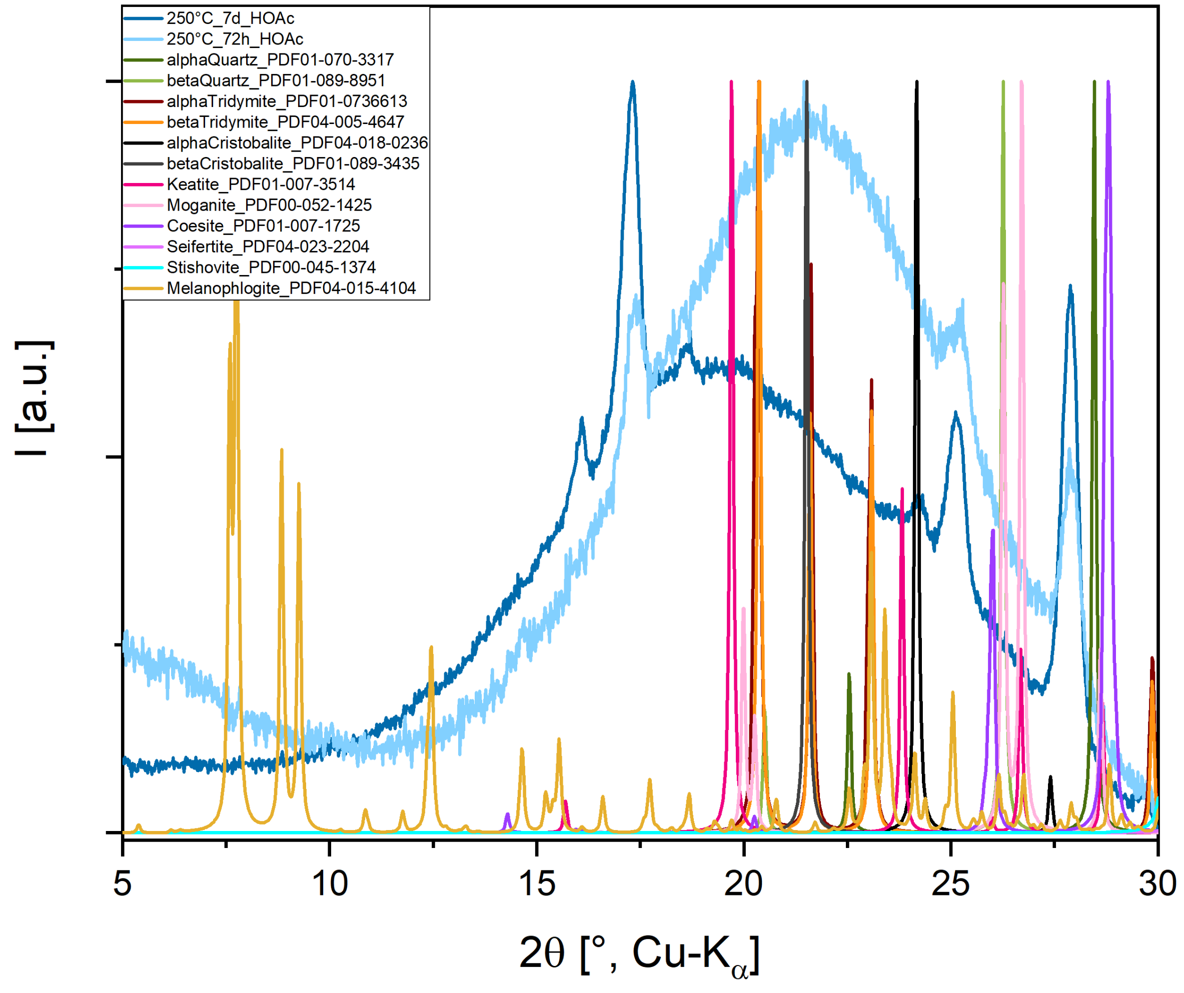

Figure S 35 Overaly of diffractograms of the different modifications of SiO_2 (PDF numbers can be found in the legend) and two of the samples showing reflections (250 °C and t_r=72 and 168 h). No matching patterns were observed; hence the formation of Si-hybrids is suggested.

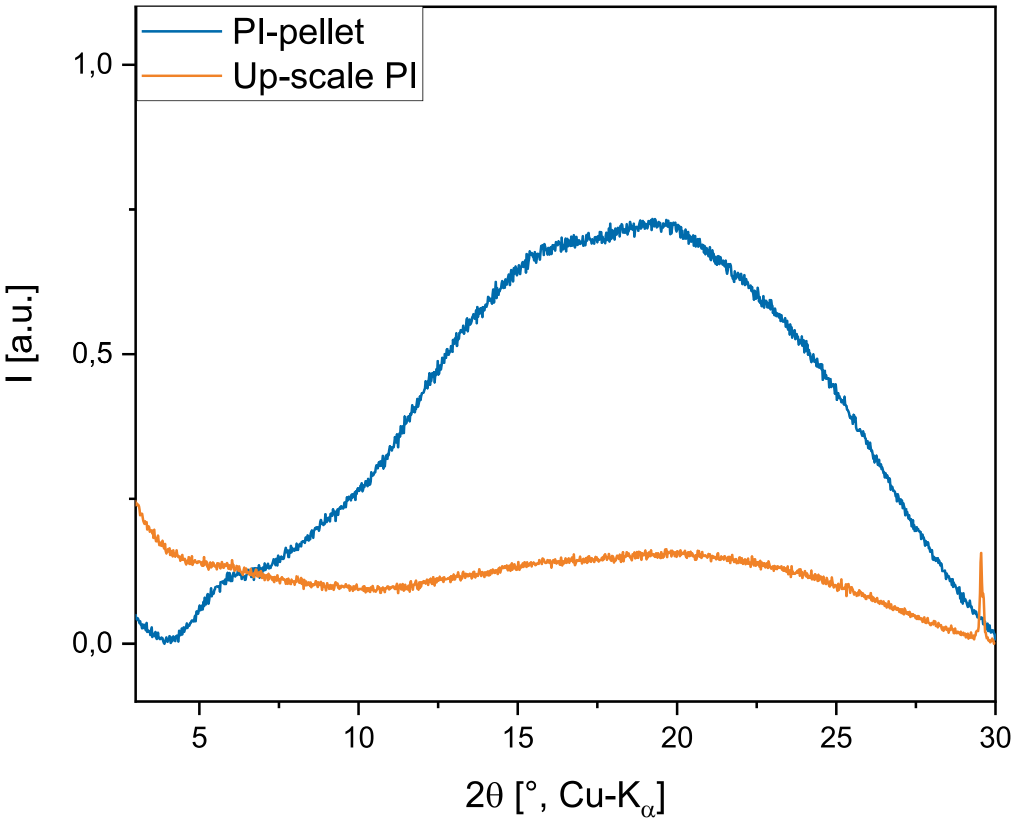

Figure S 36 XRD diffractogram of the microwave upscaled PI powder (orange curve) and after warm pressing the powder the resulting PI-pellet (blue curve).

2.7. Differential Scanning Calorimetry DSC

DSC measurements were performed on a DSC832 Mettler Toledo machine with a heating rate of 5 K/min and a nitrogen gas flow of 10 µl/min. The temperature ranged measured was 30-400 °C.

2.8. Literature Comparison

Table S2 comparison of the characterization results of the differently synthesized PI(TAPB-PMA) in literature.

	**Macromol. 2013**[2]	**Soft Matter 2011**[3]	**Micro/Mesop. Mat. 2017**[4]	**Nature Comm. 2014**[5]	**PI(TAPB-PMA)**
**Protocol**	*m*-cresol, 220 °C, 30 h	*m*-cresol, 200 °C, 32 h	H_2O 1 h, ss 300 °C 4 h	NMP, mesitylene, 250 °C, 7 d	H_2O
**Additives**	isoquinoline	isoquinoline	Pluronic F-127	isoquinoline	HOAc
**Crystallinity**	amorphous	amorphous	amorphous	crystalline	amorphous
**T_d [°C]**	tangent method > 530	5% 530	tangent method 590	535	up to 595
**CO_2 uptake**	9.9%	-	3.2%	-	0.4%
**SA_{BET} [m^2 g^{-1}]**	586 [N_2]	570 [N_2]	-	1297 [N_2]	207 [CO_2]
**PSD [nm]**	max at 0,5	0,6/0,7/0,9	-	3,7/ 2,0	< 0,8/< 50/0,1-2 µm/1-100 µm
**Total pore V [cm^3 g^{-1}]**	0.31	-	-	0.67	7.25
**Morphologies SEM**	irregular	irregular	spherical	irregular	Monodisperse spheres/fibers
**TEM**	Uniform pores	-	-	-	-
**Applications**	Air/water treatment	-	catalysis	T sensing	Water treatment

2.9. Automated Image Analysis

Image analysis was performed in Python (v. 3.x) using the open-source software library Keras (v. 2.4.3) as an interface for artificial neural networks. In a preliminary step images were split in an ordinary grid into 20 tiles. Each individual tile was then analysed whether it contains useful information (foreground). This was performed by first calculating a pixel foreground threshold using the Otsu method and then removing tiles with less than 10% foreground pixels.

Next, we used the tiled images as input for six different artificial neural networks: Xception, VGG16, VGG19, InceptionV3, MobileNet and ResNet. For all six neural networks the prediction layer has been removed, such that the final output of each network is a pseudo feature string varying between 512 and 2048 dimensions. All six neural networks have been pre-trained on regular images using the imagenet dataset. The performance of individual network architectures as well as different feature normalization methods were evaluated by calculating the Cohen's d (effect size) between the eucledian distances of tiles that belong to the same original image, and tiles that belong to different images.

$$d = \frac{\overline{x}_1 - \overline{x}_2}{\sqrt{\frac{s_1^3 + s_2^3}{2}}}$$

Next, we calculated the centroid (mean) point within the 20 tiles of an image, such that each SEM image corresponds to exactly one point. To further analyse and visualize our data, we applied dimensionality reduction techniques to our dataset to reduce the number of dimensions to two. Both principal component analysis (PCA) and t-stochastic neighborhood embedding (t-SNE) have been performed using the python package scikit-learn (v. 0.2.4).

To use a homogenous set of images with a similar range of magnification we decided to include only images between 1300x and 4000x which correspond to ca. half of all images. Next, we identified the ideal number of clusters using the k-Means clustering and the silhouette method. K-Means clustering partitions n datapoints, e.g., images, into k clusters in which each point belongs to the cluster with the nearest mean. The silhouette method calculates how similar objects are to their own cluster compared to other clusters. Values close to one indicate perfect cohesion in individual clusters, whereas values close to zero indicate bad clustering.

A Comparison between different NN topologies

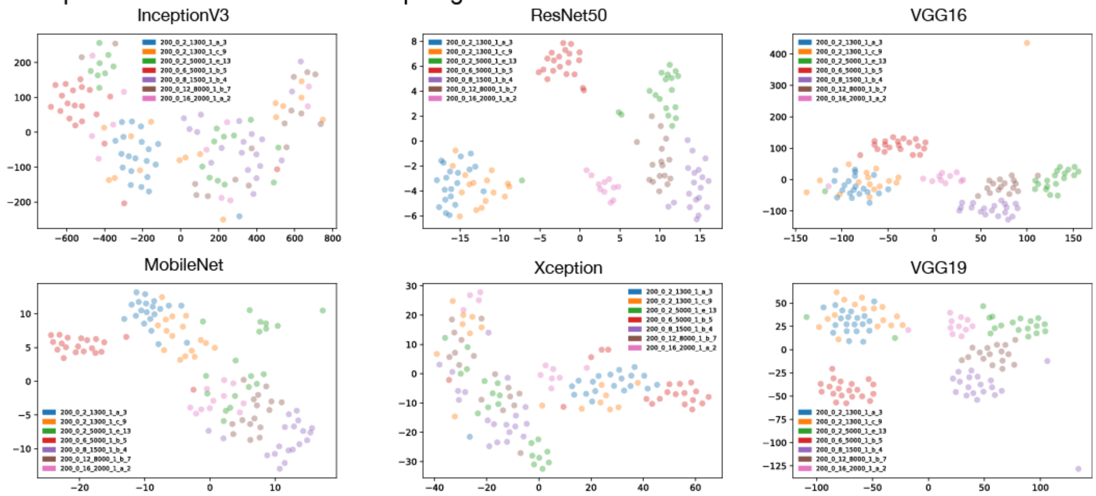

B Impact of magnification (TSNE)

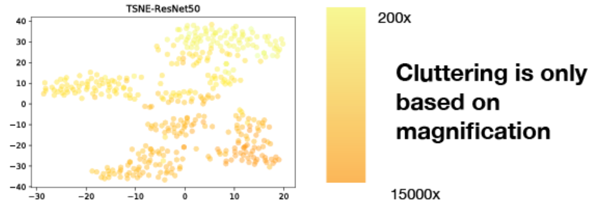

C Example images for the 6 clusters (Perplexity12)

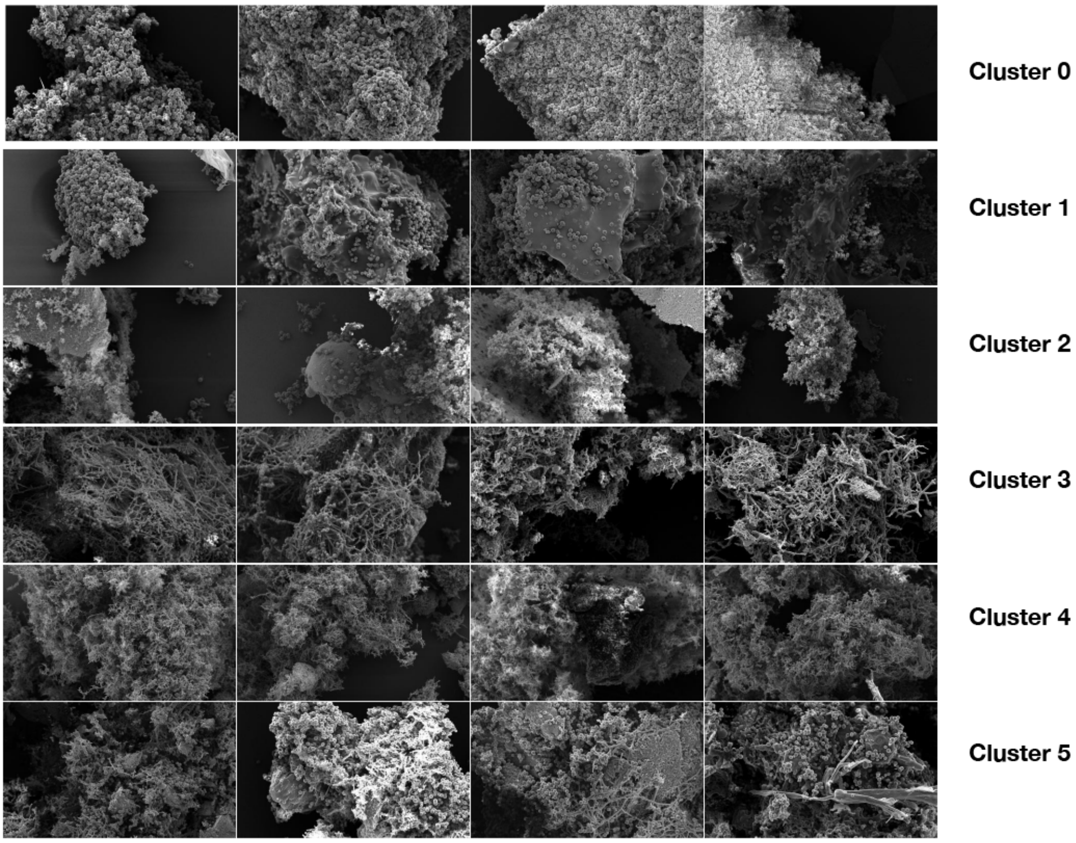

D TSNE overlay with features

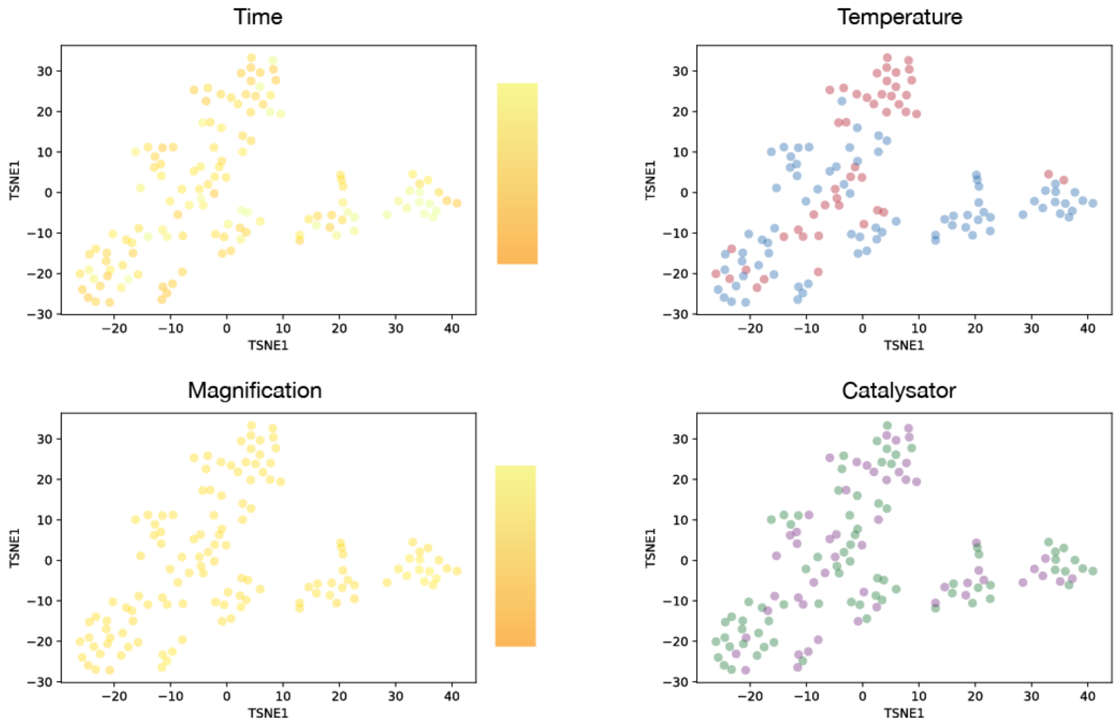

E Cluster and features (absolute number of crystal)

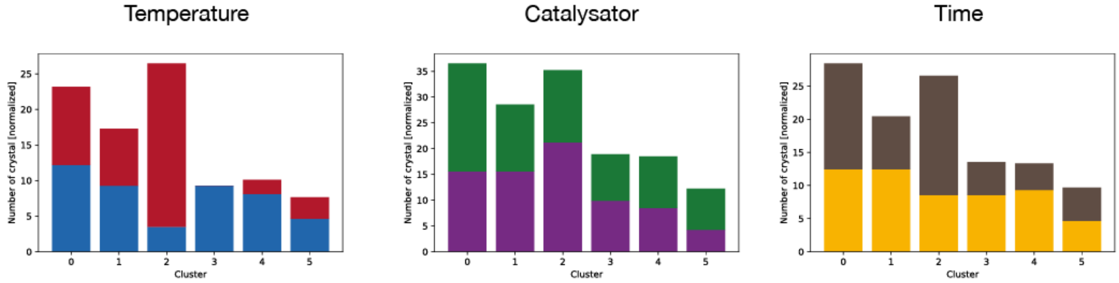

Figure S 37 Results of the computational image analysis performed on 412 SEM images

2.10. Energy-dispersive X-ray spectroscopy (EDAX)

Energy-dispersive X-ray spectroscopy was carried out with Quanta 200F FEImicroscope and the Octane Elite Super X-ray detector. The samples were typically measured at 10 kV. with a working distance of 10 mm and spot size 3.0. Prior to imaging, samples were loaded on carbon coated stubs and coated by sputtering with a 17 nm thick layer of Au/Pd 60/40 alloy with a Quarum Q105T S sample preparation system.

72 h, 250 °C, no HOAc

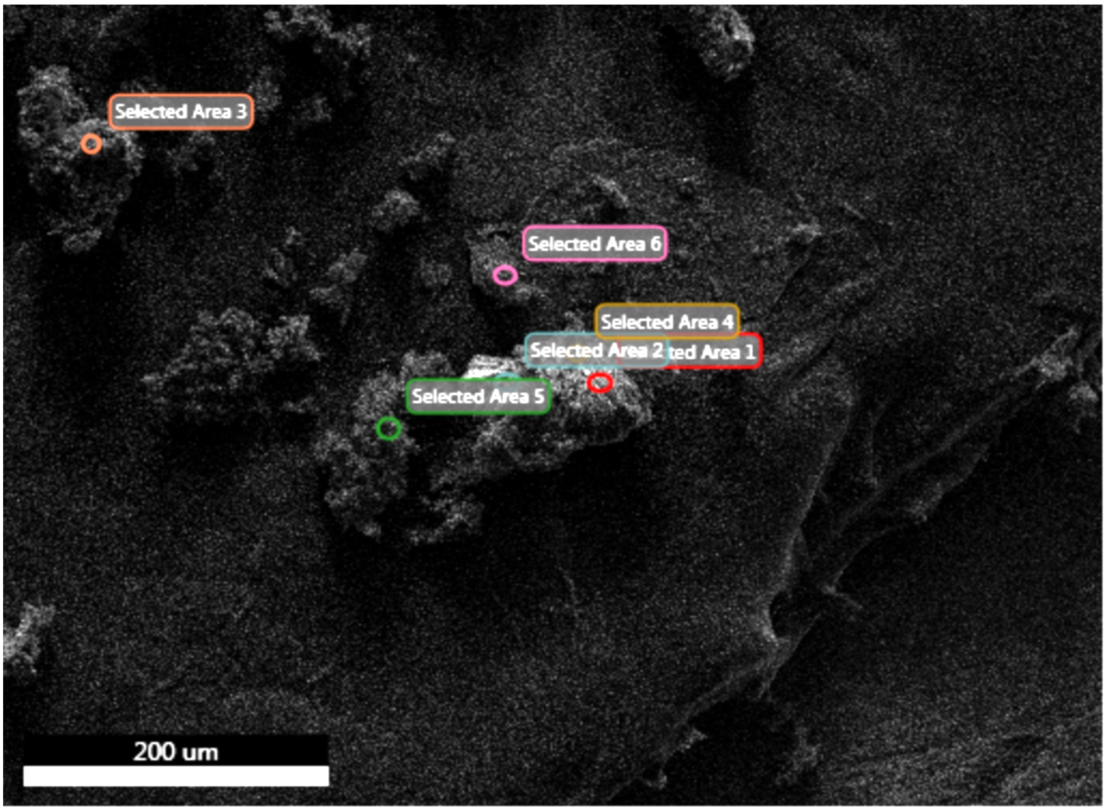

Area 1

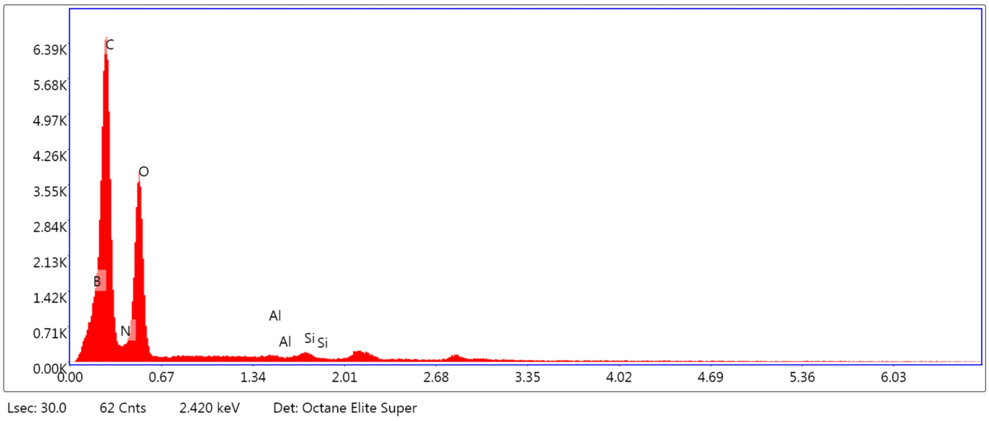

eZAF Smart Quant Results

Element	Weight %	Atomic %	Net Int.	Error %	Kratio	Z	A	F
B K	26.8	30.6	465.9	3.0	0.1902	0.7070	1.0046	1.0000
C K	50.5	52.0	1676.1	5.8	0.2489	0.7408	0.6648	1.0000
N K	1.8	1.6	44.3	23.8	0.0063	0.7234	0.4857	1.0000
O K	19.7	15.2	889.3	6.1	0.0960	0.7089	0.6864	1.0000
AlK	0.4	0.2	18.4	22.9	0.0025	0.6415	1.0018	1.0047
SiK	0.8	0.3	35.8	12.8	0.0051	0.6592	1.0116	1.0058

Area 2

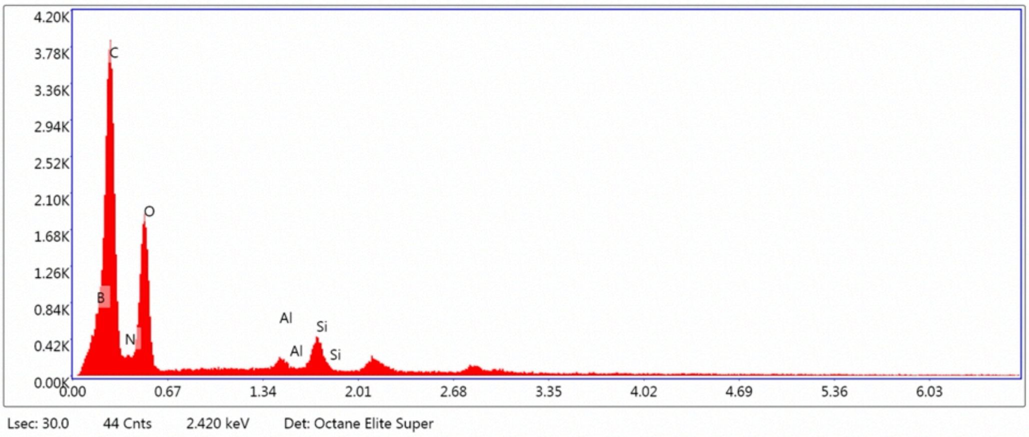

eZAF Smart Quant Results

Element	Weight %	Atomic %	Net Int.	Error %	Kratio	Z	A	F
B K	29.6	34.0	324.3	4.3	0.1876	0.7081	0.8951	1.0000
C K	51.8	53.5	1105.1	6.8	0.2326	0.7419	0.6054	1.0000
N K	0.0	0.0	0.0	100.0	0.0000	0.7244	0.4625	1.0000
O K	12.9	10.0	404.2	8.1	0.0618	0.7099	0.6747	1.0000
AlK	1.5	0.7	50.8	9.8	0.0096	0.6424	1.0071	1.0082
SiK	4.2	1.9	139.5	6.3	0.0284	0.6601	1.0129	1.0042

Area 3

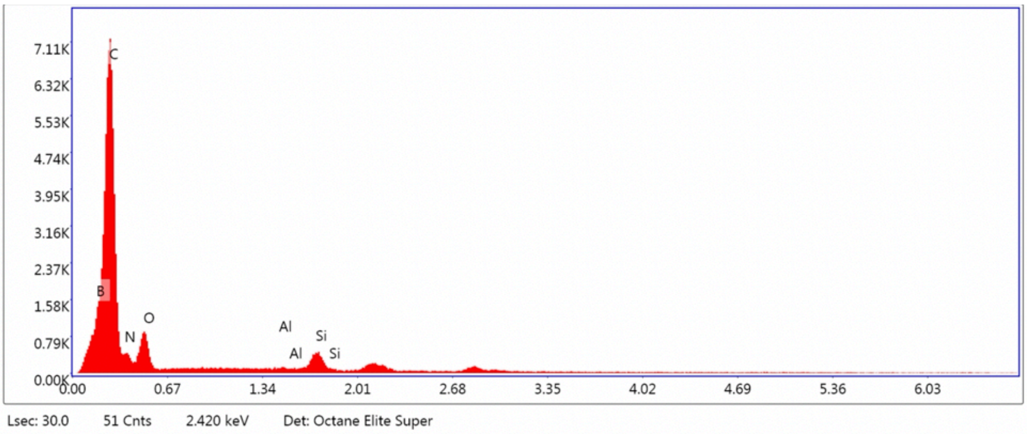

eZAF Smart Quant Results

Element	Weight %	Atomic %	Net Int.	Error %	Kratio	Z	A	F
B K	31.5	34.6	589.2	3.2	0.2296	0.7064	1.0305	1.0000
C K	64.1	63.3	2062.0	6.5	0.2923	0.7404	0.6158	1.0000
N K	0.0	0.0	1.1	100.0	0.0001	0.7232	0.4187	1.0000
O K	0.9	0.7	38.9	45.6	0.0040	0.7088	0.6300	1.0000
AlK	0.3	0.2	17.7	22.2	0.0023	0.6416	1.0143	1.0091
SiK	3.0	1.3	150.1	6.1	0.0206	0.6592	1.0199	1.0056

Area 4

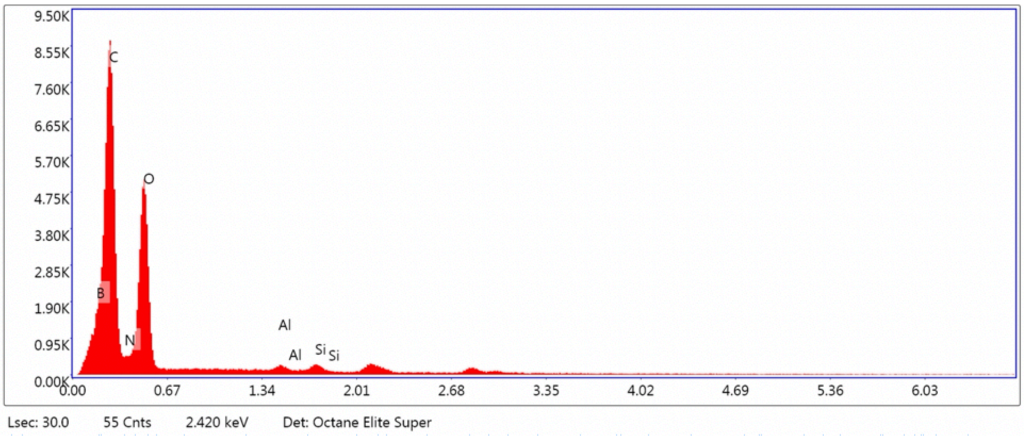

eZAF Smart Quant Results

Element	Weight %	Atomic %	Net Int.	Error %	Kratio	Z	A	F
B K	26.7	30.7	611.5	2.9	0.1846	0.7073	0.9785	1.0000
C K	49.2	51.0	2190.0	5.8	0.2406	0.7410	0.6594	1.0000
N K	2.4	2.1	79.0	17.8	0.0084	0.7236	0.4901	1.0000
O K	19.8	15.4	1211.4	6.0	0.0967	0.7092	0.6872	1.0000
AlK	0.9	0.4	56.5	8.3	0.0056	0.6417	1.0017	1.0048
SiK	1.0	0.5	64.2	8.8	0.0068	0.6593	1.0106	1.0055

Area 5

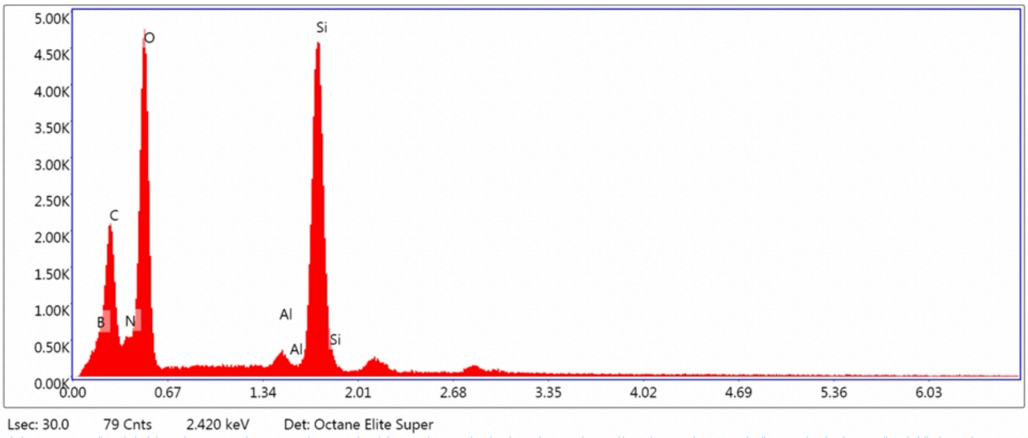

eZAF Smart Quant Results

Element	Weight %	Atomic %	Net Int.	Error %	Kratio	Z	A	F
B K	21.2	30.4	176.2	9.2	0.0667	0.7170	0.4388	1.0000
C K	20.0	25.8	527.2	8.1	0.0726	0.7493	0.4854	1.0000
N K	3.7	4.1	116.2	10.1	0.0154	0.7303	0.5664	1.0000
O K	22.2	21.5	1173.4	5.2	0.1175	0.7148	0.7415	1.0000
AlK	1.8	1.0	94.6	6.3	0.0117	0.6457	1.0014	1.0279
SiK	31.2	17.2	1567.1	2.9	0.2087	0.6637	1.0082	1.0010

Area 6

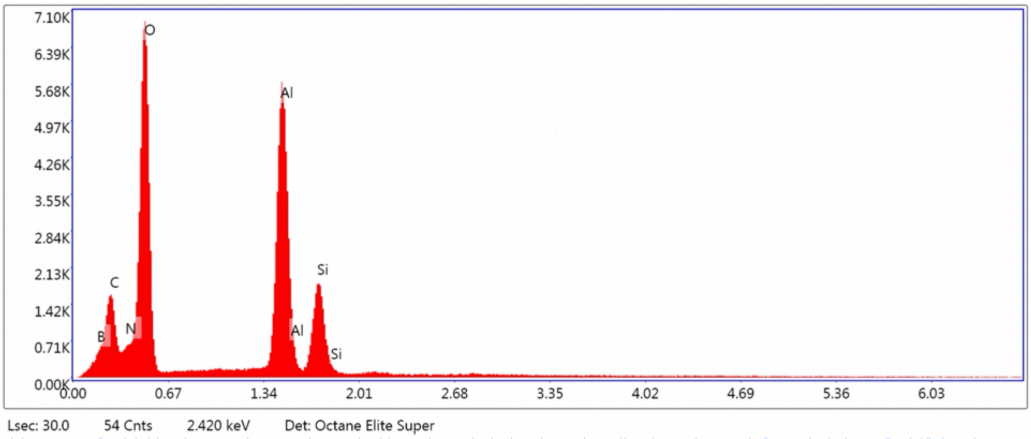

eZAF Smart Quant Results

Element	Weight %	Atomic %	Net Int.	Error %	Kratio	Z	A	F
B K	16.7	25.7	152.7	9.7	0.0500	0.7259	0.4128	1.0000
C K	12.5	17.3	398.4	8.1	0.0474	0.7581	0.5013	1.0000
N K	4.2	5.0	169.1	8.3	0.0194	0.7386	0.6255	1.0000
O K	26.5	27.5	1732.5	4.6	0.1500	0.7228	0.7842	1.0000
AlK	30.5	18.8	1861.6	2.6	0.2000	0.6526	1.0008	1.0037
SiK	9.7	5.7	538.7	4.3	0.0620	0.6707	0.9567	1.0011

72 h, 250 °C, with HOAc

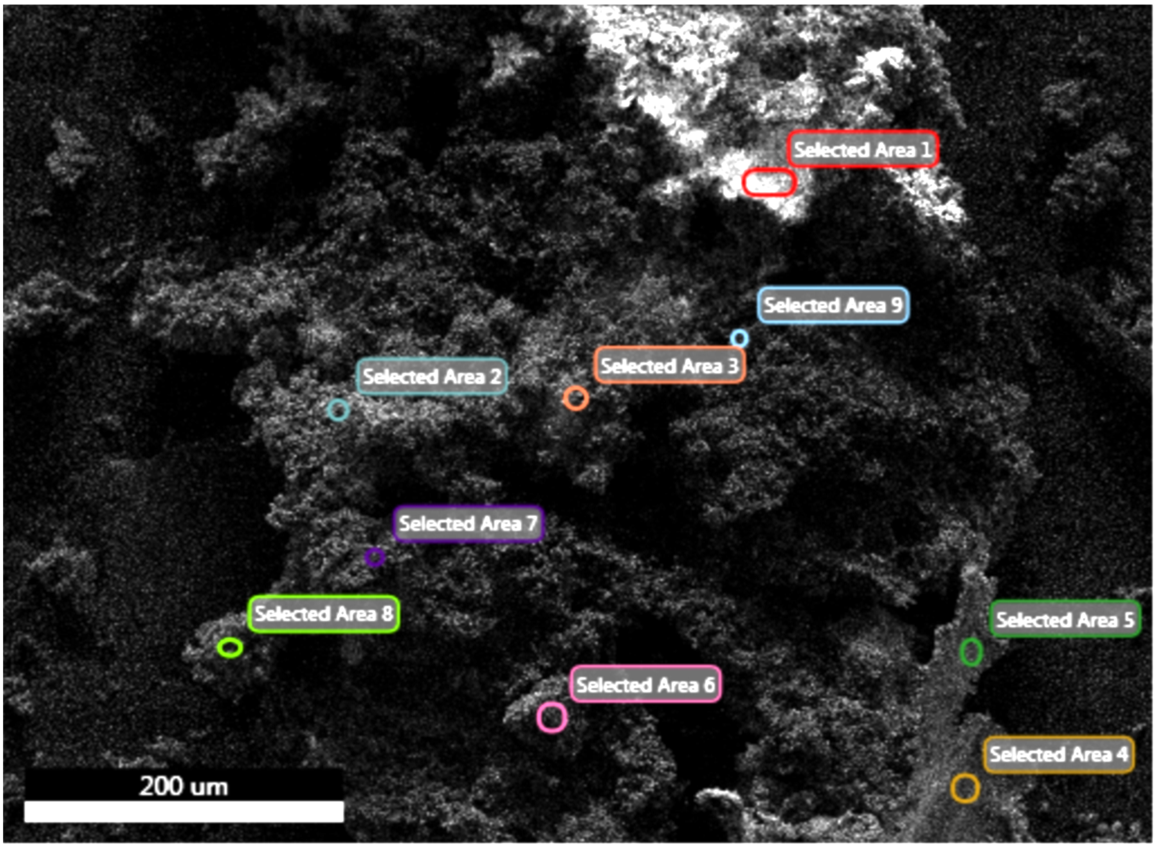

Area 1

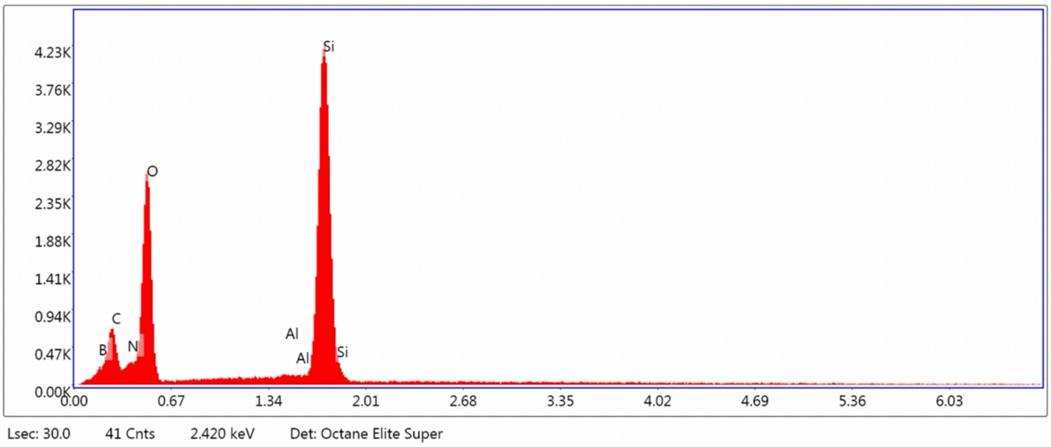

eZAF Smart Quant Results

Element	Weight %	Atomic %	Net Int.	Error %	Kratio	Z	A	F
B K	15.9	26.1	57.2	11.9	0.0389	0.7233	0.3383	1.0000
C K	11.3	16.6	150.2	9.6	0.0372	0.7547	0.4372	1.0000
N K	2.9	3.6	51.2	11.6	0.0122	0.7347	0.5818	1.0000
O K	20.0	22.2	606.9	5.2	0.1091	0.7185	0.7579	1.0000
AlK	1.1	0.7	34.3	10.4	0.0076	0.6481	1.0036	1.0412
SiK	48.8	30.8	1374.6	2.8	0.3288	0.6661	1.0106	1.0006

Area 2

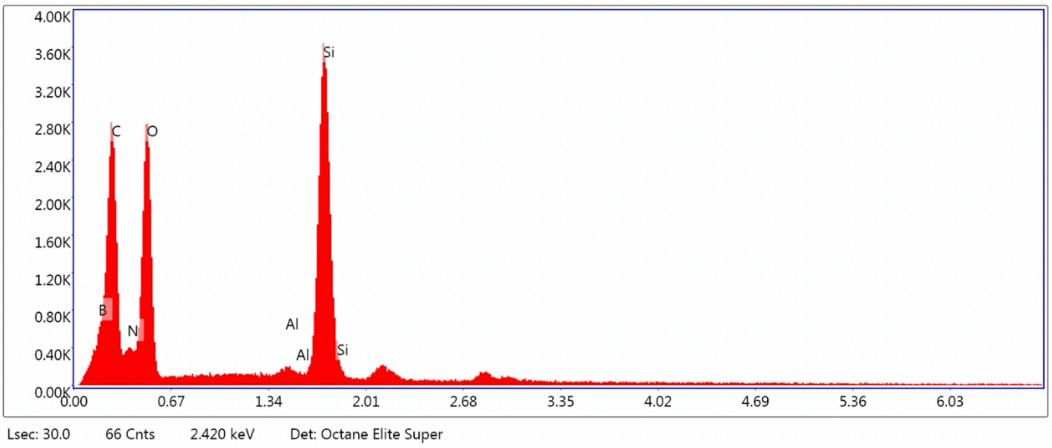

eZAF Smart Quant Results

Element	Weight %	Atomic %	Net Int.	Error %	Kratio	Z	A	F
B K	25.8	34.7	206.0	8.6	0.0896	0.7149	0.4853	1.0000
C K	29.1	35.3	664.8	8.2	0.1051	0.7475	0.4830	1.0000
N K	1.9	1.9	46.1	19.2	0.0070	0.7290	0.5151	1.0000
O K	14.4	13.1	636.4	6.2	0.0731	0.7137	0.7108	1.0000
AlK	1.5	0.8	69.6	7.4	0.0099	0.6451	1.0068	1.0280
SiK	27.3	14.1	1198.5	3.1	0.1833	0.6630	1.0121	1.0012

Area 3

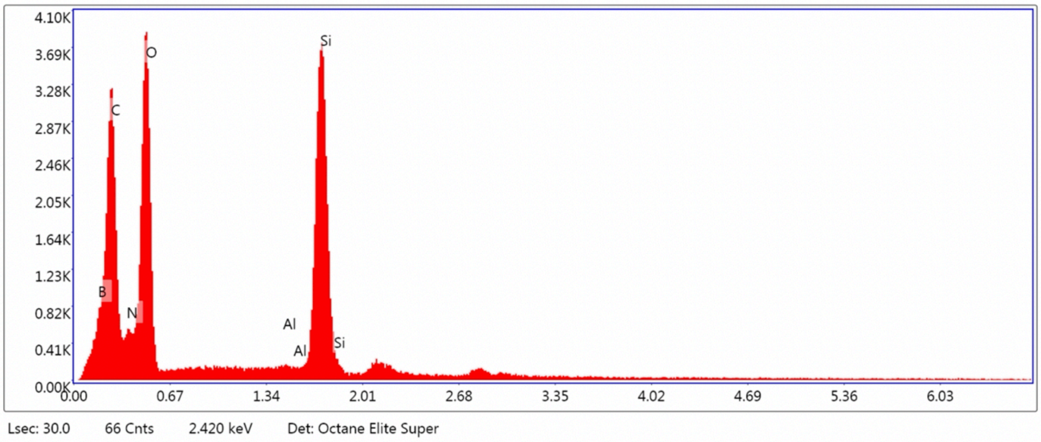

eZAF Smart Quant Results

Element	Weight %	Atomic %	Net Int.	Error %	Kratio	Z	A	F
B K	26.1	34.7	270.1	8.0	0.0965	0.7141	0.5169	1.0000
C K	28.3	33.8	810.8	7.9	0.1054	0.7469	0.4978	1.0000
N K	2.9	3.0	89.4	14.7	0.0112	0.7285	0.5275	1.0000
O K	17.5	15.7	945.7	5.8	0.0893	0.7133	0.7155	1.0000
AlK	0.8	0.4	46.0	10.1	0.0054	0.6448	1.0044	1.0259
SiK	24.3	12.4	1297.7	3.1	0.1631	0.6627	1.0118	1.0014

Area 4

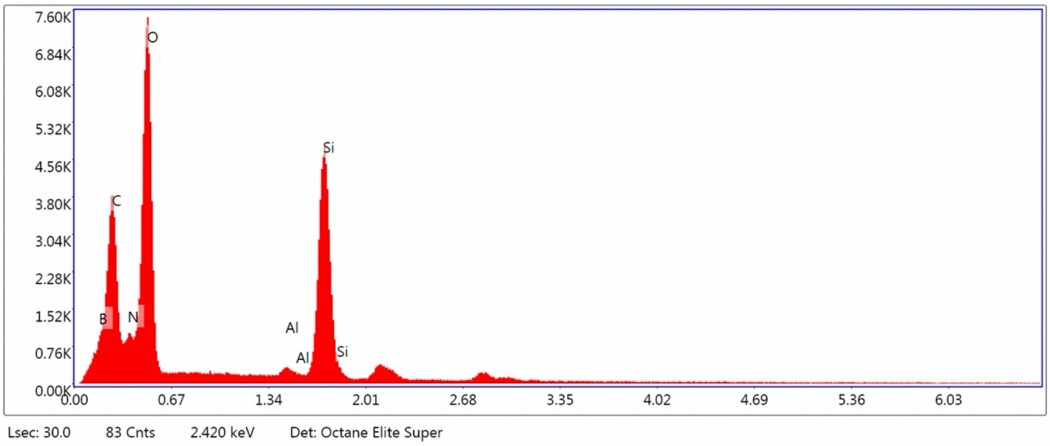

eZAF Smart Quant Results

Element	Weight %	Atomic %	Net Int.	Error %	Kratio	Z	A	F
B K	23.6	31.8	340.7	7.7	0.0883	0.7142	0.5241	1.0000
C K	23.6	28.6	972.3	7.6	0.0916	0.7469	0.5204	1.0000
N K	5.1	5.3	233.4	9.3	0.0212	0.7284	0.5672	1.0000
O K	24.3	22.1	1858.3	5.2	0.1273	0.7133	0.7339	1.0000
AlK	1.2	0.7	93.9	7.0	0.0080	0.6447	0.9994	1.0217
SiK	22.2	11.5	1628.2	2.9	0.1484	0.6626	1.0081	1.0015

Area 5

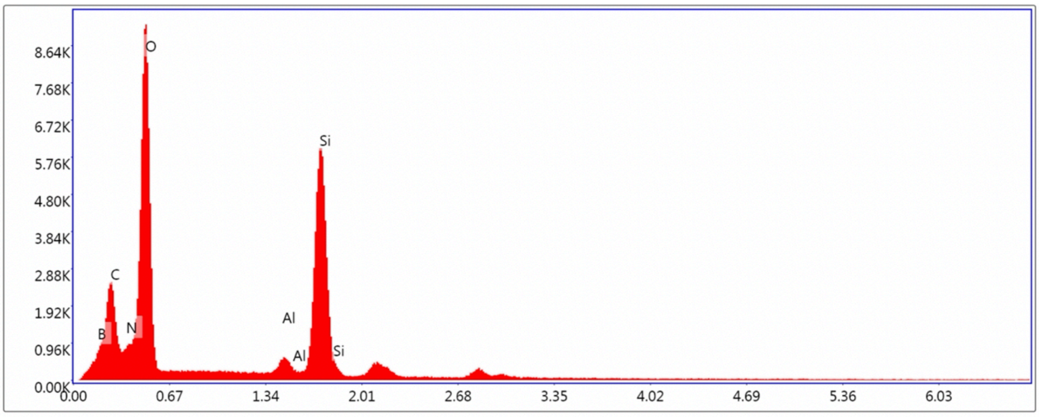

Lsec: 30.0 78 Cnts 2.420 keV Det: Octane Elite Super

eZAF Smart Quant Results

Element	Weight %	Atomic %	Net Int.	Error %	Kratio	Z	A	F
B K	20.4	29.3	259.1	8.7	0.0663	0.7172	0.4534	1.0000
C K	15.8	20.5	639.4	7.8	0.0595	0.7495	0.5015	1.0000
N K	5.1	5.6	249.5	8.0	0.0224	0.7306	0.6034	1.0000
O K	28.4	27.7	2290.1	4.8	0.1549	0.7151	0.7615	1.0000
AlK	2.4	1.4	188.9	5.2	0.0159	0.6460	0.9972	1.0232
SiK	27.9	15.4	2066.6	2.7	0.1860	0.6639	1.0043	1.0011

Area 6

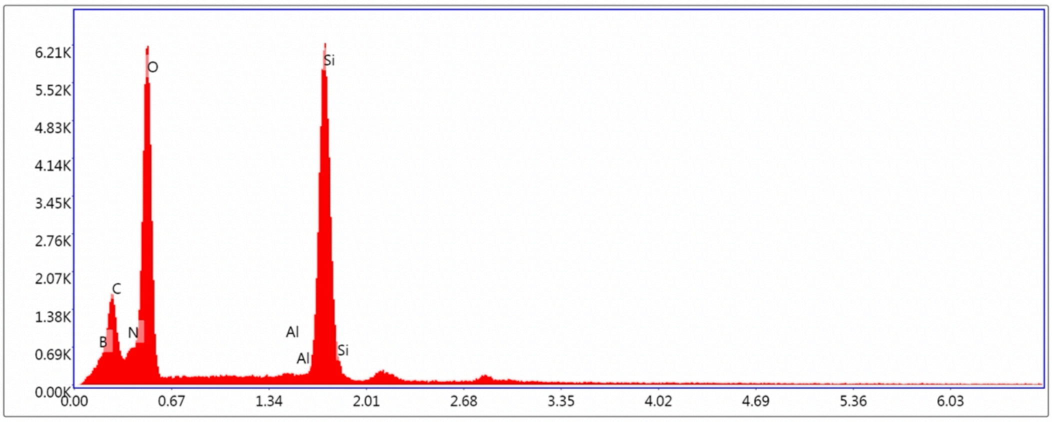

Lsec: 30.0 63 Cnts 2.420 keV Det: Octane Elite Super

eZAF Smart Quant Results

Element	Weight %	Atomic %	Net Int.	Error %	Kratio	Z	A	F
B K	18.6	28.0	152.6	9.9	0.0535	0.7190	0.4012	1.0000
C K	14.1	19.1	393.5	8.4	0.0502	0.7509	0.4754	1.0000
N K	4.6	5.3	161.2	8.7	0.0198	0.7316	0.5955	1.0000
O K	25.3	25.8	1478.7	4.9	0.1371	0.7159	0.7583	1.0000
AlK	0.9	0.6	53.7	9.6	0.0062	0.6464	0.9994	1.0321
SiK	36.7	21.3	1992.2	2.7	0.2457	0.6643	1.0083	1.0009

Area 7

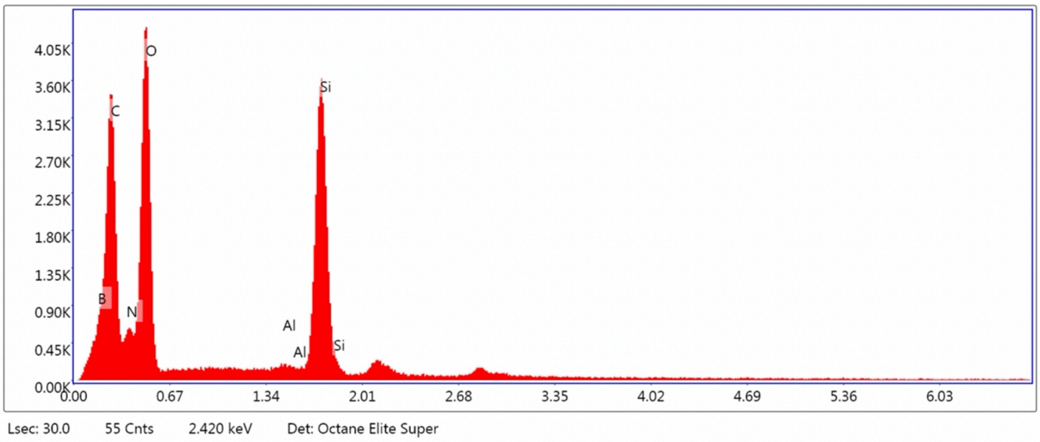

eZAF Smart Quant Results

Element	Weight %	Atomic %	Net Int.	Error %	Kratio	Z	A	F
B K	25.8	34.0	282.4	7.8	0.0982	0.7136	0.5334	1.0000
C K	29.0	34.4	870.0	7.8	0.1101	0.7465	0.5085	1.0000
N K	3.0	3.0	93.3	15.0	0.0114	0.7281	0.5299	1.0000
O K	18.8	16.7	1043.8	5.7	0.0960	0.7130	0.7174	1.0000
AlK	1.0	0.5	55.8	7.9	0.0064	0.6446	1.0035	1.0239
SiK	22.5	11.4	1234.0	3.1	0.1510	0.6624	1.0114	1.0015

Area 8

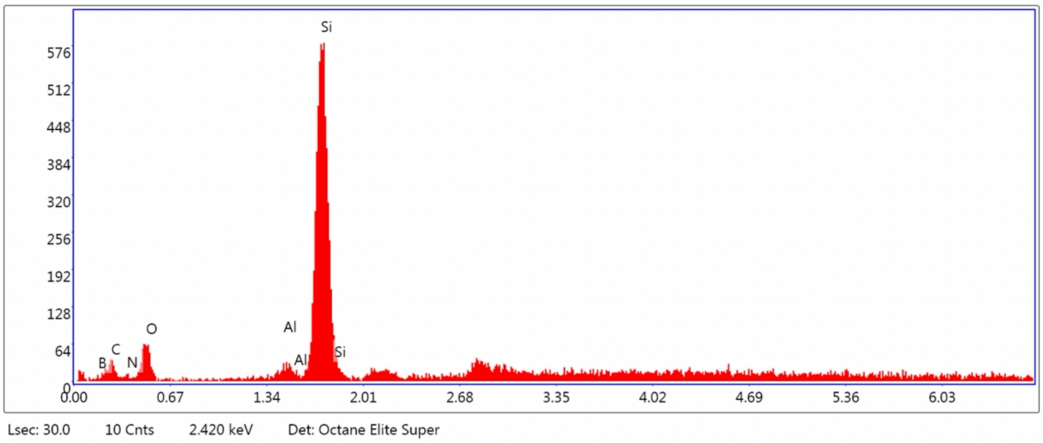

eZAF Smart Quant Results

Element	Weight %	Atomic %	Net Int.	Error %	Kratio	Z	A	F
B K	9.8	20.0	2.5	31.0	0.0180	0.7361	0.2497	1.0000
C K	5.5	10.1	6.0	22.5	0.0156	0.7661	0.3692	1.0000
N K	0.3	0.4	0.4	100.0	0.0011	0.7443	0.5484	1.0000
O K	5.2	7.2	14.9	11.9	0.0284	0.7271	0.7448	1.0000
AlK	3.3	2.7	9.9	13.1	0.0234	0.6540	1.0150	1.0566
SiK	75.8	59.5	204.2	3.8	0.5167	0.6722	1.0135	1.0002

Area 9

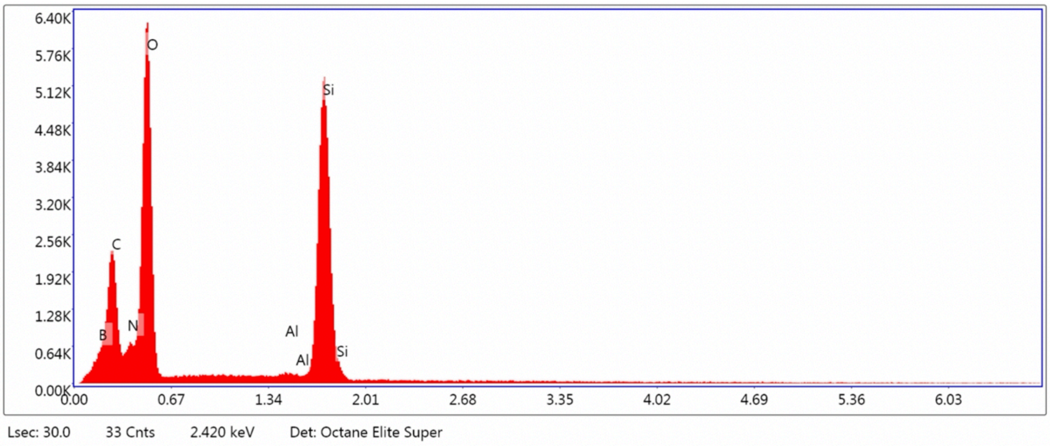

eZAF Smart Quant Results

Element	Weight %	Atomic %	Net Int.	Error %	Kratio	Z	A	F
B K	19.8	28.6	185.6	9.1	0.0629	0.7167	0.4425	1.0000
C K	18.4	23.9	556.7	7.9	0.0686	0.7489	0.4972	1.0000
N K	5.0	5.6	179.4	8.5	0.0213	0.7300	0.5837	1.0000
O K	24.8	24.2	1478.3	5.0	0.1324	0.7145	0.7467	1.0000
AlK	1.4	0.8	84.1	6.5	0.0093	0.6454	0.9993	1.0272
SiK	30.5	17.0	1715.3	2.7	0.2043	0.6633	1.0075	1.0011

3. Processing

3.1. Warm-pressing

The powders were consolidated by warm-pressing, a technique widely used for the compaction of powder-based polymers and polymer-derived materials.[6] The powders were introduced into a steel cavity with an inner diameter of 40 mm and were uniaxially compacted at 80 MPa. As release agent between the steel punches and the powder, Kapton foil was used. The temperature of the die was increased to 350 °C (heating rate 6 K min^{-1}), and the maximum temperature was held for 2 h. The pressure was adjusted accordingly when required. After cooling to room temperature, the pressure was reduced and the compacted part was removed from the mold.

3.2. Flexural strength evaluation

The flexural strength of the warm-pressed PI(TAPB-PMA) material was evaluated using a three-point flexural test setup, following EN ISO 178. Test specimens with dimensions of 5 x 1.6 x 30 mm were obtained using a diamond cutting disc. A total of

three specimens were tested until fracture using a crosshead speed of 1 mm min^{-1} (Universal testing machine Model 1474, Zwick, Germany).

1 M. Thommes, K. Kaneko, A. V. Neimark, J. P. Olivier, F. Rodriguez-Reinoso, J. Rouquerol and K. S. W. Sing, *Pure Appl. Chem.*, 2015, **87**, 1051–1069.
2 G. Li and Z. Wang, *Macromolecules*, 2013, **46**, 3058–3066.
3 Z. Wang, B. Zhang, H. Yu, G. Li and Y. Bao, *Soft Matter*, 2011, **7**, 5723–5730.
4 E. Rangel-Rangel, J. Weber, J. G. de la Campa, M. Iglesias and E. M. Maya, *Microporous Mesoporous Mater.*, 2017, **239**, 287–295.
5 Q. Fang, Z. Zhuang, S. Gu, R. B. Kaspar, J. Zheng, J. Wang, S. Qiu and Y. Yan, *Nat. Commun.*, 2014, **5**, 1–8.
6 T. Konegger, A. Liersch, C. Gierl and M. Scheffler, *Adv. Eng. Mater.*, 2013, **15**, 394–406.
7 H. D. Burks and T. L. St. Clair, *J. Appl. Polym. Sci.*, 1985, **30**, 2401–2411.
